# Epidemiology of yellow fever virus in humans, arthropods, and non-human primates in sub-Saharan Africa: A systematic review and meta-analysis

**DOI:** 10.1371/journal.pntd.0010610

**Published:** 2022-07-22

**Authors:** Martin Gael Oyono, Sebastien Kenmoe, Ngu Njei Abanda, Guy Roussel Takuissu, Jean Thierry Ebogo-Belobo, Raoul Kenfack-Momo, Cyprien Kengne-Nde, Donatien Serge Mbaga, Serges Tchatchouang, Josiane Kenfack-Zanguim, Robertine Lontuo Fogang, Elisabeth Zeuko’o Menkem, Juliette Laure Ndzie Ondigui, Ginette Irma Kame-Ngasse, Jeannette Nina Magoudjou-Pekam, Arnol Bowo-Ngandji, Seraphine Nkie Esemu, Lucy Ndip

**Affiliations:** 1 Centre for Research on Health and Priority Pathologies, Institute of Medical Research and Medicinal Plants Studies, Yaounde, Cameroon; 2 Laboratory of Parasitology and Ecology, Department of Animal Biology and Physiology, University of Yaounde I, Yaounde, Cameroon; 3 Department of Microbiology and Parasitology, University of Buea, Buea, Cameroon; 4 Virology Department, Centre Pasteur of Cameroon, Yaounde, Cameroon; 5 Centre for Food, Food Security and Nutrition Research, Institute of Medical Research and Medicinal Plants Studies, Yaounde, Cameroon; 6 Medical Research Centre, Institute of Medical Research and Medicinal Plants Studies, Yaounde, Cameroon; 7 Department of Biochemistry, The University of Yaounde I, Yaounde, Cameroon; 8 Epidemiological Surveillance, Evaluation and Research Unit, National AIDS Control Committee, Douala, Cameroon; 9 Department of Microbiology, The University of Yaounde I, Yaounde, Cameroon; 10 Scientific Direction, Centre Pasteur of Cameroon, Yaounde, Cameroon; 11 Department of Animal Biology, University of Dschang, Dschang, Cameroon; 12 Department of Biomedical Sciences, University of Buea, Buea, Cameroon; University of Pittsburgh, UNITED STATES

## Abstract

Yellow fever (YF) has re-emerged in the last two decades causing several outbreaks in endemic countries and spreading to new receptive regions. This changing epidemiology of YF creates new challenges for global public health efforts. Yellow fever is caused by the yellow fever virus (YFV) that circulates between humans, the mosquito vector, and non-human primates (NHP). In this systematic review and meta-analysis, we review and analyse data on the case fatality rate (CFR) and prevalence of YFV in humans, and on the prevalence of YFV in arthropods, and NHP in sub-Saharan Africa (SSA). We performed a comprehensive literature search in PubMed, Web of Science, African Journal Online, and African Index Medicus databases. We included studies reporting data on the CFR and/or prevalence of YFV. Extracted data was verified and analysed using the random effect meta-analysis. We conducted subgroup, sensitivity analysis, and publication bias analyses using the random effect meta-analysis while I^2^ statistic was employed to determine heterogeneity. This review was registered with PROSPERO under the identification CRD42021242444. The final meta-analysis included 55 studies. The overall case fatality rate due to YFV was 31.1% (18.3–45.4) in humans and pooled prevalence of YFV infection was 9.4% (6.9–12.2) in humans. Only five studies in West and East Africa detected the YFV in mosquito species of the genus *Aedes and in Anopheles funestus*. In NHP, YFV antibodies were found only in members of the *Cercopithecidae* family. Our analysis provides evidence on the ongoing circulation of the YFV in humans, *Aedes* mosquitoes and NHP in SSA. These observations highlight the ongoing transmission of the YFV and its potential to cause large outbreaks in SSA. As such, strategies such as those proposed by the WHO’s Eliminate Yellow Fever Epidemics (EYE) initiative are urgently needed to control and prevent yellow fever outbreaks in SSA.

## Author summary

Yellow fever, one of the most feared lethal zoonotic disease is re-emerging as a public health threat to tropical and sub-tropical countries of South America and Africa. Despite the existence of an effective yellow fever vaccine that is administered through mass vaccination campaigns and in routine immunization programs. against this disease, the mortality remains very high during the outbreak of yellow fever in several sub-Saharan African (SSA) countries. It is necessary to have accurate epidemiological data on YFV infection, in order to prioritize the policies, funding for public health interventions, and health-care planning. Our study is the first systematic review and meta-analysis with data provided on the case fatality rate (CFR) and prevalence of YFV in humans, and prevalence of YFV in arthropods, non-human primates (NHP), and other animal species in SSA. Broadly, the study shows that the CFR and prevalence of YFV in humans were relatively high and low respectively. Furthermore, mosquitoes of the genus *Aedes* and *Anopheles funestus* were the main vectors of YFV. Finally, only NHP of the *Cercopithecidae* family were the reservoirs of the YFV in SSA. These data provide evidence on the ongoing circulation of the YFV in SSA and the possibility of the large outbreaks YFV in SSA. Author suggests that preventive strategies should be promoted by educating and raising people’s awareness about YFV infection and strengthening practitioners ‘capacities towards adequate diagnosis and proper management of this infection in SSA.

## Introduction

Today and throughout history, animals have been an important source of pathogens transmitted to humans [1]. Between 1940 and 2004, over 60% of emerging infectious diseases in humans were due to pathogens from wildlife or domestic animals [2]. Some animal pathogens must be transmitted through an insect vector in order to infect humans [3,4]. A good example of such an animal pathogen requiring an insect vector to infect humans is the yellow fever virus (YFV) that causes yellow fever (YF) in humans. The YFV is an enveloped, positive-sense, single-stranded RNA virus that belongs to the genus of *Flavivirus* of the family Flaviviridae [5].

In a majority of humans, infection with the YFV may be asymptomatic or present with mild fever, headache, muscle pain and nausea. However, in about 20% of infected humans, the infection will progress to a severe form characterized by high fever, bleeding, jaundice, shock and, multiorgan failure and about 50% of these severe patients die within 7 to 10 days [6,7]. According to the World Health Organization (WHO), each year, about 200,000 YF cases and 30,000 deaths are reported of which nearly 90% of cases and death occur in Africa [8]. Clinically, YF presents with signs and symptoms similar to those of other diseases such as viral hepatitis and malaria and could easily be misdiagnosed for these diseases. As such, the actual number of YF cases could be several times higher than the reported number of cases [9].

YF is endemic in tropical and sub-tropical countries of South America and Africa [10]. In Africa, the YFV is spread through three transmission cycles: the sylvatic (or jungle) cycle, the intermediate (savannah) cycle and the urban cycle [11]. The sylvatic cycle is the primary cycle and spread of the YFV occurs between non-human primates (NHP) as reservoir hosts and forest dwelling mosquitoes such as *Aedes africanus*, *Aedes opok*, *Aedes simpsoni*, *Aedes luteocephalus*, *Aedes taylori*, *Aedes vittatus*. The YFV can then be passed from NHPs to humans when they visit or work in the jungle. The savannah cycle involves transmission of the YFV by an anthropo-zoophilic mosquito such as *Aedes albopictus* to humans living or working at the fringes of the jungle area. *Aedes albopictus* feeds on both animals and humans and serves as a bridge vector transferring YFV from animals to humans. In the urban cycle, domestic mosquitoes such as *Aedes aegypti* facilitates human-to-human transmission of the YFV [11–13].

The existence of an effective YF vaccine since the 1930s has greatly helped to contain YF outbreaks in Africa and beyond. However, since the mid-2000s, an upsurge in YFV transmission events have been reported throughout YF endemic countries especially in Africa [14–16]. Giving this unusual resurgence of YF transmission and the likelihood of major outbreaks, information on the burden and prevalence of YF in Africa is necessary for the development and deployment of counter measures [15]. We conducted a systematic review and meta-analysis to provide information in sub-Saharan Africa (SSA) on the prevalence and case fatality rate of YF in humans and on the prevalence of YF in arthropods and NHP.

## Methods

### Protocol and inclusion criteria

This systematic review was designed and conducted using the Preferred Reporting Items for Systematic Reviews and Meta–analyses (PRISMA) checklist (S1 Table) [17]. The protocol of this systematic review was published in the international database PROSPERO under the identification CRD42021242444. As this review reports on previously published data, ethical clearance was not required.

In this review, we considered cross-sectional studies, community and hospital-based studies and outbreak reports carried out in SSA. We included studies that reported on the prevalence and case fatality rate of YFV in humans, arthropods, NHP, and other animal species in SSA. Humans were further classified according to age, gender, disease state (healthy individuals and suspected YFV cases) and grouped according to the inclusion criteria of the selected studies. Arthropods were mosquitoes of the Diptera order. Other animal species and NHP were classified into their specific taxonomic orders.

To determine YF prevalence within the human populations, we included all studies reporting i) the detection of YFV specific IgM, ii) the detection of YFV specific IgG and/or IgM, iii) detection of YFV specific neutralizing antibodies, iv) detection of YFV by RT-PCR or viral isolation, v) detection of YFV specific antigens. As such, we included studies that utilize a wide range of methods for detection such as indirect immunofluorescence, complement fixation, culture, RT-PCR or ELISA, next-generation sequencing, or western blot.

Studies carried out of SSA or with inappropriate study design (comments, case reports, reviews, systematic reviews, and meta-analyses) as well as studies using positive YF samples or with sample size ≤ 10 were excluded. All studies from which data on YFV prevalence and case fatality rate (CFR) could not be obtained were also excluded. Studies published in neither the English nor French language or for which full text and abstract were either not available or could not be retrieved were also excluded.

### Article search method to identify studies for inclusion

A comprehensive strategy was designed to enable a search of relevant studies in the following databases: PubMed, Web of Science, African Journal Online, and African Index Medicus. These databases were searched for studies published in English or French languages from January 2000 until February 2021 and updated in March 2022 to have contemporary data on YF. Main search strategy (S2 Table) was developed and used to search the databases. The literature search was supplemented by a review of the reference list of identified articles to find additional potential studies. Names of SSA countries and regional groupings were also used to search for studies indexed under these names.

### Study selection and data extraction

After removing duplicates from the list of studies, titles and abstracts of the eligible studies were independently examined by two study authors (JETB and SK) for the selection of relevant studies. Data from the included studies was extracted using the online google form by 10 study authors and verified by SK and MGO. The extracted data were: the name of the first author, the year of publication, the study design, country, study period, sampling method, the study population (ill or apparently healthy humans, individual mosquitoes or negative pools, and NHP), age range of study population, YFV vaccine status, WHO Region, UNSD Region, country income level, YFV detection assay, YFV marker detected (virus, antigen, RNA, IgM or IgG), type of sample used for YFV detection, infection status (current, recent or past infections), sample size, number of positive for YFV, and number of deaths within YFV positive. For studies with less than 10 participants in animals and mosquitoes tested in pools and reporting a positive result, the names of the positive species were retained. Any difference in opinion with regards to the selection and inclusion of studies and extracted data were resolved by discussion, consensus, or by a third author.

### Evaluation of the quality of studies

The quality of the studies considered was assessed using the tool of Hoy et al. (S3 Table) [18]. This tool consists of 10 questions that evaluates the external and internal validity of the study. The expected answers for each question were “yes”, “no”, “unclear” and “not applicable” depending on the content of the articles. A score of 1 was assigned for all “yes” answers and 0 for the other ones. Articles with a total score of 0–3, 4–6, and 7–10 were considered to be respectively at high, moderate, and low risk of bias.

### Data synthesis

Data analysis was performed using R version 4.1.0 software [19]. We described without meta-analysis the positive YFV species of mosquitoes, NHP, and other animal species. The random effect model was performed to estimate combined prevalence of YFV and/or CFR in humans [20]. The Freeman-Tukey Double arcsine transformation was performed for the prevalence calculation [21]. The prevalence were represented as a forest plot with their corresponding 95% confidence intervals (CI). For the plot of the forest plot, a weighting according to the size of the sample was carried out to determine the size of the diamonds [22]. The Clopper-Pearson method was performed to calculate the 95% confidence interval of the prevalences [23,24]. Prevalences for future studies were determined by calculating a prediction interval [22,25]. The Cochran Q test and the *I*^*2*^ test statistic were used to measure the magnitude of heterogeneity between the included studies. The value of *I*^*2*^ more than 50%, was an indication of significant heterogeneity in the studies [26,27]. Sources of heterogeneity were investigated by subgroup analysis, and the sensitivity analysis that included only cross-sectional and low risk of bias studies was performed. Visual inspection of a funnel plot and the Egger test were used to estimate the risk of publication bias [28].

## Results

### Study selection

**Fig 1:**
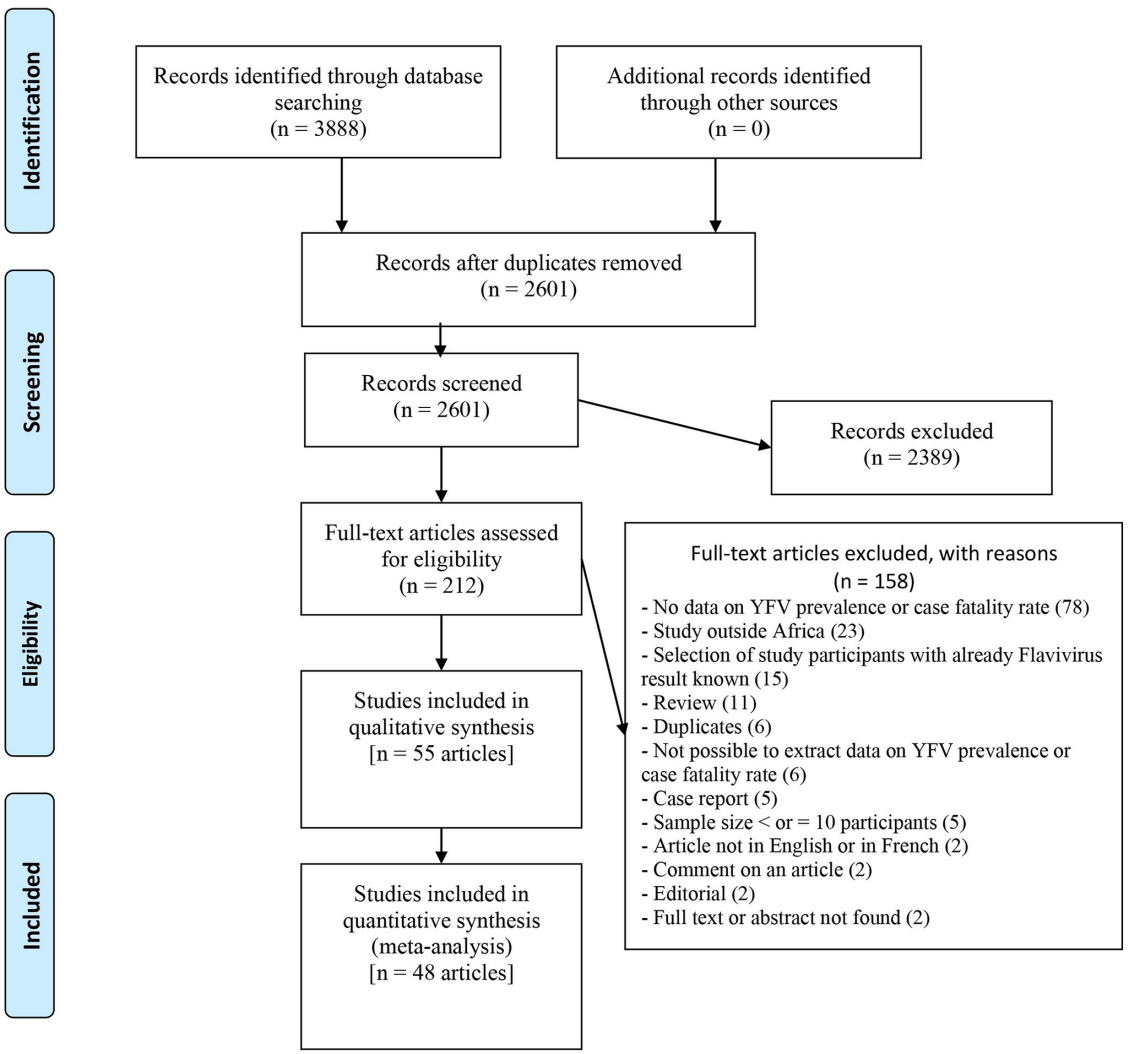
PRISMA flow diagram.

The literature search through databases provided a total of 3888 potentially relevant articles. After removing 1287 duplicate articles and excluding 2389 articles based on a careful review of the titles and abstracts, the remaining 212 articles were assessed for eligibility. Of the 212 articles, 157 full text articles were excluded for multiple reasons with absence of data on YFV prevalence or case fatality rate being the predominant reason (Fig 1 and S4 Table). We include a final total of 55 articles (151 datapoint on prevalence and/or CFR) in the qualitative and quantitative synthesis for this review [3–9,14,29–75].

### Assessment of study quality

The majority of studies included were at moderate risk of bias, 119/151 (78.8%); a few had a low risk of bias 32/151 (21.2%), and none of the included studies had a high risk of bias (S5 Table).

### Baseline characteristics of included studies

The summary and individual data of the included studies are presented in S6 and S7 Tables. The studies were published between 2001 and 2022 and the participants were recruited between 1990 and 2021. From the 151 data reported, 4/151 (2.8%) data reported CFR of YFV in humans, 71/151 (45.1%) reported prevalence of YFV in humans, 65/151 (43.0%) in mosquitoes, 7/151 (4.9%) in NHP, and 4/151 (2.8%) in other animal species. Data were majority recorded on cross-sectional studies 135/151 (88.7%), non-probabilistic 137/151 (90.7%), prospective 134/151 (88.7%), multicenter 136/151 (90.1%), and community-based 115/151 (76.2%). The included studies were conducted in 18 SSA countries with the highest number of studies conducted in Senegal 43/151 (28.5%) and the Central African Republic 33/151 (21.9%). Countries in West Africa 63/151 (41.7%) and Central Africa (49/151; 32.5%) had the largest number of studies. The predominant detection assays used to detect YFV in the included studies were sandwich ELISA 38/151 (25.2%), real-time RT-PCR 37/151 (24.5%), and infection of cellular cultures 35/151 (23.2%). The majority of studies found current YFV infection evidenced by the detection of viral RNA or live virus 89/151 (58.9%) and recent infection evidenced by the detection of IgM antibodies 32/151 (21.2%). With respect to studies among humans 64/63 (98.4%), NHP 5/7 (71.4%) and other animals 4/4 (100%), most of them found YFV in serum sample.

### Prevalence of yellow fever virus in mosquitoes, non-human primates, and other animal species in sub-Saharan Africa

The mosquitoes tested in the pool were from the Culicidae family. Individually tested mosquitoes from the genus *Aedes* 28/65 (43.1%), *Culex* 14/65 (21.5%), and *Anopheles* 13/65 (20.0%) were predominantly represented. Only five articles reported the detection of YFV in individually tested mosquitoes with prevalence ranging from 0.0 to 12.0%. The mosquito species that were positive for YFV included *Aedes aegypti*, *Aedes africanus*, *Aedes centropunctatus*, *Aedes dalzieli*, *Aedes furcifer*, *Aedes Luteocephalus*, *Aedes mcintoshi*, *Aedes taylori*, *Aedes vittatus*, and *Anopheles funestus* [29,39,41,42,46]. All studies involving mosquitoes were detection of the ongoing presence of YFV either by virus isolation or RT-PCR. Mosquito samples positive for YFV were only reported in Senegal and Kenya. Only two articles reported the detection of YFV antibodies in NHP with prevalence ranging from 1.8 and 48.0%. The family of *Cercopithecidae* 6/7 (85.7%) was the most represented NHP. YFV antibodies were detected in Gabon and in the Central African Republic in *Cercopithecidae* of the species Mandrills, *Chlorocebus*, *Cercopitheque*, *Cynocephalus*, and *Erythrocebus* [4,70]. None of the studies detected the YFV RNA (current infection) in NHP tested. Other animal species studied to date belong to the order of *Artiodactyla* 2/4 (50%), *Chiroptera* 1/4 (25.0%) and Proboscidea 1/4 (25.0%). Only one sample from bats in eastern Africa (Uganda) was found to be positive for YFV antibodies [51]. No sample from other animal species (Buffalo, Duike and Elephant) from countries in Central Africa region were positive for YFV or YF antibodies.

## Results of the meta-analysis

### Case Fatality Rate of Yellow Fever Virus infection in humans in sub-Saharan Africa

**Fig 2:**
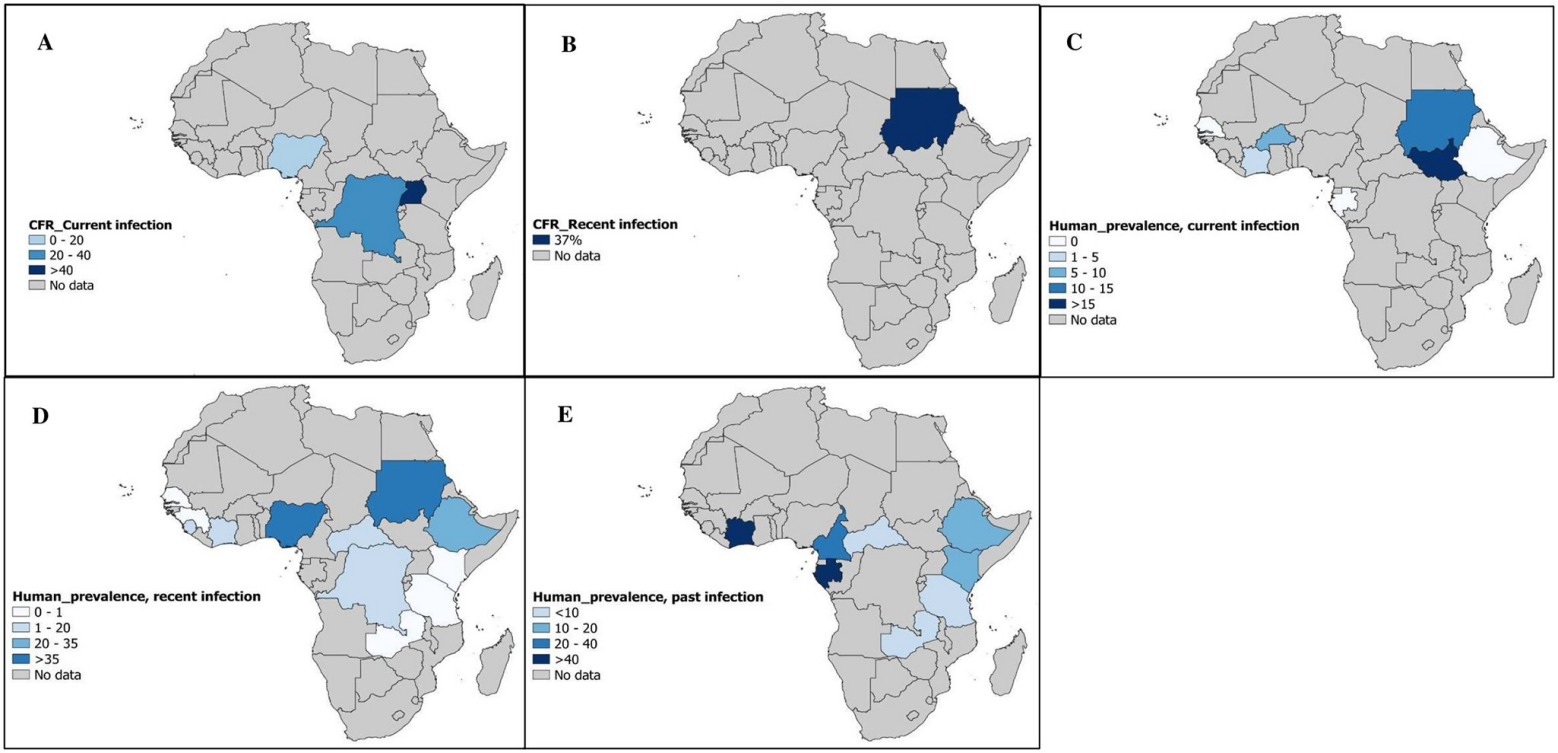
Case fatality rate and prevalence estimate of yellow fever virus in humans in sub-Saharan Africa. The letters (A and B) show the case fatality rate in humans with current and recent yellow fever virus exposures, respectively. The letters (C, D, and E) denote current, recent and past yellow fever virus exposures, respectively. The base map was taken from (https://www.naturalearthdata.com/) and modified with Qgis software.

**Fig 3:**
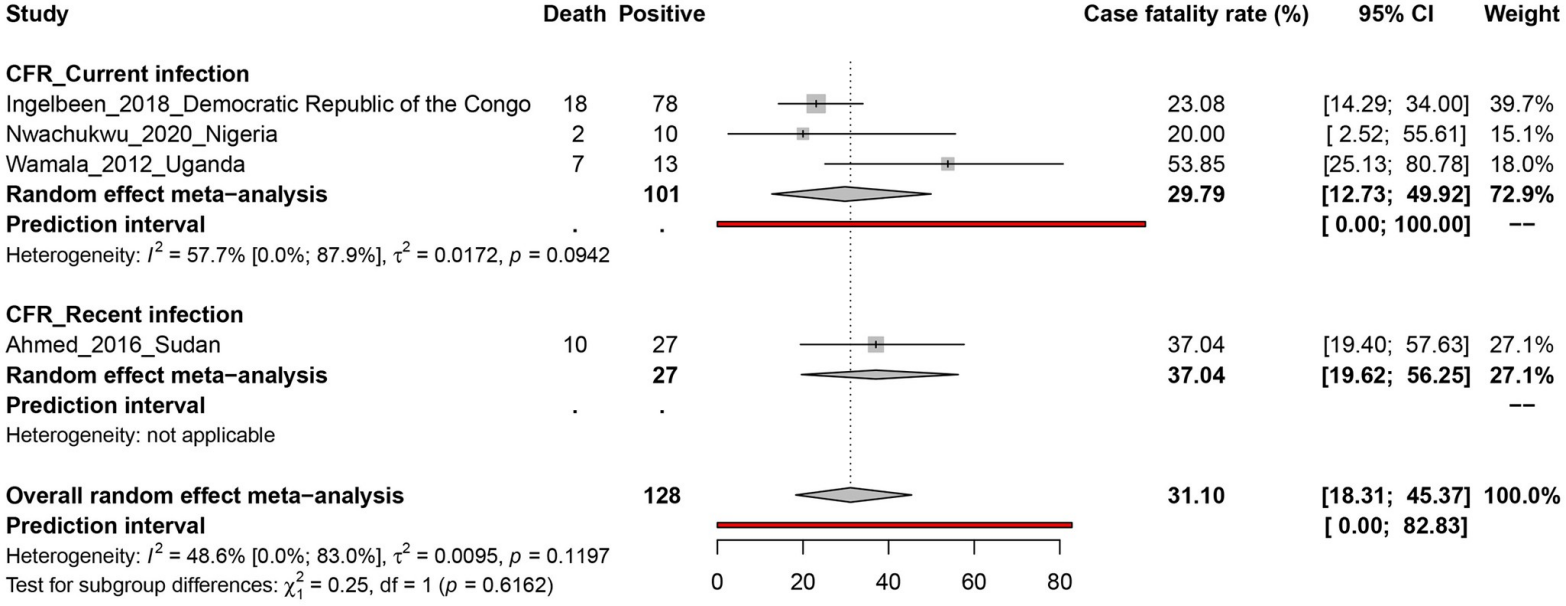
Case fatality rate estimate of yellow fever virus infections in humans in sub-Saharan Africa.

A case fatality rate of YFV infection was recorded in 4 studies conducted in four Africans countries: Democratic Republic of the Congo [76], Nigeria [62], Uganda [5] and Sudan [6] (Fig 2a and 2b). A total of 128 YFV suspected cases were recruited in the 4 studies giving an overall CFR of 31.1% (95% IC: 18.3–45.4) and data presented no heterogeneity (*I*^*2*^ = 48.6%, [95% CI = 0.0%–83.0%], *P* = 0.1197) (Fig 3). This estimated CFR of YFV varied across infection status with 29.8% [95% CI = 12.7–49.9] in people with current infection, and 37.0% [95% CI = 19.6–56.6] in people with recent infection. Based on the funnel plot (S1 Fig) and Egger’s regression test, there was a good symmetry and no evidence of potential publication bias (*P* = 0.382) for determining the CFR of YFV in humans.

### Prevalence of Yellow Fever Virus infection in humans in sub-Saharan Africa

**Fig 4:**
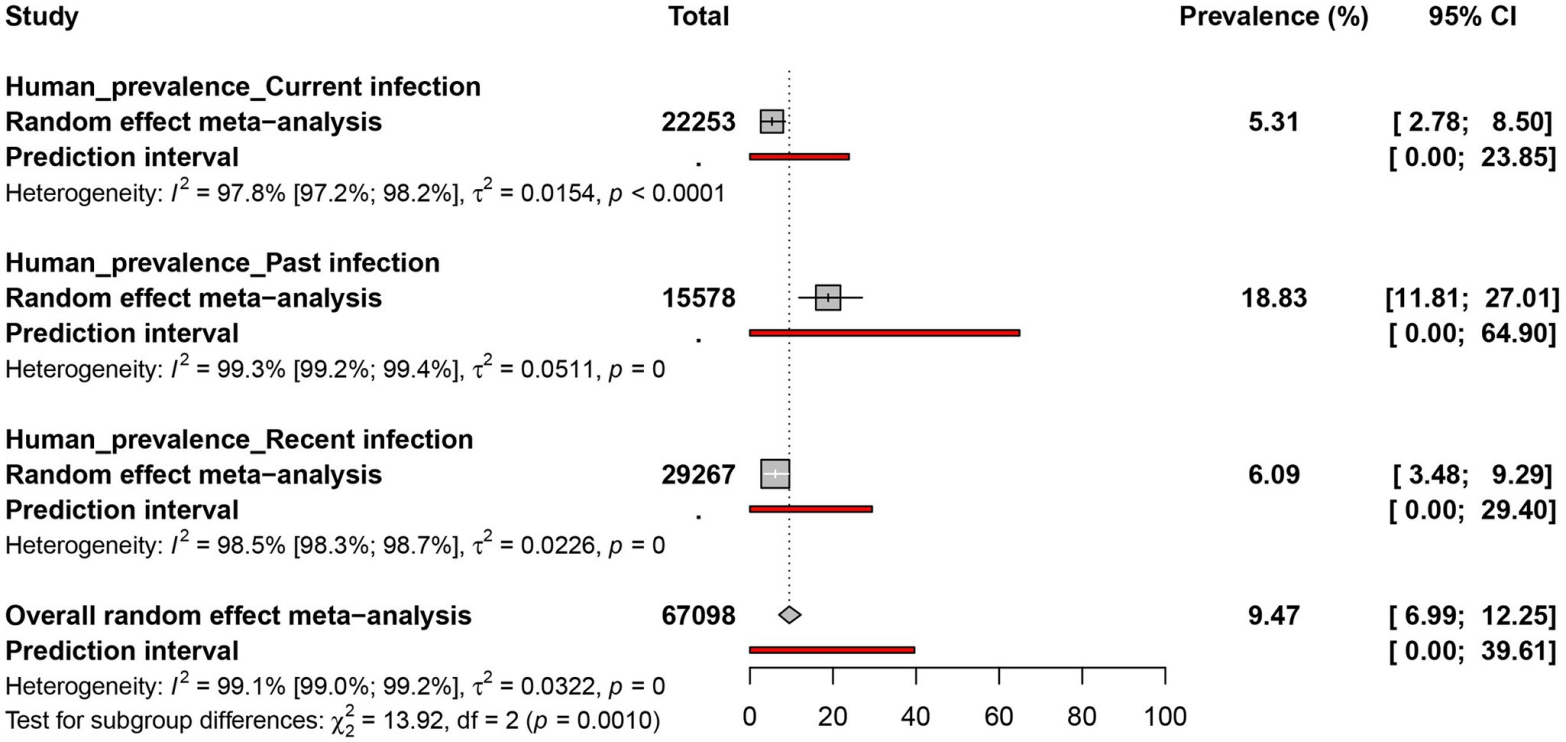
Prevalence estimates of yellow fever virus infections in humans in sub-Saharan Africa.

Studies on humans recruited mostly YFV suspected cases 22/71 (34.4%), apparently healthy individuals 22/71 (31.0%) and febrile patients 14/71 (19.7%). None of the studies considered, reported on the vaccination status of the enrolled participants. The overall prevalence of YFV recorded in 67098 human participants recruited from 71 datapoints prevalence was 9.4% (95% CI = 6.9–12.2) with a substantial heterogeneity between studies (*I*^2^ = 99.1% [95% CI = 99.0%–99.2%], p <0.001) (Figs 2c, 2d, 2e and 4, and S2 Fig). Regardless of the type of infection, the prevalence of YFV was 18.8% (95% CI = 11.8–27.0; 15578 participants), 6.0% (95% CI = 3.4–9.2; 29267 participants), and 5.3% (95% CI = 2.7–8.5; 22053 participants) in human participants with past, recent, and current infection respectively. Funnel plot (S3 Fig) and Egger’s regression test (Table 1) showed the existence of publication bias for studies of all types of YFV infections (p<0.001).

**Table 1:** Summary of meta-analysis results for case fatality rate and prevalence of yellow fever virus in humans in sub-Saharan Africa.

	Prevalence. % (95%CI)	95% Prediction interval	N Studies	N Participants	^¶^H (95%CI)	^§^I^2^ (95%CI)	P heterogeneity
YFV case fatality rate in humans							
Current infection							
Overall	29.8 [12.7–49.9]	[0–100]	3	101	1.5 [1–2.9]	57.7 [0–87.9]	0.094
Cross-sectional	23.1 [14.3–33.1]	NA	1	78	NA	NA	1
Recent infection							
Overall	37 [19.6–56.2]	NA	1	27	NA	NA	1
YFV prevalence in humans							
Current infection							
Overall	5.3 [2.8–8.5]	[0–23.8]	19	22253	6.7 [6–7.4]	97.8 [97.2–98.2]	<0.001
Cross-sectional	3.6 [1.3–6.8]	[0–20.8]	12	21313	7.7 [6.8–8.8]	98.3 [97.8–98.7]	<0.002
Low risk of bias	0.8 [0–3.1]	[0–19.2]	4	18272	6.8 [5.3–8.9]	97.9 [96.4–98.7]	<0.003
Past infection							
Overall	18.8 [11.8–27]	[0–64.9]	22	15578	11.7 [10.9–12.6]	99.3 [99.2–99.4]	<0.001
Cross-sectional	18 [11.2–26]	[0–62.4]	21	14973	11.3 [10.5–12.2]	99.2 [99.1–99.3]	<0.002
Low risk of bias	12.7 [5–23.3]	[0–65.7]	13	10297	13.5 [12.4–14.6]	99.4 [99.3–99.5]	<0.003
Recent infection							
Overall	6.1 [3.5–9.3]	[0–29.4]	30	29267	8.3 [7.7–8.9]	98.5 [98.3–98.7]	<0.001
Cross-sectional	4.3 [2.1–7.2]	[0–24.1]	25	28353	8.2 [7.5–8.9]	98.5 [98.2–98.7]	<0.002
Low risk of bias	2.1 [0.9–3.9]	[0–11.1]	11	24532	6.4 [5.5–7.5]	97.6 [96.7–98.2]	<0.003

CI: confidence interval; N: Number; 95% CI: 95% Confidence Interval; NA: not applicable.

^¶^H is a measure of the extent of heterogeneity. a value of H = 1 indicates homogeneity of effects and a value of H >1indicates a potential heterogeneity of effects.

^§^: I2 describes the proportion of total variation in study estimates that is due to heterogeneity. a value > 50% indicates presence of heterogeneity

### Subgroup analysis of meta-analysis results for case fatality rate and prevalence of yellow fever virus in humans in sub-Saharan Africa

Subgroup analyses of case fatality rate and prevalence of YFV in humans, mosquitoes, NHP, and other animal species in SSA is summarize in S8 Table and Fig 2. Analysis of the data showed that YFV CFR was significantly higher in community-based studies (42.3%; 95%CI: 27.0–58.4; p = 0.031). Community-based studies (10.4%; 95%CI: 6.9–14.4; p = 0.015), conducted in Nigeria (44.7%; 95%CI: 36.3–53.2), Cameroon (26.1%; 95%CI: 12.0–43.1), and Sudan (25.6%; 95%CI: 5.8–52.5; p < 0.015), recruiting hospitalized participants (27.1%; 95%CI: 11.3–46.5; p = 0.014), suspected YFV cases (11.4%; 95%CI: 7.7–15.7), YFV positive case contacts (10.6%; 95%CI: 0.0–34.2), and apparently healthy individuals (10.8%; 95%CI: 6.1–16.7; p < 0.001) were more likely to show higher YFV prevalence.

## Discussion

The present study is the first systematic review and meta-analysis on the prevalence and CFR of YFV in humans, and YFV prevalence in arthropods, NHP, and other animal species in SSA. Overall, our analysis reports a computed pooled CFR estimate due to YF of 31.1% in humans and an overall prevalence of 9.4% of YFV in humans. Mosquitoes positive for YFV included several species of the genus *Aedes* and *Anopheles funestus*. Only NHP of the Cercopithecidae family showed serological evidence of exposure to YFV.

The estimated CFR of YFV in humans identified in this review is consistent with that recently reported in a global review with a CFR of 36% for Africa [77]. Such a high CFR could be due to delays or deterrents in seeking care during the early less sever phase of the disease or delayed clinical diagnosis of cases [62,76]. It should also be noted that African population is more at risk of contracting the yellow fever virus and of developing severe forms and death due to a low rate of vaccination coverage and daily activities that bring them closer to vectors such as agriculture, livestock, hunting, and deforestation [77–80]. Also, the existence in Africa of other health conditions such as malnutrition, tuberculosis, malaria and, HIV are other factors that could be associated with this high of YFV CFR [81–83]. About 1/5^th^ (18.8%) of sampled human participants included in this review had IgG antibodies against YFV (past infection). YF IgG antibodies could be naturally acquired following an infection with the virus or following vaccination with the YF vaccine [84]. It is unclear if participants in the included studies had received the YF vaccine as participant’s vaccination history was not reported in most studies. However, the estimated seroprevalence level is slightly reflective of naturally acquire IgG antibodies as the value is comparable to values reported by individual studies conducted on non-vaccinated persons in subgroup analysis. Even so, we cannot rule out the contribution of the YF vaccine on the seroprevalence levels. Most of the included studies were conducted in countries with moderate to high levels of YF vaccine coverage [85]. Despite reports of a good vaccine coverage in SSA including countries incorporated in this review, YF infection continues to persist. The prevalence rates of current and recent infection were 5.3% and 6.0% respectively. This prevalence levels could even be higher if not of the inherent limitation in detecting YF viral RNA and/or antigen (current infection) and YF IgM antibodies (recent infection). Identifying these infection biomarkers is totally dependent on the timing of sample collection and if the studied area is endemic to other flaviviruses such as Dengue, Zika [86]. YF viral RNA and/or antigen can be detected in serum of symptomatic patients only during the first 7 days of illness or for longer periods in severe cases (30% of patients). As such, in the 70% of patients presenting mild symptoms, if this period of sample collection is missed, testing for viral RNA and/or antigen may not be clinically useful. Generally, YF diagnosis relies on the detection YF IgM antibodies as IgM antibodies can be detected for up to 3 months following infection. However, in patients with a prior history of infection with other flaviviruses, IgM antibodies may absent or present briefly (<1 month) thereby hampering IgM detection [87–89]. Among the included studies, high numbers of current and recent YF infections were predominantly detected in studies in Nigeria, Cameroon, and Sudan and among hospitalized patients who were most likely exhibiting severe symptoms.

Mosquitoes or the genus *Aedes* are the primary vectors responsible for the transmission of the YFV in all transmission cycles [13]. Surprisingly, despite evidence of YF infection in humans, very few of the included individual studies were able to identify YFV in mosquitoes. Broadly, mosquitoes of the *Aedes* genus specifically *Aedes africanus*, *Aedes furcifer*, *Aedes luteocephalus*, *Aedes taylori*, *and Aedes vittatus* in rural settings and *Aedes aegypti* in an urban setting were found to be positive for YFV. The relatively low detection of YFV in mosquitoes could be due to the low viral load in mosquitoes making detection by direct isolation or RT-PCR very challenging. Also, collected mosquitoes need to be transported at low temperatures to prevent degradation of viral RNA [90]. New techniques and technological advances such as mosquito traps with inserted FTA nuclei acid preservation cards could help bypass some of these challenges in sampling mosquitoes for YF detection [91,92]. NHP are generally considered as competent reservoir hosts of the YFV and are responsible for maintaining the sylvatic YFV transmission cycle [13]. In this review, we found a low rate of YFV antibodies exposure among NHP. Among the NHP, YFV antibodies were detected in mandrills from Gabon and *Cercopithecidae* from the Central Africa Republic, although the number of studies on NHP were limited. The scanty evidence of acute and/or recent infection of YFV in NHP makes it difficult to understand their role as reservoir host and in maintaining the sylvatic and intermediate transmission cycle in SSA. Although antibodies to YFV were detected in a bat in Uganda, the role of bats as a reservoir could not be ascertained and this would probably be the result of a cross-reaction with another virus [89].

Overall, this systematic review and meta-analysis provides evidence on the ongoing circulation of the YFV in humans, *Aedes* mosquitoes and NHP in SSA. Our analysis reports on the prevalence of the YFV among the different studied populations. The high number of studies included in this review increases the accuracy of reported estimates. However, there are at least two limitations to our study. First, we observed substantial heterogeneity among the included studies that still existed even when subgroup analyses were done. Secondly, most of the reported pooled estimates had significant publication bias. Despite these limitations, our analyses revealed: the presence of YFV in humans with a relatively high CFR especially during outbreak, one family of NHP (Cercopithecidae) served as a potential reservoir host and *Aedes* species as main vector of YFV in SSA. These observations highlight the ongoing transmission of the YFV and its potential causing large outbreaks in SSA. As such, strategies such as those proposed by the WHO’s Eliminate Yellow Fever Epidemics (EYE) initiative are urgently needed to control and prevent YFV outbreaks [93].

## Supporting information

S1 Table Preferred reporting items for systematic reviews and meta-analyses checklist. (PDF)

S2 Table Search strategy in PubMed. (PDF)

S3 Table Items for risk of bias assessment. (PDF)

S4 Table Main reasons of exclusion of eligible studies. (PDF)

S5 Table Risk of bias assessment. (PDF)

S6 Table Characteristics of included studies. (PDF)

S7 Table Individual characteristics of included studies. (PDF)

S8 Table Subgroup analyses of case fatality rate and prevalence of yellow fever virus in humans in sub-Saharan Africa. (PDF)

S1 Fig Funnel chart for publications of the yellow fever virus case fatality rate in sub-Saharan Africa. (PDF)

S2 Fig Prevalence estimate of yellow fever virus infections in humans in sub-Saharan Africa. (PDF)

S3 Fig Funnel chart for publications of the yellow fever virus prevalence in humans in Africa. (PDF)

## References

[1] Taylor LH, Latham SM, Woolhouse ME. Risk factors for human disease emergence. Philosophical transactions of the Royal Society of London Series B, Biological sciences. 2001;356(1411):983–9. Epub 2001/08/23. doi: 10.1098/rstb.2001.0888 . 11516376 PMC1088493

[2] Jones KE, Patel NG, Levy MA, Storeygard A, Balk D, Gittleman JL, et al. Global trends in emerging infectious diseases. Nature. 2008;451(7181):990–3. Epub 2008/02/22. doi: 10.1038/nature06536 . 18288193 PMC5960580

[3] Mohamed N, Magzoub M, Mohamed REH, Aleanizy FS, Alqahtani FY, Nour BYM, et al. Prevalence and identification of arthropod-transmitted viruses in Kassala state, Eastern Sudan. Libyan J Med. 2019;14(1):1564511. Epub 2019/02/05. doi: 10.1080/19932820.2018.1564511 . 30716013 PMC6366427

[4] Kading RC, Borland EM, Cranfield M, Powers AM. Prevalence of antibodies to alphaviruses and flaviviruses in free-ranging game animals and nonhuman primates in the greater Congo basin. Journal of wildlife diseases. 2013;49(3):587–99. Epub 2013/06/20. doi: 10.7589/2012-08-212 . 23778608

[5] Wamala JF, Malimbo M, Okot CL, Atai-Omoruto AD, Tenywa E, Miller JR, et al. Epidemiological and laboratory characterization of a yellow fever outbreak in northern Uganda, October 2010-January 2011. International journal of infectious diseases: IJID: official publication of the International Society for Infectious Diseases. 2012;16(7):e536–42. Epub 2012/05/12. doi: 10.1016/j.ijid.2012.03.004 . 22575876

[6] Ahmed SS, Soghaier MA, Mohammed S, Khogali HS, Osman MM, Abdalla AM. Concomitant outbreaks of yellow fever and hepatitis E virus in Darfur States, Sudan, 2012. Journal of infection in developing countries. 2016;10(1):24–9. Epub 2016/02/02. doi: 10.3855/jidc.6342 . 26829534

[7] Gould LH, Osman MS, Farnon EC, Griffith KS, Godsey MS, Karch S, et al. An outbreak of yellow fever with concurrent chikungunya virus transmission in South Kordofan, Sudan, 2005. Transactions of the Royal Society of Tropical Medicine and Hygiene. 2008;102(12):1247–54. Epub 2008/05/27. doi: 10.1016/j.trstmh.2008.04.014 . 18502458

[8] Mengesha Tsegaye M, Beyene B, Ayele W, Abebe A, Tareke I, Sall A, et al. Sero-prevalence of yellow fever and related Flavi viruses in Ethiopia: a public health perspective. BMC public health. 2018;18(1):1011. Epub 2018/08/21. doi: 10.1186/s12889-018-5726-9 mc6092792. 30107830 PMC6092792

[9] Babaniyi OA, Mwaba P, Mulenga D, Monze M, Songolo P, Mazaba-Liwewe ML, et al. Risk assessment for yellow Fever in Western and north-Western provinces of zambia. Journal of global infectious diseases. 2015;7(1):11–7. Epub 2015/02/28. doi: 10.4103/0974-777X.150884 mc4338443. 25722614 PMC4338443

[10] Monath TP. Yellow fever: an update. The Lancet Infectious diseases. 2001;1(1):11–20. Epub 2002/03/02. doi: 10.1016/S1473-3099(01)00016-0 . 11871403

[11] Huang Y-JS, Higgs S, Horne KM, Vanlandingham DL. Flavivirus-mosquito interactions. Viruses. 2014;6(11):4703–30. doi: 10.3390/v6114703 MEDLINE:. 25421894 PMC4246245

[12] Onyango CO, Grobbelaar AA, Gibson GV, Sang RC, Sow A, Swaneopel R, et al. Yellow fever outbreak, southern Sudan, 2003. Emerging infectious diseases. 2004;10(9):1668–70. Epub 2004/10/23. doi: 10.3201/eid1009.030727 mc3320285. 15498174 PMC3320285

[13] Gardner CL, Ryman KD. Yellow fever: a reemerging threat. Clinics in laboratory medicine. 2010;30(1):237–60. Epub 2010/06/02. doi: 10.1016/j.cll.2010.01.001 . 20513550 PMC4349381

[14] Rachas A, Nakouné E, Bouscaillou J, Paireau J, Selekon B, Senekian D, et al. Timeliness of yellow fever surveillance, Central African Republic. Emerging infectious diseases. 2014;20(6):1004–8. Epub 2014/05/27. doi: 10.3201/eid2006.130671 mc4036780. 24857597 PMC4036780

[15] World Health Organisation. Eliminate Yellow fever Epidemics (EYE): a global strategy, 2017–2026. Releve epidemiologique hebdomadaire. 2017;92(16):193–204. Epub 2017/04/22. . 28429585

[16] Chen LH, Wilson ME. Yellow fever control: current epidemiology and vaccination strategies. Tropical diseases, travel medicine and vaccines. 2020;6:1. Epub 2020/01/16. doi: 10.1186/s40794-020-0101-0 mc6954598. 31938550 PMC6954598

[17] Moher D, Liberati A, Tetzlaff J, Altman DG, Group P. Preferred reporting items for systematic reviews and meta-analyses: the PRISMA Statement. Open Med. 2009;3(3):e123–30. Epub 2009/01/01. . 21603045 PMC3090117

[18] Hoy D, Brooks P, Woolf A, Blyth F, March L, Bain C, et al. Assessing risk of bias in prevalence studies: modification of an existing tool and evidence of interrater agreement. Journal of clinical epidemiology. 2012;65(9):934–9. Epub 2012/06/30. doi: 10.1016/j.jclinepi.2011.11.014 . 22742910

[19] Schwarzer G. meta: An R Package for Meta-Analysis. 2007;7:40–5.

[20] DerSimonian R, Laird N. Meta-analysis in clinical trials. Controlled clinical trials. 1986;7(3):177–88. Epub 1986/09/01. doi: 10.1016/0197-2456(86)90046-2 3802833

[21] Barendregt JJ, Doi SA, Lee YY, Norman RE, Vos T. Meta-analysis of prevalence. Journal of epidemiology and community health. 2013;67(11):974–8. Epub 2013/08/22. doi: 10.1136/jech-2013-203104 . 23963506

[22] Higgins JP, Thompson SG, Spiegelhalter DJ. A re-evaluation of random-effects meta-analysis. Journal of the Royal Statistical Society Series A, (Statistics in Society). 2009;172(1):137–59. Epub 2009/04/22. doi: 10.1111/j.1467-985X.2008.00552.x . 19381330 PMC2667312

[23] Agresti A, Coull BA. Approximate is Better than “Exact” for Interval Estimation of Binomial Proportions. The American Statistician. 1998;52(2):119–26. doi: 10.1080/00031305.1998.10480550

[24] Newcombe RG. Two-sided confidence intervals for the single proportion: comparison of seven methods. Statistics in medicine. 1998;17(8):857–72. Epub 1998/05/22. doi: 10.1002/(sici)1097-0258(19980430)17:8&lt;857::aid-sim777&gt;3.0.co;2-e 9595616

[25] Guddat C, Grouven U, Bender R, Skipka G. A note on the graphical presentation of prediction intervals in random-effects meta-analyses. Systematic reviews. 2012;1:34. Epub 2012/07/31. doi: 10.1186/2046-4053-1-34 . 22839660 PMC3552946

[26] Borenstein M, Hedges LV, Higgins JPT, Rothstein HR. Introduction to Meta-Analysis: John Wiley and Sons; 2009 2009/03/11/.

[27] Higgins JP, Thompson SG, Deeks JJ, Altman DG. Measuring inconsistency in meta-analyses. BMJ (Clinical research ed). 2003;327(7414):557–60. Epub 2003/09/06. doi: 10.1136/bmj.327.7414.557 . 12958120 PMC192859

[28] Egger M, Davey Smith G, Schneider M, Minder C. Bias in meta-analysis detected by a simple, graphical test. BMJ (Clinical research ed). 1997;315(7109):629–34. Epub 1997/10/06. doi: 10.1136/bmj.315.7109.629 9310563 PMC2127453

[29] Agwu EJ, Igbinosa IB, Isaac C. Entomological assessment of yellow fever-epidemic risk indices in Benue State, Nigeria, 2010–2011. Acta tropica. 2016;161:18–25. Epub 2016/05/18. doi: 10.1016/j.actatropica.2016.05.005 . 27189925

[30] Akelew Y, Pareyn M, Lemma M, Negash M, Bewket G, Derbew A, et al. Aetiologies of acute undifferentiated febrile illness at the emergency ward of the University of Gondar Hospital, Ethiopia. Tropical medicine & international health: TM & IH. 2022. Epub 2022/01/15. doi: 10.1111/tmi.13721 . 35029010

[31] Akoua-Koffi C, Akran V, kata Faye O, Grandadam M, Ekaza E, Kouassi KS, et al. Yellow fever and dengue fever serotype 3 viruses cocirculation in Côte d’Ivoire in 2008. African Journal of Pathology and Microbiology. 2014.

[32] Akoua-Koffi C, Diarrassouba S, Benie VB, Ngbichi JM, Bozoua T, Bosson A, et al. Inquiry into a fatal case of yellow fever in Cote d’lvoire in 1999. Bulletin De La Societe De Pathologie Exotique. 2001;94(3):227–30. CCC:000171401500001. 11681215

[33] Akoua-Koffi C, Ekra KD, Kone AB, Dagnan NS, Akran V, Kouadio KL, et al. [Detection and management of the yellow fever epidemic in the Ivory Coast, 2001]. Medecine tropicale: revue du Corps de sante colonial. 2002;62(3):305–9. Epub 2002/09/25. . 12244930

[34] Alhakimi HA, Mohamed OG, Khogaly HSE, Arafa KAO, Ahmed WA. Epidemiological, Clinical and Entomological Characteristics of Yellow Fever Outbreak in Darfur 2012. AIMS public health. 2015;2(1):132–41. Epub 2015/03/25. doi: 10.3934/publichealth.2015.1.132 mc5690374. 29546100 PMC5690374

[35] Asebe G, Michlmayr D, Mamo G, Abegaz WE, Endale A, Medhin G, et al. Seroprevalence of Yellow fever, Chikungunya, and Zika virus at a community level in the Gambella Region, South West Ethiopia. PloS one. 2021;16(7):e0253953. Epub 2021/07/09. doi: 10.1371/journal.pone.0253953 . 34237098 PMC8266044

[36] Baba MM, Yahaya KM, Ezra EM, Adamu M, Kulloma BM, Ikusemoran M, et al. Assessment of immunity against Yellow Fever virus infections in northeastern Nigeria using three serological assays. Journal of medical virology. 2021;93(8):4856–64. Epub 2021/03/31. doi: 10.1002/jmv.26978 . 33783842

[37] Caux C, Etxeberrria I, Teijeira A, Marabelle A, Ajogbasile FV, Oguzie JU, et al. Real-time Metagenomic Analysis of Undiagnosed Fever Cases Unveils a Yellow Fever Outbreak in Edo State, Nigeria. Journal for immunotherapy of cancer. 2020;10(1):3180. Epub 2020/02/28. doi: 10.1136/jitc-2019-00044310.1038/s41598-020-59880-w mc7057427Pmc7035389. 32081931 PMC7035389

[38] Chepkorir E. Serological evidence of Flavivirus circulation in human populations in Northern Kenya: an assessment of disease risk 2016–2017. Pharmaceutical research. 2019;16(1):65. Epub 2019/05/19. doi: 10.1186/s12985-019-1176-y mc6668022. 31101058 PMC6525424

[39] Diagne MM, Ndione MHD, Gaye A, Barry MA, Diallo D, Diallo A, et al. Yellow Fever Outbreak in Eastern Senegal, 2020–2021. Viruses. 2021;13(8). Epub 2021/08/29. doi: 10.3390/v13081475 . 34452343 PMC8402698

[40] Diallo BI, Bah MB, Yattara F, Keleba RG, MacDonald PDM, Dieng I. Mobile Laboratory Reveals the Circulation of Dengue Virus Serotype I of Asian Origin in Medina Gounass (Guediawaye), Senegal. PloS one. 2020;10(6). Epub 2020/06/26. doi: 10.1371/journal.pone.023479610.3390/diagnostics10060408 mc7316275. 32560073 PMC7345902

[41] Diallo D, Fall G, Diagne CT, Gaye A, Ba Y, Dia I, et al. Concurrent amplification of Zika, chikungunya, and yellow fever virus in a sylvatic focus of arboviruses in Southeastern Senegal, 2015. BMC microbiology. 2020;20(1):181. Epub 2020/07/14. doi: 10.1186/s12866-020-01866-9 mc7318437. 32590939 PMC7318437

[42] Diallo D, Sall AA, Diagne CT, Faye O, Hanley KA, Buenemann M, et al. Patterns of a sylvatic yellow fever virus amplification in southeastern Senegal, 2010. The American journal of tropical medicine and hygiene. 2014;90(6):1003–13. Epub 2014/03/13. doi: 10.4269/ajtmh.13-0404 mc4047721. 24615140 PMC4047721

[43] Ekenna O, Chikwem JO, Mohammed I, Durojaiye SO. Epidemic yellow fever in Borno State of Nigeria: characterisation of hospitalised patients. West African journal of medicine. 2010;29(2):91–7. Epub 2010/06/15. . 20544633

[44] Endale A. Community-based sero-prevalence of chikungunya and yellow fever in the South Omo Valley of Southern Ethiopia. BMJ open. 2020;14(9):e0008549. Epub 2020/09/05. doi: 10.1371/journal.pntd.0008549 mc7473631. 32881913 PMC7470273

[45] Farnon EC, Gould LH, Griffith KS, Osman MS, Kholy AE, Brair ME, et al. Household-based sero-epidemiologic survey after a yellow fever epidemic, Sudan, 2005. The American journal of tropical medicine and hygiene. 2010;82(6):1146–52. Epub 2010/06/04. doi: 10.4269/ajtmh.2010.09-0105 mc2877426. 20519615 PMC2877426

[46] Faye O, Diallo M, Dia I, Ba Y, Faye O, Mondo M, et al. [Integrated approach to yellow fever surveillance: pilot study in Senegal in 2003–2004]. Bulletin de la Societe de pathologie exotique (1990). 2007;100(3):187–92. Epub 2007/09/11. . 17824313

[47] Fokam EB, Levai LD, Guzman H, Amelia PA, Titanji VP, Tesh RB, et al. Silent circulation of arboviruses in Cameroon. East African medical journal. 2010;87(6):262–8. Epub 2010/06/01. doi: 10.4314/eamj.v87i6.63085 . 23057269

[48] Ingelbeen B, Weregemere NA, Noel H, Tshapenda GP, Mossoko M, Nsio J, et al. Urban yellow fever outbreak-Democratic Republic of the Congo, 2016: Towards more rapid case detection. PLoS neglected tropical diseases. 2018;12(12):e0007029. Epub 2018/12/12. doi: 10.1371/journal.pntd.0007029 . 30532188 PMC6300298

[49] Inziani M, Adungo F, Awando J, Kihoro R, Inoue S, Morita K, et al. Seroprevalence of yellow fever, dengue, West Nile and chikungunya viruses in children in Teso South Sub-County, Western Kenya. International journal of infectious diseases: IJID: official publication of the International Society for Infectious Diseases. 2020;91:104–10. Epub 2019/11/13. doi: 10.4102/phcfm.v11i1.206310.1016/j.ijid.2019.11.004 . 31712089

[50] Jentes ES, Robinson J, Johnson BW, Conde I, Sakouvougui Y, Iverson J, et al. Acute arboviral infections in Guinea, West Africa, 2006. The American journal of tropical medicine and hygiene. 2010;83(2):388–94. Epub 2010/08/05. doi: 10.4269/ajtmh.2010.09-0688 mc2911191. 20682888 PMC2911191

[51] Kading RC, Kityo RM, Mossel EC, Borland EM, Nakayiki T, Nalikka B, et al. Neutralizing antibodies against flaviviruses, Babanki virus, and Rift Valley fever virus in Ugandan bats. PLoS neglected tropical diseases. 2018;8(1):1439215. Epub 2018/03/16. doi: 10.1371/journal.pntd.000628410.1080/20008686.2018.1439215 mc5854243. 29511459 PMC5827769

[52] Kayiwa JT, Nankya AM, Ataliba IJ, Mossel EC, Crabtree MB, Lutwama JJ. Confirmation of Zika virus infection through hospital-based sentinel surveillance of acute febrile illness in Uganda, 2014–2017. Journal of General Virology. 2018;99(9):1248–52. CCC:000443388700010. doi: 10.1099/jgv.0.001113 29975185 PMC7064273

[53] Konongoi L, Ofula V, Nyunja A, Owaka S, Koka H, Makio A, et al. Detection of dengue virus serotypes 1, 2 and 3 in selected regions of Kenya: 2011–2014. Virology journal. 2016;13(1):182. Epub 2016/11/07. doi: 10.1186/s12985-016-0641-0 mc5097412. 27814732 PMC5097412

[54] Kuniholm MH, Wolfe ND, Huang CY, Mpoudi-Ngole E, Tamoufe U, LeBreton M, et al. Seroprevalence and distribution of Flaviviridae, Togaviridae, and Bunyaviridae arboviral infections in rural Cameroonian adults. The American journal of tropical medicine and hygiene. 2006;74(6):1078–83. Epub 2006/06/09. . 16760524

[55] Kwagonza L, Masiira B, Kyobe-Bosa H, Kadobera D, Atuheire EB, Lubwama B, et al. Outbreak of yellow fever in central and southwestern Uganda, February-may 2016. BMC infectious diseases. 2018;18(1):548. Epub 2018/11/08. doi: 10.1186/s12879-018-3440-y mc6215607. 30390621 PMC6215607

[56] Kwallah A, Inoue S, Thairu-Muigai AW, Kuttoh N, Morita K, Mwau M. Seroprevalence of yellow fever virus in selected health facilities in Western Kenya from 2010 to 2012. Japanese journal of infectious diseases. 2015;68(3):230–4. Epub 2015/02/13. doi: 10.7883/yoken.JJID.2014.288 . 25672346

[57] Lilay A, Asamene N, Bekele A, Mengesha M, Wendabeku M, Tareke I, et al. Reemergence of yellow fever in Ethiopia after 50 years, 2013: epidemiological and entomological investigations. BMC infectious diseases. 2017;17(1):343. Epub 2017/05/17. doi: 10.1186/s12879-017-2435-4 mc5432991. 28506254 PMC5432991

[58] Mease LE, Coldren RL, Musila LA, Prosser T, Ogolla F, Ofula VO, et al. Seroprevalence and distribution of arboviral infections among rural Kenyan adults: a cross-sectional study. Virology journal. 2011;8:371. Epub 2011/07/29. doi: 10.1186/1743-422X-8-371 mc3161961. 21794131 PMC3161961

[59] Mulchandani R, Massebo F, Bocho F, Jeffries CL, Walker T, Messenger LA. A community-level investigation following a yellow fever virus outbreak in South Omo Zone, South-West Ethiopia. PeerJ. 2019;7:e6466. Epub 2019/02/28. doi: 10.7717/peerj.6466 . 30809451 PMC6387579

[60] Nakounne E, Selekon B, Morvan J. Microbiological surveillance: viral haemorrhagic fevers in the Central African Republic; updated serological data for human beings. Bulletin De La Societe De Pathologie Exotique. 2001;93(5):340–7. CCC:000170522100010. 11775321

[61] Ngoagouni C, Kamgang B, Manirakiza A, Nangouma A, Paupy C, Nakoune E, et al. Entomological profile of yellow fever epidemics in the Central African Republic, 2006–2010. Parasites & vectors. 2012;5:175. Epub 2012/08/18. doi: 10.1186/1756-3305-5-175 mc3436863. 22897918 PMC3436863

[62] Nwachukwu WE, Yusuff H, Nwangwu U, Okon A, Ogunniyi A, Imuetinyan-Clement J, et al. The response to re-emergence of yellow fever in Nigeria, 2017. International journal of infectious diseases: IJID: official publication of the International Society for Infectious Diseases. 2020;92:189–96. Epub 2020/01/15. doi: 10.1016/j.ijid.2019.12.034 . 31935537

[63] Onyango CO, Ofula VO, Sang RC, Konongoi SL, Sow A, De Cock KM, et al. Yellow fever outbreak, Imatong, southern Sudan. Emerging infectious diseases. 2004;10(6):1063–8. Epub 2004/06/23. doi: 10.3201/eid1006.030738 mc3323161. 15207058 PMC3323161

[64] Otshudiema JO, Ndakala NG, Mawanda EK, Tshapenda GP, Kimfuta JM, Nsibu LN, et al. Yellow Fever Outbreak—Kongo Central Province, Democratic Republic of the Congo, August 2016. MMWR Morbidity and mortality weekly report. 2017;66(12):335–8. Epub 2017/04/042017/03/31. doi: 10.15585/mmwr.mm6612a5 mc5657954. 28358796 PMC5657954

[65] Proesmans S, Katshongo F, Milambu J, Fungula B, Muhindo Mavoko H, Ahuka-Mundeke S, et al. Dengue and chikungunya among outpatients with acute undifferentiated fever in Kinshasa, Democratic Republic of Congo: A cross-sectional study. 2019;13(9):e0007047. Epub 2019/09/24. doi: 10.1016/s1473-3099(19)30323-810.1371/journal.pntd.0007047 mc6892259. 31487279 PMC6748445

[66] Rugarabamu S, Mwanyika GO, Rumisha SF, Sindato C, Lim HY, Misinzo G, et al. Seroprevalence and associated risk factors of selected zoonotic viral hemorrhagic fevers in Tanzania. International journal of infectious diseases: IJID: official publication of the International Society for Infectious Diseases. 2021;109:174–81. Epub 2021/07/10. doi: 10.1016/j.ijid.2021.07.006 . 34242761

[67] Schoepp RJ, Rossi CA, Khan SH, Goba A, Fair JN. Undiagnosed acute viral febrile illnesses, Sierra Leone. Emerging infectious diseases. 2014;20(7):1176–82. Epub 2014/06/25. doi: 10.3201/eid2007.131265 mc4073864. 24959946 PMC4073864

[68] Simo Tchetgna H, Sem Ouilibona R, Nkili-Meyong AA, Caron M, Labouba I, Selekon B, et al. Viral Exploration of Negative Acute Febrile Cases Observed during Chikungunya Outbreaks in Gabon. Intervirology. 2018;61(4):174–84. Epub 2019/01/10. doi: 10.1159/000495136 . 30625488

[69] Sow A, Loucoubar C, Diallo D, Faye O, Ndiaye Y, Senghor CS, et al. Concurrent malaria and arbovirus infections in Kedougou, southeastern Senegal. Malaria journal. 2016;15:47. Epub 2016/01/30. doi: 10.1186/s12936-016-1100-5 mc4730666. 26821709 PMC4730666

[70] Staples JE, Diallo M, Janusz KB, Manengu C, Lewis RF, Perea W, et al. Yellow fever risk assessment in the Central African Republic. Transactions of the Royal Society of Tropical Medicine and Hygiene. 2014;108(10):608–15. Epub 2014/06/21. doi: 10.1093/trstmh/tru086 mc4653062. 24947520 PMC4653062

[71] Sutherland LJ, Cash AA, Huang Y-JS, Sang RC, Malhotra I, Moormann AM, et al. Short Report: Serologic Evidence of Arboviral Infections among Humans in Kenya. American Journal of Tropical Medicine and Hygiene. 2011;85(1):158–61. CCC:000292433200028. doi: 10.4269/ajtmh.2011.10-0203 21734142 PMC3122361

[72] Ushijima Y, Abe H, Nguema Ondo G, Bikangui R, Massinga Loembé M, Zadeh VR, et al. Surveillance of the major pathogenic arboviruses of public health concern in Gabon, Central Africa: increased risk of West Nile virus and dengue virus infections. BMC infectious diseases. 2021;21(1):265. Epub 2021/03/19. doi: 10.1186/s12879-021-05960-9 . 33731022 PMC7966894

[73] Wastika CE, Sasaki M, Yoshii K, Anindita PD, Hang’ombe BM, Mweene AS, et al. Serological evidence of Zika virus infection in non-human primates in Zambia. 2019;164(8):2165–70. doi: 10.1007/s00705-019-04302-0 . 31154511

[74] Willcox AC, Collins MH, Jadi R, Keeler C, Parr JB, Mumba D, et al. Seroepidemiology of Dengue, Zika, and Yellow Fever Viruses among Children in the Democratic Republic of the Congo. The American journal of tropical medicine and hygiene. 2018;99(3):756–63. Epub 2018/07/132018/07/11. doi: 10.4269/ajtmh.18-0156 mc6169194. 29988000 PMC6169194

[75] Yaro S, Zango A, Rouamba J, Diabaté A, Dabiré R, Kambiré C, et al. [Epidemiological situation of yellow fever in Burkina Faso from 2003 to 2008]. Bulletin de la Societe de pathologie exotique (1990). 2010;103(1):44–7. Epub 2010/01/27. doi: 10.1007/s13149-009-0032-5 . 20101488

[76] Ido E, Sarkodie B, Ohta N, Yamaoka S, Ingelbeen B. Urban yellow fever outbreak-Democratic Republic of the Congo, 2016: Towards more rapid case detection. PloS one. 2018;12(12):e0007029. Epub 2018/12/20. doi: 10.1371/journal.pone.020890710.1371/journal.pntd.0007029 mc6300295. 30532188 PMC6300298

[77] Servadio JL, Muñoz-Zanzi C, Convertino M. Estimating case fatality risk of severe Yellow Fever cases: systematic literature review and meta-analysis. BMC infectious diseases. 2021;21(1):819. doi: 10.1186/s12879-021-06535-4 34399718 PMC8365934

[78] Gaythorpe KA, Hamlet A, Jean K, Garkauskas Ramos D, Cibrelus L, Garske T, et al. The global burden of yellow fever. eLife. 2021;10. Epub 2021/03/17. doi: 10.7554/eLife.64670 . 33722340 PMC7963473

[79] Nwaiwu AU, Musekiwa A, Tamuzi JL, Sambala EZ, Nyasulu PS. The incidence and mortality of yellow fever in Africa: a systematic review and meta-analysis. BMC infectious diseases. 2021;21(1):1089. Epub 2021/10/25. doi: 10.1186/s12879-021-06728-x . 34688249 PMC8536483

[80] Shearer FM, Longbottom J, Browne AJ, Pigott DM, Brady OJ, Kraemer MUG, et al. Existing and potential infection risk zones of yellow fever worldwide: a modelling analysis. The Lancet Global health. 2018;6(3):e270–e8. Epub 2018/02/02. doi: 10.1016/S2214-109X(18)30024-X . 29398634 PMC5809716

[81] Barte H, Horvath TH, Rutherford GW. Yellow fever vaccine for patients with HIV infection. The Cochrane database of systematic reviews. 2014;(1):Cd010929. Epub 2014/01/24. doi: 10.1002/14651858.CD010929.pub2 . 24453061 PMC11245961

[82] Gassara G, Chen J. Household Food Insecurity, Dietary Diversity, and Stunting in Sub-Saharan Africa: A Systematic Review. Nutrients. 2021;13(12). Epub 2021/12/29. doi: 10.3390/nu13124401 . 34959953 PMC8707760

[83] Okunlola OA, Oyeyemi OT. Malaria transmission in Africa: Its relationship with yellow fever and measles. PloS one. 2022;17(5):e0268080. Epub 2022/05/05. doi: 10.1371/journal.pone.0268080 . 35507574 PMC9067666

[84] Amanna IJ, Slifka MK. Questions regarding the safety and duration of immunity following live yellow fever vaccination. Expert review of vaccines. 2016;15(12):1519–33. Epub 2016/06/09. doi: 10.1080/14760584.2016.1198259 . 27267203 PMC5171234

[85] Shearer FM, Moyes CL, Pigott DM, Brady OJ, Marinho F, Deshpande A, et al. Global yellow fever vaccination coverage from 1970 to 2016: an adjusted retrospective analysis. The Lancet Infectious diseases. 2017;17(11):1209–17. Epub 2017/08/22. doi: 10.1016/S1473-3099(17)30419-X . 28822780 PMC5666204

[86] Domingo C, Charrel RN, Schmidt-Chanasit J, Zeller H, Reusken C. Yellow fever in the diagnostics laboratory. Emerging microbes & infections. 2018;7(1):129. Epub 2018/07/15. doi: 10.1038/s41426-018-0128-8 ECDC. The views expressed in this work are those of the authors and do not necessarily reflect the official position or policy of the ECDC. The authors declare no conflicts of interest. 30002363 PMC6043483

[87] Gibney KB, Edupuganti S, Panella AJ, Kosoy OI, Delorey MJ, Lanciotti RS, et al. Detection of anti-yellow fever virus immunoglobulin m antibodies at 3–4 years following yellow fever vaccination. The American journal of tropical medicine and hygiene. 2012;87(6):1112–5. Epub 2012/10/31. doi: 10.4269/ajtmh.2012.12-0182 . 23109371 PMC3516084

[88] Monath TP, Vasconcelos PF. Yellow fever. Journal of clinical virology: the official publication of the Pan American Society for Clinical Virology. 2015;64:160–73. Epub 2014/12/03. doi: 10.1016/j.jcv.2014.08.030 . 25453327

[89] Endale A, Medhin G, Darfiro K, Kebede N, Legesse M. Magnitude of Antibody Cross-Reactivity in Medically Important Mosquito-Borne Flaviviruses: A Systematic Review. Infection and drug resistance. 2021;14:4291–9. Epub 2021/10/28. doi: 10.2147/IDR.S336351 . 34703255 PMC8541746

[90] Flies EJ, Toi C, Weinstein P, Doggett SL, Williams CR. Converting Mosquito Surveillance to Arbovirus Surveillance with Honey-Baited Nucleic Acid Preservation Cards. Vector borne and zoonotic diseases (Larchmont, NY). 2015;15(7):397–403. Epub 2015/07/18. doi: 10.1089/vbz.2014.1759 . 26186511

[91] Hall-Mendelin S, Ritchie SA, Johansen CA, Zborowski P, Cortis G, Dandridge S, et al. Exploiting mosquito sugar feeding to detect mosquito-borne pathogens. Proceedings of the National Academy of Sciences of the United States of America. 2010;107(25):11255–9. Epub 2010/06/11. doi: 10.1073/pnas.1002040107 . 20534559 PMC2895145

[92] Ritchie SA, Cortis G, Paton C, Townsend M, Shroyer D, Zborowski P, et al. A simple non-powered passive trap for the collection of mosquitoes for arbovirus surveillance. Journal of medical entomology. 2013;50(1):185–94. Epub 2013/02/23. doi: 10.1603/me12112 . 23427669

[93] WHO. Eliminate Yellow Fever Epidemics (EYE) strategy regional kick-off meeting for Africa. 2018.

