# Supplementary material for: Epidemiology of yellow fever virus in humans, arthropods, and non-human primates in sub-Saharan Africa: A systematic review and meta-analysis
Source: PLoS Negl Trop Dis. 2022 Jul 22;16(7):e0010610. doi: 10.1371/journal.pntd.0010610 (PMC9307179; doi:10.1371/journal.pntd.0010610)
Supplement: S3 Table — (PDF) [file pntd.0010610.s003.pdf]

S3 Table. Items for risk of bias assessment

| **Hoy et al. tool for cross sectional studies** | **Yes (1)/No (0)** |
|---|---|
| **External validity** | |
| 1. Was the study's target population a close representation of the national population in relation to Yellow fever virus prevalence or case fatality rate? | **1** |
| 2. Was the sampling frame a true or close representation of the population? | **1** |
| 3. Was some form of random selection used to select the sample, OR was a census undertaken? | **1** |
| 4. Was the likelihood of non-response bias minimal? | **1** |
| **Internal validity** | |
| 5. Were data collected directly from the subjects (as opposed to a proxy)? | **1** |
| 6. Was an acceptable case definition used in the study? | **1** |
| 7. Was the study viral detection assay shown to have validity and reliability? | **1** |
| 8. Was the same mode type of sample collected for all subjects? | **1** |
| 9. Was the length of the length of the study period > 1 year? | **1** |
| 10. Were the numerator(s) and denominator(s) for the prevalence or case fatality rate of Yellow fever virus appropriate? | **1** |
| Total score | **10** |
| **Interpretation of the risk of bias tool**<br><br>• 7-10: Low risk of bias<br>• 4-6: Moderate risk of bias<br>• 0-3: High risk of bias | |
