# Supplementary material for: Epidemiology of yellow fever virus in humans, arthropods, and non-human primates in sub-Saharan Africa: A systematic review and meta-analysis
Source: PLoS Negl Trop Dis. 2022 Jul 22;16(7):e0010610. doi: 10.1371/journal.pntd.0010610 (PMC9307179; doi:10.1371/journal.pntd.0010610)
Supplement: S4 Table — (PDF) [file pntd.0010610.s004.pdf]

S4 Table. Main reasons of exclusion of eligible studies

N°	Author, Year	Title	Reason of exclusion
1	Abdullahi, 2021	The interplay between environmental factors, vector competence and vaccine immunodynamics as possible explanation of the 2019 yellow fever re-emergence in Nigeria.	Review
2	Abílio, 2020	First confirmed occurrence of the yellow fever virus and dengue virus vector Aedes (Stegomyia) luteocephalus (Newstead, 1907) in Mozambique.	No data on YFV prevalence or case fatality rate
3	Adungo, 2016	Development and Characterization of Monoclonal Antibodies to Yellow Fever Virus and Application in Antigen Detection and IgM Capture Enzyme-Linked Immunosorbent Assay.	No data on YFV prevalence or case fatality rate
4	Agboli, 2021	Mosquito-Associated Viruses and Their Related Mosquitoes in West Africa.	Review
5	Ahmed, 2017	Yellow fever from Angola and Congo: a storm gathers.	Review
6	Ahmed, 2021	First report of epidemic dengue fever and malaria co-infections among internally displaced persons in humanitarian camps of North Darfur, Sudan.	No data on YFV prevalence or case fatality rate
7	Akoua-Koffi, 2001	[Investigation surrounding a fatal case of yellow fever in Côte d'Ivoire in 1999].	Duplicates
8	Alencar, 2021	Natural Infection and Vertical Transmission of Zika Virus in Sylvatic Mosquitoes Aedes albopictus and Haemagogus leucocelaenus from Rio de Janeiro, Brazil.	Study outside Africa
9	Althouse, 2015	Impact of climate and mosquito vector abundance on sylvatic arbovirus circulation dynamics in Senegal.	No data on YFV prevalence or case fatality rate
10	Amraoui, 2019	Potential of Aedes albopictus to cause the emergence of arboviruses in Morocco.	No data on YFV prevalence or case fatality rate
11	Anonymous, 2016	Yellow fever spreads from Angola.	Full text or abstract not found
12	Anonymous, 2018	Yellow fever in Nigeria.	No data on YFV prevalence or case fatality rate
13	Appawu, 2006	Surveillance of viral haemorrhagic fevers in ghana: entomological assessment of the risk of transmission in the northern regions.	No data on YFV prevalence or case fatality rate
14	Attoh-Toure, 2010	Resurgence of yellow fever epidemics in Cote-d'Ivoire.	No data on YFV prevalence or case fatality rate
15	Awando, 2013	Seroprevalence of Anti-Dengue Virus 2 Serocomplex antibodies in out-patients with fever visiting selected hospitals in rural parts of western Kenya in 2010-2011: a cross sectional study	No data on YFV prevalence or case fatality rate
16	Baba, 2013	Evidence of arbovirus co-infection in suspected febrile malaria and typhoid patients in Nigeria.	Case report
17	Bagcchi, 2017	Looking back at yellow fever in Angola.	Comment on an article
18	Bamou, 2021	An update on the mosquito fauna and mosquito-borne diseases distribution in Cameroon.	Review
19	Barennes, 2002	[An epidemic risk of yellow fever in Burkina Faso despite a rapid immunisation riposte: role of a multidisciplinary investigation team].	Case report
20	Beck, 2013	Phylogeographic reconstruction of African yellow fever virus isolates indicates recent simultaneous dispersal into east and west Africa.	No data on YFV prevalence or case fatality rate
21	Beressa, 2021	Potential Benefits of Antiviral African Medicinal Plants in the Management of Viral Infections: Systematic Review.	Review
22	Bisimwa, 2016	Molecular detection of arboviruses in Aedes mosquitoes collected from Kyela district, Tanzania.	No data on YFV prevalence or case fatality rate
23	Boga, 2019	Simultaneous detection of Dengue virus, Chikungunya virus, Zika virus, Yellow fever virus and West Nile virus.	Study outside Africa
24	Brinkmann, 2017	Development and preliminary evaluation of a multiplexed amplification and next generation sequencing method for viral hemorrhagic fever diagnostics.	Study outside Africa

25	Buechler, 2017	Seroprevalence of Zika Virus in Wild African Green Monkeys and Baboons.	No data on YFV prevalence or case fatality rate
26	Canela Soler, 2009	A mortality study of the last outbreak of yellow fever in Barcelona City (Spain) in 1870.	Study outside Africa
27	Çavdaroğlu, 2021	The spread of Yellow fever amidst the COVID-19 pandemic in Africa and the ongoing efforts to mitigate it.	Review
28	Chaubal, 2018	Development of single step RT-PCR for detection of Kyasanur forest disease virus from clinical samples.	No data on YFV prevalence or case fatality rate
29	Chisenga, 2020	Sero-prevalence of arthropod-borne viral infections among Lukanga swamp residents in Zambia.	No data on YFV prevalence or case fatality rate
30	Clara, 2020	Community-Based Surveillance in Cote D'Ivoire.	No data on YFV prevalence or case fatality rate
31	Coelho, 2018	Phylogeny of Yellow Fever Virus, Uganda, 2016.	Duplicates
32	Cooke, 2005	Yellow fever in Guinea and Mali.	Full text or abstract not found
33	Coulibaly, 2000	Prevalence and control of zoonotic diseases: collaboration between public health workers and veterinarians in Burkina Faso.	No data on YFV prevalence or case fatality rate
34	Cracknell Daniels, 2021	Yellow fever in Asia-a risk analysis.	Study outside Africa
35	Dash, 2012	Development of a SYBR green I based RT-PCR assay for yellow fever virus: application in assessment of YFV infection in Aedes aegypti.	No data on YFV prevalence or case fatality rate
36	Diallo, 2013	Bloodfeeding patterns of sylvatic arbovirus vectors in southeastern Senegal.	No data on YFV prevalence or case fatality rate
37	Diallo, 2012	Larval ecology of mosquitoes in sylvatic arbovirus foci in southeastern Senegal.	No data on YFV prevalence or case fatality rate
38	Dimeglio, 2019	Hepatitis E Virus Infections among Patients with Acute Febrile Jaundice in Burkina Faso.	No data on YFV prevalence or case fatality rate
39	Djarma, 2021	[Recrudescence of yellow fever in Chad: case report of the last confirmed case in the health district of Lai-Chad].	Case report
40	Drosten, 2002	Rapid detection and quantification of RNA of Ebola and Marburg viruses, Lassa virus, Crimean-Congo hemorrhagic fever virus, Rift Valley fever virus, dengue virus, and yellow fever virus by real-time reverse transcription-PCR.	Sample size < or = 10 participants
41	Efunshile, 2021	Mosquito control at a tertiary teaching hospital in Nigeria.	No data on YFV prevalence or case fatality rate
42	Escadafal, 2014	Rapid molecular assays for the detection of yellow fever virus in low-resource settings.	Not possible to extract data on YFV prevalence or case fatality rate
43	Gadia, 2017	Identification of pathogens for differential diagnosis of fever with jaundice in the Central African Republic: a retrospective assessment, 2008-2010.	No data on YFV prevalence or case fatality rate
44	Garske, 2014	Yellow Fever in Africa: estimating the burden of disease and impact of mass vaccination from outbreak and serological data.	No data on YFV prevalence or case fatality rate
45	Gaythorpe, 2021	The global burden of yellow fever.	Review
46	Giovanetti, 2019	Yellow Fever Virus Reemergence and Spread in Southeast Brazil, 2016-2019.	Study outside Africa
47	Goenaga , 2012	Isolation of yellow fever virus from mosquitoes in Misiones province, Argentina.	Study outside Africa
48	Goodman, 2021	Technical viability of the YF MAC-HD ELISA kit for use in yellow fever-endemic regions.	Selection of study participants with already Flavivirus result known
49	Green, 2016	Yellow fever continues to spread in Angola.	No data on YFV prevalence or case fatality rate
50	Grobbelaar, 2016	Resurgence of Yellow Fever in Angola, 2015-2016.	Sample size < or = 10 participants
51	Hamer, 2018	Fatal Yellow Fever in Travelers to Brazil, 2018.	Study outside Africa

52	Hamid, 2021	Stegomyia indices of Aedes aquatic stages in El Geneina town, Western Sudan.	No data on YFV prevalence or case fatality rate
53	Hamlet, 2021	Seasonality of agricultural exposure as an important predictor of seasonal yellow fever spillover in Brazil.	Study outside Africa
54	Isa, 2021	Genetic diversity of Dengue virus serotypes circulating among Aedes mosquitoes in selected regions of northeastern Nigeria.	No data on YFV prevalence or case fatality rate
55	Jaenson, 2012	[Yellow fever epidemic threatens refugee camps in western Sudan].	Article not in English or in French
56	Joannides, 2021	Species composition and risk of transmission of some Aedes-borne arboviruses in some sites in Northern Ghana.	No data on YFV prevalence or case fatality rate
57	Johnson, 2021	Laboratory capacity assessments in 25 African countries at high risk of yellow fever, August-December 2018.	Not possible to extract data on YFV prevalence or case fatality rate
58	Jupp, 2002	Laboratory vector competence experiments with yellow fever virus and five South African mosquito species including Aedes aegypti.	No data on YFV prevalence or case fatality rate
59	Kallas, 2019	Predictors of mortality in patients with yellow fever: an observational cohort study.	Article not in English or in French
60	Kamgang, 2019	Potential of Aedes albopictus and Aedes aegypti (Diptera: Culicidae) to transmit yellow fever virus in urban areas in Central Africa.	No data on YFV prevalence or case fatality rate
61	Konan, 2011	Entomological investigations conducted around ten cases of yellow fever in 2009 in the Denguele sanitary region, Cote-d'Ivoire.	No data on YFV prevalence or case fatality rate
62	Konan, 2009	[Entomological investigation following the re-emergence of yellow fever in 2008 in Abidjan area (Côte d'Ivoire)].	No data on YFV prevalence or case fatality rate
63	Konan1, 2014	Management of the yellow fever epidemic in 2010 in Seguela (Cote d'Ivoire): value of multidisciplinary investigation.	No data on YFV prevalence or case fatality rate
64	Kone, 2013	Entomological evaluation of the risk of urban outbreak of yellow fever in 2008 in Abidjan, Cote d'Ivoire.	No data on YFV prevalence or case fatality rate
65	Koraka, 2002	Reactivity of serum samples from patients with a flavivirus infection measured by immunofluorescence assay and ELISA.	Selection of study participants with already Flavivirus result known
66	Kraemer, 2019	Spread of yellow fever virus outbreak in Angola and the Democratic Republic of the Congo 2015-16: a modelling study (vol 17, pg 330, 2017).	No data on YFV prevalence or case fatality rate
67	Kraemer, 2017	Spread of yellow fever virus outbreak in Angola and the Democratic Republic of the Congo 2015-16: a modelling study.	Duplicates
68	Kwallah, 2013	A real-time reverse transcription loop-mediated isothermal amplification assay for the rapid detection of yellow fever virus.	Selection of study participants with already Flavivirus result known
69	LaBeaud, 2011	Arbovirus Prevalence in Mosquitoes, Kenya.	No data on YFV prevalence or case fatality rate
70	Leal, 2020	Abundance and Updated Distribution of Aedes aegypti (Diptera: Culicidae) in Cabo Verde Archipelago: A Neglected Threat to Public Health.	No data on YFV prevalence or case fatality rate
71	Leta, 2018	Global risk mapping for major diseases transmitted by Aedes aegypti and Aedes albopictus.	Selection of study participants with already Flavivirus result known
72	Li, 2019	Rift Valley Fever Virus and Yellow Fever Virus in Urine: A Potential Source of Infection.	Selection of study participants with already Flavivirus result known
73	Li, 2017	Adaptive Diversification Between Yellow Fever Virus West African and South American Lineages: A Genome-Wide Study.	Selection of study participants with already Flavivirus result known
74	Li, 2022	Molecular epidemiology of yellow fever virus in Africa: A perspective of the phylogeographic split between East/Central African and West African lineages.	No data on YFV prevalence or case fatality rate
75	Liu, 2016	Development of a TaqMan Array Card for Acute-Febrile-Illness Outbreak Investigation and Surveillance of Emerging Pathogens, Including Ebola Virus.	Selection of study participants with already Flavivirus result known

76	Liu, 2021	Preparation and application of yellow fever virus NS1 protein-specific monoclonal antibodies.	Study outside Africa
77	Mackenzie, 2022	Pneumococcal conjugate vaccination schedules in infants-acquisition, immunogenicity, and pneumococcal conjugate and yellow fever vaccine co-administration study.	No data on YFV prevalence or case fatality rate
78	Makiala-Mandanda, 2018	Identification of Dengue and Chikungunya Cases Among Suspected Cases of Yellow Fever in the Democratic Republic of the Congo.	Selection of study participants with already Flavivirus result known
79	Makiala-Mandanda, 2017	High Prevalence and Diversity of Hepatitis Viruses in Suspected Cases of Yellow Fever in the Democratic Republic of Congo.	Selection of study participants with already Flavivirus result known
80	Makiala-Mandanda, 2021	Herpes Infections in Suspected Cases of Yellow Fever in the Democratic Republic of the Congo.	No data on YFV prevalence or case fatality rate
81	Markoff, 2013	Yellow fever outbreak in Sudan.	Comment on an article
82	Mayanja, 2021	Mosquito-borne arboviruses in Uganda: history, transmission and burden.	Review
83	Mbanzulu, 2017	Mosquito-borne viruses circulating in Kinshasa, Democratic Republic of the Congo.	Not possible to extract data on YFV prevalence or case fatality rate
84	McArthur, 2020	Molecular Characterization of Hamster-Adapted Yellow Fever Virus.	No data on YFV prevalence or case fatality rate
85	McMullan, 2012	Using next generation sequencing to identify yellow fever virus in Uganda.	Sample size < or = 10 participants
86	Mendez, 2007	Detection of yellow fever virus by reverse transcriptase polymerase chain reaction in wild monkeys: a sensitive tool for epidemiologic surveillance.	Sample size < or = 10 participants
87	Méndez, 2013	Development of a reverse transcription polymerase chain reaction method for yellow fever virus detection.	No data on YFV prevalence or case fatality rate
88	Montoya-Ruiz, 2018	Epidemiological Surveillance of Viral Hemorrhagic Fevers With Emphasis on Clinical Virology.	No data on YFV prevalence or case fatality rate
89	Mukadi Kakoni, 2021	Leptospirosis as a cause of fever associated with jaundice in the Democratic Republic of the Congo.	Not possible to extract data on YFV prevalence or case fatality rate
90	Mullaert, 2015	Diphtheria, tetanus, poliomyelitis, yellow fever and hepatitis B seroprevalence among HIV1-infected migrants. Results from the ANRS VIHVO vaccine sub-study.	Study outside Africa
91	Mushi, 2021	A Longitudinal Study Regarding the Health Profile of the 2017 South African Hajj Pilgrims.	No data on YFV prevalence or case fatality rate
92	Mutebi, 2004	Genetic relationships and evolution of genotypes of yellow fever virus and other members of the yellow fever virus group within the Flavivirus genus based on the 3' noncoding region.	No data on YFV prevalence or case fatality rate
93	Mutebi, 2001	Phylogenetic and evolutionary relationships among yellow fever virus isolates in Africa.	No data on YFV prevalence or case fatality rate
94	Nathan, 2001	Shortage of vaccines during a yellow fever outbreak in Guinea.	Not possible to extract data on YFV prevalence or case fatality rate
95	Ndumu, 2021	Serological evidence of Rift Valley fever virus infection among domestic ruminant herds in Uganda.	No data on YFV prevalence or case fatality rate
96	Niedrig, 2008	Evaluation of an indirect immunofluorescence assay for detection of immunoglobulin M (IgM) and IgG antibodies against yellow fever virus.	Study outside Africa
97	No author listed, 2016	Reining in Angola's yellow fever outbreak.	Review
98	No author listed, 2016	Yellow fever in Angola.	Review
99	Nomhwange, 2021	The resurgence of yellow fever outbreaks in Nigeria: a 2-year review 2017-2019.	Not possible to extract data on YFV prevalence or case fatality rate

100	Nunes, 2011	Evaluation of two molecular methods for the detection of Yellow fever virus genome.	Study outside Africa
101	Nunes, 2015	Analysis of a Reverse Transcription Loop-mediated Isothermal Amplification (RT-LAMP) for yellow fever diagnostic.	Study outside Africa
102	Ochola, 2022	Mosquitoes Larvicidal Activity of Ocimum kilimandscharicum Oil Formulation under Laboratory and Field-Simulated Conditions.	No data on YFV prevalence or case fatality rate
103	Ofosu-Appiah, 2022	An evaluation of the diagnostic performance characteristics of the Yellow Fever IgM immunochromatographic rapid diagnostic test kit from SD Biosensor in Ghana.	Selection of study participants with already Flavivirus result known
104	Oladepo, 2021	Outcome of reminder text messages intervention on completion of routine immunization in rural areas, Nigeria.	No data on YFV prevalence or case fatality rate
105	Olivero, 2016	Dengue Virus Seroconversion in Travelers to Dengue-Endemic Areas.	Study outside Africa
106	Omololu, 2019	YELLOW FEVER CHILDHOOD IMMUNIZATION COVERAGE IN JOS NORTH LOCAL GOVERNMENT AREA, NORTH CENTRAL NIGERIA: 2015 – 2017	No data on YFV prevalence or case fatality rate
107	Onyango, 2004	Yellow fever outbreak, southern Sudan, 2003.	No data on YFV prevalence or case fatality rate
108	Onyango, 2004	Yellow Fever Outbreak; Imatong; Southern Sudan.	Duplicates
109	Phan, 2019	Genomic sequence of yellow fever virus from a Dutch traveller returning from the Gambia-Senegal region, the Netherlands, November 2018.	Study outside Africa
110	Phoutrides, 2011	Dengue virus seroprevalence among febrile patients in Bamako, Mali: results of a 2006 surveillance study.	Selection of study participants with already Flavivirus result known
111	Ramos Junior, 2015	Yellow fever risk assessment in the Central African Republic.	Duplicates
112	Raulino, 2021	Multiplex detection of antibodies to Chikungunya, O'nyong-nyong, Zika, Dengue, West Nile and Usutu viruses in diverse non-human primate species from Cameroon and the Democratic Republic of Congo.	No data on YFV prevalence or case fatality rate
113	Rojas, 2018	Internally Controlled, Multiplex Real-Time Reverse Transcription PCR for Dengue Virus and Yellow Fever Virus Detection.	Study outside Africa
114	Rossetto, 2016	REPORTING DELAY DURING THE YELLOW FEVER OUTBREAK, ANGOLA, 2016.	No data on YFV prevalence or case fatality rate
115	Rubbo, 2018	Evidence of human leptospirosis cases in a cohort of febrile patients in Bangui, Central African Republic: a retrospective study, 2012-2015.	Selection of study participants with already Flavivirus result known
116	Sánchez-Seco, 2006	Detection and subtyping of dengue 1-4 and yellow fever viruses by means of a multiplex RT-nested-PCR using degenerated primers.	Study outside Africa
117	Scheck, 2022	FluoRNT: A robust, efficient assay for the detection of neutralising antibodies against yellow fever virus 17D.	Study outside Africa
118	Selemane, 2019	Epidemiological monitoring of the last outbreak of yellow fever in Brazil An outlook from Portugal.	Study outside Africa
119	Shi, 2016	Surveillance of mosquito-borne infectious diseases in febrile travelers entering China via Shenzhen ports, China, 2013.	Study outside Africa
120	Simões, 2012	Evaluation of accuracy and reliability of the plaque reduction neutralization test (micro-PRNT) in detection of yellow fever virus antibodies.	Study outside Africa
121	Simon-Loriere, 2017	Autochthonous Japanese Encephalitis with Yellow Fever Coinfection in Africa.	Case report
122	Soghaier, 2013	Yellow Fever outbreak in Darfur, Sudan in October 2012; the initial outbreak investigation report.	Sample size < or = 10 participants
123	Sow, 2019	Seroreactivity to Chikungunya and West Nile Viruses in Rwandan Blood Donors.	No data on YFV prevalence or case fatality rate
124	Stock, 2015	Development and characterization of polyclonal peptide antibodies for the detection of Yellow fever virus proteins.	No data on YFV prevalence or case fatality rate
125	Stock, 2013	Biological and phylogenetic characteristics of yellow fever virus lineages from West Africa.	No data on YFV prevalence or case fatality rate

126	Sutherland, 2011	Serologic evidence of arboviral infections among humans in Kenya.	Duplicates
127	Tricou, 2020	Hepatitis E virus outbreak associated with rainfall in the Central African Republic in 2008-2009.	Selection of study participants with already Flavivirus result known
128	Trindade, 2020	Detection of Yellow Fever Virus by Quantitative Real-Time PCR (qPCR).	No data on YFV prevalence or case fatality rate
129	Umoke, 2021	Delay in health-seeking behaviour: Implication to yellow fever outcome in the 2019 outbreak in Nigeria.	Selection of study participants with already Flavivirus result known
130	Vianello, 2019	Ocular Findings in Yellow Fever Infection.	Case report
131	von Lindern, 2006	Genome analysis and phylogenetic relationships between east, central and west African isolates of Yellow fever virus.	No data on YFV prevalence or case fatality rate
132	Wat'senga Tezzo, 2021	High Aedes spp. larval indices in Kinshasa, Democratic Republic of Congo.	No data on YFV prevalence or case fatality rate
133	WHO, 2006	Outbreak news. Yellow fever, Cote d'Ivoire.	No data on YFV prevalence or case fatality rate
134	WHO, 2002	Yellow fever, Senegal--update.	No data on YFV prevalence or case fatality rate
135	WHO, 2002	Yellow fever, Senegal (update).	No data on YFV prevalence or case fatality rate
136	WHO, 2007	Outbreak news. Yellow fever, Togo.	No data on YFV prevalence or case fatality rate
137	WHO, 2007	Outbreak news. Yellow fever, Togo--update.	No data on YFV prevalence or case fatality rate
138	WHO, 2008	Outbreak news. Yellow fever, Liberia.	No data on YFV prevalence or case fatality rate
139	WHO, 2008	Outbreak news. Yellow fever, Guinea.	No data on YFV prevalence or case fatality rate
140	WHO, 2009	Outbreak news. Yellow fever, Republic of the Congo.	No data on YFV prevalence or case fatality rate
141	WHO, 2010	Yellow fever, Senegal.	No data on YFV prevalence or case fatality rate
142	WHO, 2010	Yellow fever surveillance and outbreak response: revision of case definitions, October 2010.	Review
143	WHO, 2011	Outbreak news. Yellow fever, Sierra Leone.	No data on YFV prevalence or case fatality rate
144	WHO, 2011	Outbreak news. Yellow fever, Côte d'Ivoire.	No data on YFV prevalence or case fatality rate
145	WHO, 2011	Outbreak news. Yellow fever, Uganda.	No data on YFV prevalence or case fatality rate
146	WHO, 2011	Outbreak news. Yellow fever, Côte d'Ivoire –update.	No data on YFV prevalence or case fatality rate
147	WHO, 2012	Outbreak news. Yellow fever, Sudan.	No data on YFV prevalence or case fatality rate
148	WHO, 2012	Outbreak news. Yellow fever, Sudan – update.	No data on YFV prevalence or case fatality rate
149	Woodall, 2016	Another pandemic disaster looms: yellow fever spreading from Angola.	Editorial
150	Woodall, 2016	Why is the yellow fever outbreak in Angola a 'threat to the entire world'?	Editorial
151	Yang, 2010	Detection of Chikungunya Virus and Arboviruses in Mosquito Vectors.	No data on YFV prevalence or case fatality rate
152	Yao, 2021	Simultaneous Detection of Ebola Virus and Pathogens Associated With Hemorrhagic Fever by an Oligonucleotide Microarray.	Study outside Africa
153	Yen, 2016	Seroprevalence of antibodies against dengue virus among pregnant women in the Democratic Republic of Sao Tome and Principe.	No data on YFV prevalence or case fatality rate
154	Yuill, 2013	Latest outbreak news from ProMED-mail. Yellow fever outbreak-Darfur Sudan and Chad.	No data on YFV prevalence or case fatality rate

155	Zhang, 2019	Next-generation Sequencing Study of Pathogens in Serum from Patients with Febrile Jaundice in Sierra Leone.	Selection of study participants with already Flavivirus result known
156	Zhao, 2020	Modelling the effective reproduction number of vector-borne diseases: the yellow fever outbreak in Luanda, Angola 2015-2016 as an example.	No data on YFV prevalence or case fatality rate
157	Zhao, 2018	Modelling the large-scale yellow fever outbreak in Luanda, Angola, and the impact of vaccination.	No data on YFV prevalence or case fatality rate

Reference

1. Abdullahi IN, Anka AU, Emeribe AU, Umar K, Adekola HA, Uzairue L, et al. The interplay between environmental factors, vector competence and vaccine immunodynamics as possible explanation of the 2019 yellow fever re-emergence in Nigeria. New microbes and new infections. 2021;41:100858. Epub 2021/04/30. doi: 10.1016/j.nmni.2021.100858. PubMed PMID: 33912348; PubMed Central PMCID: PMCPMC8066781.
2. Abílio AP, Kampango A, Armando EJ, Gudo ES, das Neves LCB, Parreira R, et al. First confirmed occurrence of the yellow fever virus and dengue virus vector Aedes (Stegomyia) luteocephalus (Newstead, 1907) in Mozambique. Parasites & vectors. 2020;13(1):350. Epub 2020/10/07. doi: 10.1186/s13071-020-04217-9. PubMed PMID: 33019944; PubMed Central PMCID: PMCPMC7537105.
3. Adungo F, Yu F, Kamau D, Inoue S, Hayasaka D, Posadas-Herrera G, et al. Development and Characterization of Monoclonal Antibodies to Yellow Fever Virus and Application in Antigen Detection and IgM Capture Enzyme-Linked Immunosorbent Assay. Clinical and vaccine immunology : CVI. 2016;23(8):689-97. Epub 2016/06/17. doi: 10.1128/cvi.00209-16. PubMed PMID: 27307452; PubMed Central PMCID: PMCPmc4979174.
4. Agboli E, Zahouli JBZ, Badolo A, Jöst H. Mosquito-Associated Viruses and Their Related Mosquitoes in West Africa. Viruses. 2021;13(5). Epub 2021/06/03. doi: 10.3390/v13050891. PubMed PMID: 34065928; PubMed Central PMCID: PMCPMC8151702.
5. Ahmed A, Eldigail M, Elduma A, Breima T, Dietrich I, Ali Y, et al. First report of epidemic dengue fever and malaria co-infections among internally displaced persons in humanitarian camps of North Darfur, Sudan. International journal of infectious diseases : IJID : official publication of the International Society for Infectious Diseases. 2021;108:513-6. Epub 2021/05/28. doi: 10.1016/j.ijid.2021.05.052. PubMed PMID: 34044142; PubMed Central PMCID: PMCPMC8860570.
6. Ahmed QA, Memish ZA. Yellow fever from Angola and Congo: a storm gathers. Tropical doctor. 2017;47(2):92-6. Epub 2017/04/21. doi: 10.1177/0049475517699726. PubMed PMID: 28424031.
7. Akoua-Koffi C, Diarrassouba S, Bénié VB, Ngbichi JM, Bozoua T, Bosson A, et al. [Investigation surrounding a fatal case of yellow fever in Côte d'Ivoire in 1999]. Bulletin de la Societe de pathologie exotique (1990). 2001;94(3):227-30. Epub 2001/10/30. PubMed PMID: 11681215.
8. Alencar J, Ferreira de Mello C, Brisola Marcondes C, Érico Guimarães A, Toma HK, Queiroz Bastos A, et al. Natural Infection and Vertical Transmission of Zika Virus in Sylvatic Mosquitoes Aedes albopictus and Haemagogus leucocelaenus from Rio de Janeiro, Brazil. Tropical medicine and infectious disease. 2021;6(2). Epub 2021/07/03. doi: 10.3390/tropicalmed6020099. PubMed PMID: 34207935; PubMed Central PMCID: PMCPMC8293354.

9. Althouse BM, Hanley KA, Diallo M, Sall AA, Ba Y, Faye O, et al. Impact of climate and mosquito vector abundance on sylvatic arbovirus circulation dynamics in Senegal. The American journal of tropical medicine and hygiene. 2015;92(1):88-97. Epub 2014/11/19. doi: 10.4269/ajtmh.13-0617. PubMed PMID: 25404071; PubMed Central PMCID: PMCPmc4347398.
10. Amraoui F, Ben Ayed W, Madec Y, Faraj C, Himmi O, Btissam A, et al. Potential of Aedes albopictus to cause the emergence of arboviruses in Morocco. PLoS neglected tropical diseases. 2019;13(2):6997-. PubMed PMID: CCC:000459970700011.
11. Anonymous. Yellow fever spreads from Angola. Bmj-British Medical Journal. 2016;353:NIL_19-NIL_. PubMed PMID: CCC:000376447700014.
12. Anonymous. Yellow fever in Nigeria. Bulletin of the World Health Organization. 2018;96(1):5-. PubMed PMID: CCC:000425780600007.
13. Appawu M, Dadzie S, Abdul H, Asmah H, Boakye D, Wilson M, et al. Surveillance of viral haemorrhagic fevers in ghana: entomological assessment of the risk of transmission in the northern regions. Ghana medical journal. 2006;40(4):137-41. Epub 2007/05/15. doi: 10.4314/gmj.v40i3.55269. PubMed PMID: 17496987; PubMed Central PMCID: PMCPmc1868007.
14. Attoh-Toure H, Dagnan NS, Tagliante-Saracino J. Resurgence of yellow fever epidemics in Cote-d'Ivoire. Bulletin de la Societe de pathologie exotique (1990). 2010;103(5):323-6. doi: 10.1007/s13149-010-0047-y. PubMed PMID: MEDLINE:20349218.
15. Awando J, Awino, Juliette R, Ongus, Collins O, Matilu M. Seroprevalence of Anti-Dengue Virus 2 Serocomplex Antibodies in out-patients with fever visiting selected hospitals in rural parts of Western Kenya in 2010-2011: a cross sectional study. PAMJ. 2013;16. doi: 10.11604/pamj.2013.16.73.2891.
16. Baba M, Logue CH, Oderinde B, Abdulmaleek H, Williams J, Lewis J, et al. Evidence of arbovirus co-infection in suspected febrile malaria and typhoid patients in Nigeria. Journal of infection in developing countries. 2013;7(1):51-9. Epub 2013/01/18. doi: 10.3855/jidc.2411. PubMed PMID: 23324821.
17. Bagcchi S. Looking back at yellow fever in Angola. The Lancet Infectious diseases. 2017;17(3):269-70. Epub 2017/03/01. doi: 10.1016/s1473-3099(17)30064-6. PubMed PMID: 28244394.
18. Bamou R, Mayi MPA, Djiappi-Tchamen B, Nana-Ndjangwo SM, Nchoutpouen E, Cornel AJ, et al. An update on the mosquito fauna and mosquito-borne diseases distribution in Cameroon. Parasites & vectors. 2021;14(1):527. Epub 2021/10/13. doi: 10.1186/s13071-021-04950-9. PubMed PMID: 34635176; PubMed Central PMCID: PMCPMC8507310.
19. Barennes H, Baldet T, Cassel AM, Kabiré C, Kambou C. [An epidemic risk of yellow fever in Burkina Faso despite a rapid immunisation riposte: role of a multidisciplinary investigation team]. Sante (Montrouge, France). 2002;12(3):323-9. Epub 2002/12/11. PubMed PMID: 12473528.
20. Beck A, Guzman H, Li L, Ellis B, Tesh RB, Barrett AD. Phylogeographic reconstruction of African yellow fever virus isolates indicates recent simultaneous dispersal into east and west Africa. PLoS neglected tropical diseases. 2013;7(3):e1910. Epub 2013/03/22. doi: 10.1371/journal.pntd.0001910. PubMed PMID: 23516640; PubMed Central PMCID: PMCPmc3597480.
21. Beressa TB, Deyno S, Mtewa AG, Aidah N, Tuyiringire N, Lukubye B, et al. Potential Benefits of Antiviral African Medicinal Plants in the Management of Viral Infections: Systematic Review. Frontiers in pharmacology. 2021;12:682794. Epub 2022/01/11. doi:

10.3389/fphar.2021.682794. PubMed PMID: 35002686; PubMed Central PMCID: PMCPMC8740180.
22. Bisimwa NP, Angwenyi S, Kinimi E, Shayo M, Bwihangane BA, Kasanga CJ. Molecular detection of arboviruses in Aedes mosquitoes collected from Kyela district, Tanzania. Revue De Medecine Veterinaire. 2016;167(5-6):138-44. PubMed PMID: CCC:000377772800005.
23. Boga JA, Alvarez-Arguelles ME, Rojo-Alba S, Rodríguez M, de Oña M, Melón S. Simultaneous detection of Dengue virus, Chikungunya virus, Zika virus, Yellow fever virus and West Nile virus. Journal of virological methods. 2019;268:53-5. Epub 2019/04/03. doi: 10.1093/jtm/taz024

10.1016/j.jviromet.2019.03.014. PubMed PMID: 30930286.
24. Brinkmann A, Ergünay K, Radonić A, Kocak Tufan Z, Domingo C, Nitsche A. Development and preliminary evaluation of a multiplexed amplification and next generation sequencing method for viral hemorrhagic fever diagnostics. 2017;11(11):e0006075. doi: 10.1371/journal.pntd.0006075. PubMed PMID: 29155823.
25. Buechler CR, Bailey AL, Weiler AM, Barry GL, Breitbach ME, Stewart LM, et al. Seroprevalence of Zika Virus in Wild African Green Monkeys and Baboons. mSphere. 2017;2(2). Epub 2017/03/16. doi: 10.1128/mSphere.00392-16. PubMed PMID: 28289727; PubMed Central PMCID: PMCPmc5343173.
26. Canela Soler J, Pallarés Fusté MR, Abós Herràndiz R, Nebot Adell C, Lawrence RS. A mortality study of the last outbreak of yellow fever in Barcelona City (Spain) in 1870. Gaceta sanitaria. 2009;23(4):295-9. Epub 2009/03/10. doi: 10.1016/j.gaceta.2008.09.008. PubMed PMID: 19268397.
27. Çavdaroğlu S, Hasan MM, Mohan A, Xenophontos E, Costa A, Aborode AT, et al. The spread of Yellow fever amidst the COVID-19 pandemic in Africa and the ongoing efforts to mitigate it. Journal of medical virology. 2021;93(9):5223-5. Epub 2021/04/20. doi: 10.1002/jmv.27027. PubMed PMID: 33871091; PubMed Central PMCID: PMCPMC8251444.
28. Chaubal G, Sarkale P, Kore P, Yadav P. Development of single step RT-PCR for detection of Kyasanur forest disease virus from clinical samples. Heliyon. 2018;4(2):e00549. Epub 2018/03/22. doi: 10.1016/j.heliyon.2018.e00549. PubMed PMID: 29560461; PubMed Central PMCID: PMCPmc5857640.
29. Chisenga CC, Bosomprah S, Musukuma K, Mubanga C, Chilyabanyama ON, Velu RM, et al. Sero-prevalence of arthropod-borne viral infections among Lukanga swamp residents in Zambia. PloS one. 2020;15(7):e0235322-e. doi: 10.1371/journal.pone.0235322. PubMed PMID: MEDLINE:32609784.
30. Clara A, Ndiaye SM, Joseph B, Nzogu MA, Coulibaly D, Alroy KA, et al. Community-Based Surveillance in Cote D'Ivoire. Health security. 2020;18:S23-S33. PubMed PMID: CCC:000510329600005.
31. Coelho G, Wilder-Smith A, Burattini MN, Hughes HR, Kayiwa J, Mossel EC, et al. Phylogeny of Yellow Fever Virus, Uganda, 2016. Epidemiology and infection. 2018;24(8):1598-9. Epub 2018/05/31. doi: 10.1017/s0950268818001334

10.3201/eid2408.180588. PubMed PMID: 29798746; PubMed Central PMCID: PMCPmc6056105.
32. Cooke FJ, Shapiro DS. Yellow fever in Guinea and Mali. International Journal of Infectious Diseases. 2005;9(2):67-. PubMed PMID: CCC:000227764600001.

33. Coulibaly ND, Yameogo KR. Prevalence and control of zoonotic diseases: collaboration between public health workers and veterinarians in Burkina Faso. Acta tropica. 2000;76(1):53-7. Epub 2000/07/29. doi: 10.1016/s0001-706x(00)00090-5. PubMed PMID: 10913767.
34. Cracknell Daniels B, Gaythorpe K, Imai N, Dorigatti I. Yellow fever in Asia-a risk analysis. Journal of travel medicine. 2021;28(3). Epub 2021/01/29. doi: 10.1093/jtm/taab015. PubMed PMID: 33506250; PubMed Central PMCID: PMCPMC8045179.
35. Dash PK, Boutonnier A, Prina E, Sharma S, Reiter P. Development of a SYBR green I based RT-PCR assay for yellow fever virus: application in assessment of YFV infection in Aedes aegypti. Virology journal. 2012;9:27. Epub 2012/01/24. doi: 10.1186/1743-422x-9-27. PubMed PMID: 22264275; PubMed Central PMCID: PMCPmc3296605.
36. Diallo D, Chen R, Diagne CT, Ba Y, Dia I, Sall AA, et al. Bloodfeeding patterns of sylvatic arbovirus vectors in southeastern Senegal. Transactions of the Royal Society of Tropical Medicine and Hygiene. 2013;107(3):200-3. Epub 2013/02/21. doi: 10.1093/trstmh/trs095. PubMed PMID: 23423342; PubMed Central PMCID: PMCPmc3913185.
37. Diallo D, Diagne CT, Hanley KA, Sall AA, Buenemann M, Ba Y, et al. Larval ecology of mosquitoes in sylvatic arbovirus foci in southeastern Senegal. Parasites & vectors. 2012;5:286. Epub 2012/12/12. doi: 10.1186/1756-3305-5-286. PubMed PMID: 23216815; PubMed Central PMCID: PMCPmc3543325.
38. Dimeglio C, Kania D, Mantono JM, Kagoné T, Zida S, Tassembedo S, et al. Hepatitis E Virus Infections among Patients with Acute Febrile Jaundice in Burkina Faso. 2019;11(6). Epub 2019/06/21. doi: 10.3967/bes2019.048

10.3390/v11060554. PubMed PMID: 31207982.
39. Djarma OM, Elisee D, Bolti MA, Sougoudi DA, Diop AB, Haggar FA, et al. [Recrudescence of yellow fever in Chad: case report of the last confirmed case in the health district of Lai-Chad]. The Pan African medical journal. 2021;38:248. Epub 2021/06/10. doi: 10.11604/pamj.2021.38.248.27123. PubMed PMID: 34104296; PubMed Central PMCID: PMCPMC8164432.
40. Drosten C, Göttig S, Schilling S, Asper M, Panning M, Schmitz H, et al. Rapid detection and quantification of RNA of Ebola and Marburg viruses, Lassa virus, Crimean-Congo hemorrhagic fever virus, Rift Valley fever virus, dengue virus, and yellow fever virus by real-time reverse transcription-PCR. Journal of clinical microbiology. 2002;40(7):2323-30. Epub 2002/06/29. doi: 10.1128/jcm.40.7.2323-2330.2002. PubMed PMID: 12089242; PubMed Central PMCID: PMCPmc120575.
41. Efunshile AM, Ojide CK, Igwe D, Onyia B, Jokelainen P, Robertson LJ. Mosquito control at a tertiary teaching hospital in Nigeria. Infection prevention in practice. 2021;3(4):100172. Epub 2021/10/05. doi: 10.1016/j.infpip.2021.100172. PubMed PMID: 34604733; PubMed Central PMCID: PMCPMC8473772.
42. Escadafal C, Faye O, Sall AA, Faye O, Weidmann M, Strohmeier O, et al. Rapid molecular assays for the detection of yellow fever virus in low-resource settings. PLoS neglected tropical diseases. 2014;8(3):e2730. Epub 2014/03/08. doi: 10.1371/journal.pntd.0002730. PubMed PMID: 24603874; PubMed Central PMCID: PMCPMC3945292 Michael Eberhard are employees of the commercial company QIAGEN Lake Constance GmbH. The affiliations to this company do not alter our adherence to all PLOS NTDs policies on sharing data and materials. The authors have declared that no other competing interests exist.
43. Gadia CLB, Manirakiza A. Identification of pathogens for differential diagnosis of fever with jaundice in the Central African Republic: a retrospective assessment, 2008-2010. 2017;17(1):735. doi: 10.1186/s12879-017-2840-8. PubMed PMID: 29187150.

44. Garske T, Van Kerkhove MD, Yactayo S, Ronveaux O, Lewis RF, Staples JE, et al. Yellow Fever in Africa: estimating the burden of disease and impact of mass vaccination from outbreak and serological data. PLoS medicine. 2014;11(5):e1001638. Epub 2014/05/08. doi: 10.1371/journal.pmed.1001638. PubMed PMID: 24800812; PubMed Central PMCID: PMCPmc4011853.
45. Gaythorpe KA, Hamlet A, Jean K, Garkauskas Ramos D, Cibrelus L, Garske T, et al. The global burden of yellow fever. eLife. 2021;10. Epub 2021/03/17. doi: 10.7554/eLife.64670. PubMed PMID: 33722340; PubMed Central PMCID: PMCPMC7963473.
46. Giovanetti M. Yellow Fever Virus Reemergence and Spread in Southeast Brazil, 2016-2019. PLoS neglected tropical diseases. 2019;94(1). Epub 2019/10/11. doi: 10.1371/journal.pntd.0007615

10.1128/jvi.01623-19. PubMed PMID: 31597773; PubMed Central PMCID: PMCPmc6786541.
47. Goenaga S, Fabbri C, Dueñas JC, Gardenal CN, Rossi GC, Calderon G, et al. Isolation of yellow fever virus from mosquitoes in Misiones province, Argentina. Vector borne and zoonotic diseases (Larchmont, NY). 2012;12(11):986-93. Epub 2012/10/03. doi: 10.1089/vbz.2011.0730. PubMed PMID: 23025694.
48. Goodman CH, Demanou M, Mulders M, Mendez-Rico J, Basile AJ. Technical viability of the YF MAC-HD ELISA kit for use in yellow fever-endemic regions. PLoS neglected tropical diseases. 2021;15(6):e0009417. Epub 2021/06/05. doi: 10.1371/journal.pntd.0009417. PubMed PMID: 34086676; PubMed Central PMCID: PMCPMC8177417.
49. Green A. Yellow fever continues to spread in Angola. Lancet (London, England). 2016;387(10037):2493. Epub 2016/06/30. doi: 10.1016/s0140-6736(16)30835-2. PubMed PMID: 27353673.
50. Grobbelaar AA, Weyer J, Moolla N, Jansen van Vuren P, Moises F, Paweska JT. Resurgence of Yellow Fever in Angola, 2015-2016. Emerging infectious diseases. 2016;22(10):1854-5. Epub 2016/08/19. doi: 10.3201/eid2210.160818. PubMed PMID: 27536787; PubMed Central PMCID: PMCPmc5038398.
51. Hamer DH, Angelo K, Caumes E, van Genderen PJJ, Florescu SA, Popescu CP, et al. Fatal Yellow Fever in Travelers to Brazil, 2018. MMWR Morbidity and mortality weekly report. 2018;67(11):340-1. Epub 2018/03/23. doi: 10.15585/mmwr.mm6711e1. PubMed PMID: 29565840; PubMed Central PMCID: PMCPmc5868208.
52. Hamid H, Musa H, Ahmed A, Abdul Azeez T, Adam A, Abdel Malik M, et al. Stegomyia indices of Aedes aquatic stages in El Geneina town, Western Sudan. East Mediterr Health J. 2021;27(12):1189-96. Epub 2022/02/10. doi: 10.26719/emhj.21.073. PubMed PMID: 35137387.
53. Hamlet A, Ramos DG, Gaythorpe KAM, Romano APM, Garske T, Ferguson NM. Seasonality of agricultural exposure as an important predictor of seasonal yellow fever spillover in Brazil. Nature communications. 2021;12(1):3647. Epub 2021/06/17. doi: 10.1038/s41467-021-23926-y. PubMed PMID: 34131128; PubMed Central PMCID: PMCPMC8206143.
54. Isa I, Ndams IS, Aminu M, Chechet G, Dotzauer A, Simon AY. Genetic diversity of Dengue virus serotypes circulating among Aedes mosquitoes in selected regions of northeastern Nigeria. One health (Amsterdam, Netherlands). 2021;13:100348. Epub 2021/11/27. doi: 10.1016/j.onehlt.2021.100348. PubMed PMID: 34825044; PubMed Central PMCID: PMCPMC8605110.
55. Jaenson T. [Yellow fever epidemic threatens refugee camps in western Sudan]. Lakartidningen. 2012;109(48):2231-2. Epub 2013/01/22. PubMed PMID: 23330533.

56. Joannides J, Dzodzomenyo M, Azerigyik F, Agbosu EE, Pratt D, Nyarko Osei JH, et al. Species composition and risk of transmission of some Aedes-borne arboviruses in some sites in Northern Ghana. PloS one. 2021;16(6):e0234675. Epub 2021/06/02. doi: 10.1371/journal.pone.0234675. PubMed PMID: 34061882; PubMed Central PMCID: PMCPMC8168856.
57. Johnson BW, Demanou M, Fall G, Betoulle JL, Obiekea C, Basile AJ, et al. Laboratory capacity assessments in 25 African countries at high risk of yellow fever, August-December 2018. The Pan African medical journal. 2021;38:402. Epub 2021/08/13. doi: 10.11604/pamj.2021.38.402.28886. PubMed PMID: 34381546; PubMed Central PMCID: PMCPMC8325472.
58. Jupp PG, Kemp A. Laboratory vector competence experiments with yellow fever virus and five South African mosquito species including Aedes aegypti. Transactions of the Royal Society of Tropical Medicine and Hygiene. 2002;96(5):493-8. Epub 2002/12/12. doi: 10.1016/s0035-9203(02)90417-7. PubMed PMID: 12474475.
59. Kallas EG, D'Elia Zanella LGFAB, Moreira CHV, Buccheri R, Diniz GBF, Castineiras ACP, et al. Predictors of mortality in patients with yellow fever: an observational cohort study. Lancet Infectious Diseases. 2019;19(7):750-8. PubMed PMID: CCC:000472818900035.
60. Kamgang B, Vazeille M, Yougang AP, Tedjou AN, Wilson-Bahun TA, Mousson L, et al. Potential of Aedes albopictus and Aedes aegypti (Diptera: Culicidae) to transmit yellow fever virus in urban areas in Central Africa. 2019;8(1):1636-41. Epub 2019/11/13. doi: 10.1016/j.ijid.2019.11.004

10.1080/22221751.2019.1688097. PubMed PMID: 31711378.
61. Konan YL, Coulibaly ZI, Allali KB, Tetchi SM, Kone AB, Coulibaly D, et al. Management of the yellow fever epidemic in 2010 in Seguela (Cote d'Ivoire): value of multidisciplinary investigation. Sante publique (Vandoeuvre-les-Nancy, France). 2014;26(6):859-67. doi: 10.3917/spub.146.0859. PubMed PMID: MEDLINE:25629680.
62. Konan YL, Fofana D, Coulibaly ZI, Diallo A, Kone AB, Doannio JMC, et al. Entomological investigations conducted around ten cases of yellow fever in 2009 in the Denguele sanitary region, Cote-d'Ivoire. Bulletin de la Societe de pathologie exotique (1990). 2011;104(4):296-302. doi: 10.1007/s13149-010-0128-y. PubMed PMID: MEDLINE:21293956.
63. Konan YL, Koné AB, Ekra KD, Doannio JM, Odéhouri KP. [Entomological investigation following the re-emergence of yellow fever in 2008 in Abidjan area (Côte d'Ivoire)]. Parasite (Paris, France). 2009;16(2):149-52. Epub 2009/07/10. doi: 10.1051/parasite/2009162149. PubMed PMID: 19585894.
64. Kone AB, Konan YL, Coulibaly ZI, Fofana D, Guindo-Coulibaly N, Diallo M, et al. Entomological evaluation of the risk of urban outbreak of yellow fever in 2008 in Abidjan, Cote d'Ivoire. Medecine et sante tropicales. 2013;23(1):66-71. doi: 10.1684/mst.2013.0153. PubMed PMID: MEDLINE:23693032.
65. Koraka P, Zeller H, Niedrig M, Osterhaus A, Groen J. Reactivity of serum samples from patients with a flavivirus infection measured by immunofluorescence assay and ELISA. Microbes and infection. 2002;4(12):1209-15. doi: 10.1016/s1286-4579(02)01647-7. PubMed PMID: CCC:000179410300002.
66. Kraemer MUG, Faria NR, Reiner RC, Jr. Spread of yellow fever virus outbreak in Angola and the Democratic Republic of the Congo 2015-16: a modelling study (vol 17, pg 330, 2017). Lancet Infectious Diseases. 2019;19(4):E109-E. PubMed PMID: CCC:000462599400001.
67. Kraemer MUG, Faria NR, Reiner RC, Jr., Golding N, Nikolay B, Stasse S, et al. Spread of yellow fever virus outbreak in Angola and the Democratic Republic of the Congo 2015-16: a

modelling study. The Lancet Infectious diseases. 2017;17(3):330-8. Epub 2016/12/27. doi: 10.1016/s1473-3099(16)30513-8. PubMed PMID: 28017559; PubMed Central PMCID: PMCPmc5332542.
68. Kwallah A, Inoue S, Muigai AW, Kubo T, Sang R, Morita K, et al. A real-time reverse transcription loop-mediated isothermal amplification assay for the rapid detection of yellow fever virus. Journal of virological methods. 2013;193(1):23-7. Epub 2013/05/23. doi: 10.1016/j.jviromet.2013.05.004. PubMed PMID: 23692685.
69. LaBeaud AD, Sutherland LJ, Muiruri S, Muchiri EM, Gray LR, Zimmerman PA, et al. Arbovirus Prevalence in Mosquitoes, Kenya. Emerging infectious diseases. 2011;17(2):233-41. doi: 10.3201/eid1702.091666. PubMed PMID: CCC:000287436400011.
70. Leal SDV, Fernandes Varela IB, Lopes Gonçalves AAL, Sousa Monteiro DD, Ramos de Sousa CM, Lima Mendonça MDL, et al. Abundance and Updated Distribution of Aedes aegypti (Diptera: Culicidae) in Cabo Verde Archipelago: A Neglected Threat to Public Health. International journal of environmental research and public health. 2020;17(4). Epub 2020/02/23. doi: 10.1038/s41598-020-59880-w

10.3390/ijerph17041291. PubMed PMID: 32079356; PubMed Central PMCID: PMCPmc7068338.
71. Leta S, Beyene TJ, De Clercq EM, Amenu K, Kraemer MUG, Revie CW. Global risk mapping for major diseases transmitted by Aedes aegypti and Aedes albopictus. International journal of infectious diseases : IJID : official publication of the International Society for Infectious Diseases. 2018;67:25-35. Epub 2017/12/03. doi: 10.1016/j.ijid.2017.11.026. PubMed PMID: 29196275; PubMed Central PMCID: PMCPmc5976855.
72. Li M, Wang B, Li L, Wong G, Liu Y, Ma J, et al. Rift Valley Fever Virus and Yellow Fever Virus in Urine: A Potential Source of Infection. Virologica Sinica. 2019;34(3):342-5. doi: 10.1007/s12250-019-00096-2. PubMed PMID: MEDLINE:30888606.
73. Li Y. Molecular epidemiology of yellow fever virus in Africa: A perspective of the phylogeographic split between East/Central African and West African lineages. Acta tropica. 2022;225:106199. Epub 2021/11/07. doi: 10.1016/j.actatropica.2021.106199. PubMed PMID: 34740635.
74. Li Y, Yang Z. Adaptive Diversification Between Yellow Fever Virus West African and South American Lineages: A Genome-Wide Study. The American journal of tropical medicine and hygiene. 2017;96(3):727-34. Epub 2017/01/04. doi: 10.4269/ajtmh.16-0698. PubMed PMID: 28044043; PubMed Central PMCID: PMCPmc5361553.
75. Liu D, Chen D, Zhang T, Yu N, Ren R, Chen Y, et al. Preparation and application of yellow fever virus NS1 protein-specific monoclonal antibodies. Journal of medical virology. 2020. Epub 2020/09/03. doi: 10.1002/jmv.26455. PubMed PMID: 32841419.
76. Liu J, Ochieng C, Wiersma S, Ströher U, Towner JS, Whitmer S, et al. Development of a TaqMan Array Card for Acute-Febrile-Illness Outbreak Investigation and Surveillance of Emerging Pathogens, Including Ebola Virus. Journal of clinical microbiology. 2016;54(1):49-58. Epub 2015/10/23. doi: 10.1128/jcm.02257-15. PubMed PMID: 26491176; PubMed Central PMCID: PMCPmc4702733.
77. Mackenzie GA, Osei I, Salaudeen R, Secka O, D'Alessandro U, Clarke E, et al. Pneumococcal conjugate vaccination schedules in infants-acquisition, immunogenicity, and pneumococcal conjugate and yellow fever vaccine co-administration study. Trials. 2022;23(1):39. Epub 2022/01/17. doi: 10.1186/s13063-021-05949-4. PubMed PMID: 35033180; PubMed Central PMCID: PMCPMC8760872.

78. Makiala-Mandanda S, Abbate JL, Pukuta-Simbu E, Ahuka-Mundeke S, Muyembe-Tamfum JJ, Leroy EM, et al. Herpes Infections in Suspected Cases of Yellow Fever in the Democratic Republic of the Congo. Medicina (Kaunas, Lithuania). 2021;57(9). Epub 2021/09/29. doi: 10.3390/medicina57090871. PubMed PMID: 34577794; PubMed Central PMCID: PMCPMC8468251.
79. Makiala-Mandanda S, Ahuka-Mundeke S, Abbate JL, Pukuta-Simbu E, Nsio-Mbeta J, Berthet N, et al. Identification of Dengue and Chikungunya Cases Among Suspected Cases of Yellow Fever in the Democratic Republic of the Congo. Vector borne and zoonotic diseases (Larchmont, NY). 2018;18(7):364-70. Epub 2018/05/19. doi: 10.1016/j.vaccine.2018.04.072

10.1089/vbz.2017.2176. PubMed PMID: 29768102.
80. Makiala-Mandanda S, Le Gal F, Ngwaka-Matsung N, Ahuka-Mundeke S, Onanga R, Bivigou-Mboumba B. High Prevalence and Diversity of Hepatitis Viruses in Suspected Cases of Yellow Fever in the Democratic Republic of Congo. 2017;55(5):1299-312. doi: 10.1128/jcm.01847-16. PubMed PMID: 28202798.
81. Markoff L. Yellow fever outbreak in Sudan. The New England journal of medicine. 2013;368(8):689-91. Epub 2013/02/08. doi: 10.1056/NEJMp1300772. PubMed PMID: 23387798.
82. Mayanja MN, Mwiine FN, Lutwama JJ, Ssekagiri A, Egesa M, Thomson EC, et al. Mosquito-borne arboviruses in Uganda: history, transmission and burden. The Journal of general virology. 2021;102(6). Epub 2021/06/25. doi: 10.1099/jgv.0.001615. PubMed PMID: 34166178.
83. Mbanzulu KM, Wumba R, Mukendi J-PK, Zanga JK, Shija F, Bobanga TL, et al. Mosquito-borne viruses circulating in Kinshasa, Democratic Republic of the Congo. International Journal of Infectious Diseases. 2017;57:32-7. PubMed PMID: CCC:000397947500006.
84. McArthur MA, Zhang SL, Li L, Tesh RB, Barrett ADT. Molecular Characterization of Hamster-Adapted Yellow Fever Virus. Vector borne and zoonotic diseases (Larchmont, NY). 2020;20(3):222-7. Epub 2019/12/06. doi: 10.1007/s00103-019-03064-z

10.1089/vbz.2019.2495. PubMed PMID: 31794691; PubMed Central PMCID: PMCPmc7081246.
85. McMullan LK, Frace M, Sammons SA, Shoemaker T, Balinandi S, Wamala JF, et al. Using next generation sequencing to identify yellow fever virus in Uganda. Virology. 2012;422(1):1-5. Epub 2011/10/04. doi: 10.1016/j.virol.2011.08.024. PubMed PMID: 21962764.
86. Mendez JA, Parra E, Neira M, Rey GJ. Detection of yellow fever virus by reverse transcriptase polymerase chain reaction in wild monkeys: a sensitive tool for epidemiologic surveillance. Biomedica : revista del Instituto Nacional de Salud. 2007;27(3):461-7. doi: 10.7705/biomedica.v27i3.209. PubMed PMID: MEDLINE:18320112.
87. Méndez MC, Domingo C, Tenorio A, Pardo LC, Rey GJ, Méndez JA. Development of a reverse transcription polymerase chain reaction method for yellow fever virus detection. Biomedica : revista del Instituto Nacional de Salud. 2013;33 Suppl 1:190-6. Epub 2014/03/22. PubMed PMID: 24652263.
88. Montoya-Ruiz C, Rodas JD. Epidemiological Surveillance of Viral Hemorrhagic Fevers With Emphasis on Clinical Virology. Methods in molecular biology (Clifton, NJ). 2018;1604:55-78. doi: 10.1007/978-1-4939-6981-4_4. PubMed PMID: MEDLINE:28986825.
89. Mukadi Kakoni P, Munyeku Bazitama Y, Nepomuceno JR, Pukuta-Simbu E, Kawhata Mawika F, Kashitu Mujinga G, et al. Leptospirosis as a cause of fever associated with jaundice in the Democratic Republic of the Congo. PLoS neglected tropical diseases.

2021;15(8):e0009670. Epub 2021/08/18. doi: 10.1371/journal.pntd.0009670. PubMed PMID: 34403427; PubMed Central PMCID: PMCPMC8396788.
90. Mullaert J, Abgrall S, Lele N, Batteux F, Slama LB, Meritet JF, et al. Diphtheria, tetanus, poliomyelitis, yellow fever and hepatitis B seroprevalence among HIV1-infected migrants. Results from the ANRS VIHVO vaccine sub-study. Vaccine. 2015;33(38):4938-44. Epub 2015/07/26. doi: 10.1016/j.vaccine.2015.07.036. PubMed PMID: 26209841.
91. Mushi A, Yassin Y, Khan A, Alotaibi B, Parker S, Mahomed O, et al. A Longitudinal Study Regarding the Health Profile of the 2017 South African Hajj Pilgrims. International journal of environmental research and public health. 2021;18(7). Epub 2021/04/04. doi: 10.3390/ijerph18073607. PubMed PMID: 33807142; PubMed Central PMCID: PMCPMC8036399.
92. Mutebi JP, Rijnbrand RC, Wang H, Ryman KD, Wang E, Fulop LD, et al. Genetic relationships and evolution of genotypes of yellow fever virus and other members of the yellow fever virus group within the Flavivirus genus based on the 3' noncoding region. Journal of virology. 2004;78(18):9652-65. Epub 2004/08/28. doi: 10.1128/jvi.78.18.9652-9665.2004. PubMed PMID: 15331698; PubMed Central PMCID: PMCPmc515011.
93. Mutebi JP, Wang H, Li L, Bryant JE, Barrett AD. Phylogenetic and evolutionary relationships among yellow fever virus isolates in Africa. Journal of virology. 2001;75(15):6999-7008. Epub 2001/07/04. doi: 10.1128/jvi.75.15.6999-7008.2001. PubMed PMID: 11435580; PubMed Central PMCID: PMCPmc114428.
94. Nathan N, Barry M, Van Herp M, Zeller H. Shortage of vaccines during a yellow fever outbreak in Guinea. Lancet (London, England). 2001;358(9299):2129-30. Epub 2002/01/11. doi: 10.1016/s0140-6736(01)07185-9. PubMed PMID: 11784630.
95. Ndumu DB, Bakamutumaho B, Miller E, Nakayima J, Downing R, Balinandi S, et al. Serological evidence of Rift Valley fever virus infection among domestic ruminant herds in Uganda. BMC veterinary research. 2021;17(1):157. Epub 2021/04/15. doi: 10.1186/s12917-021-02867-0. PubMed PMID: 33849526; PubMed Central PMCID: PMCPMC8045185.
96. Niedrig M, Kuersteiner O, Herzog C, Sonnenberg K. Evaluation of an indirect immunofluorescence assay for detection of immunoglobulin M (IgM) and IgG antibodies against yellow fever virus. Clinical and Vaccine Immunology. 2008;15(2):177-81. doi: 10.1128/cvi.00078-07. PubMed PMID: CCC:000258666600001.
97. No author listed. Reining in Angola's yellow fever outbreak. Bulletin of the World Health Organization. 2016;94(10):716-7. Epub 2016/11/16. doi: 10.2471/blt.16.031016. PubMed PMID: 27843160; PubMed Central PMCID: PMCPmc5043210.
98. No author listed. Yellow fever in Angola. Bulletin of the World Health Organization. 2016;94(5):314-. PubMed PMID: CCC:000376472800006.
99. Nomhwange T, Jean Baptiste AE, Ezebilo O, Oteri J, Olajide L, Emelife K, et al. The resurgence of yellow fever outbreaks in Nigeria: a 2-year review 2017-2019. BMC infectious diseases. 2021;21(1):1054. Epub 2021/10/13. doi: 10.1186/s12879-021-06727-y. PubMed PMID: 34635069; PubMed Central PMCID: PMCPMC8504075.
100. Nunes MR, Palacios G, Nunes KN, Casseb SM, Martins LC, Quaresma JA, et al. Evaluation of two molecular methods for the detection of Yellow fever virus genome. Journal of virological methods. 2011;174(1-2):29-34. Epub 2011/03/23. doi: 10.1016/j.jviromet.2011.02.025. PubMed PMID: 21419803; PubMed Central PMCID: PMCPmc4109279.
101. Nunes MR, Vianez JL, Jr., Nunes KN, da Silva SP, Lima CP, Guzman H, et al. Analysis of a Reverse Transcription Loop-mediated Isothermal Amplification (RT-LAMP) for yellow fever

diagnostic. Journal of virological methods. 2015;226:40-51. Epub 2015/10/16. doi: 10.1016/j.jviromet.2015.10.003. PubMed PMID: 26459206.
102. Ochola JB, Mutero CM, Marubu RM, Haller BF, Hassanali A, Lwande W. Mosquitoes Larvicidal Activity of Ocimum kilimandscharicum Oil Formulation under Laboratory and Field-Simulated Conditions. Insects. 2022;13(2). Epub 2022/02/26. doi: 10.3390/insects13020203. PubMed PMID: 35206778; PubMed Central PMCID: PMCPMC8877965.
103. Ofosu-Appiah LH, Amelor DK, Ayensu B, Akyereko E, Rabiwu NI, Opare D, et al. An evaluation of the diagnostic performance characteristics of the Yellow Fever IgM immunochromatographic rapid diagnostic test kit from SD Biosensor in Ghana. PloS one. 2022;17(1):e0262312. Epub 2022/01/08. doi: 10.1371/journal.pone.0262312. PubMed PMID: 34995319; PubMed Central PMCID: PMCPMC8741057.
104. Oladepo O, Dipeolu IO, Oladunni O. Outcome of reminder text messages intervention on completion of routine immunization in rural areas, Nigeria. Health promotion international. 2020. Epub 2020/11/03. doi: 10.3389/fimmu.2020.577751

10.1093/heapro/daaa092. PubMed PMID: 33057615.
105. Olivero RM, Hamer DH, MacLeod WB, Benoit CM, Sanchez-Vegas C, Jentes ES, et al. Dengue Virus Seroconversion in Travelers to Dengue-Endemic Areas. American Journal of Tropical Medicine and Hygiene. 2016;95(5):1130-6. PubMed PMID: CCC:000400206600032.
106. Omololu A, Shehu N, Shwe D, Onukak A, Andrew G, Gomerep S. Yellow fever childhood immunization coverage in Jos North Local Government area, North Central Nigeria: 2015–2017. International Journal of Infectious Diseases. 2020;101:509-10. doi: 10.1016/j.ijid.2020.09.1323.
107. Onyango CO, Grobbelaar AA, Gibson GV, Sang RC, Sow A, Swaneopel R, et al. Yellow fever outbreak, southern Sudan, 2003. Emerging infectious diseases. 2004;10(9):1668-70. Epub 2004/10/23. doi: 10.3201/eid1009.030727. PubMed PMID: 15498174; PubMed Central PMCID: PMCPmc3320285.
108. Onyango CO, Ofula VO, Sang RC, Konongoi SL, Sow A, De Cock KM, et al. Yellow fever outbreak, Imatong, southern Sudan. Emerging infectious diseases. 2004;10(6):1063-8. Epub 2004/06/23. doi: 10.3201/eid1006.030738. PubMed PMID: 15207058; PubMed Central PMCID: PMCPmc3323161.
109. Phan MV, Murad SD, van der Eijk AA, Metselaar HJ, Hartog H, Harinck F, et al. Genomic sequence of yellow fever virus from a Dutch traveller returning from the Gambia-Senegal region, the Netherlands, November 2018. Euro surveillance : bulletin Europeen sur les maladies transmissibles = European communicable disease bulletin. 2019;24(4). Epub 2019/02/12. doi: 10.1016/j.puhe.2018.12.015

10.2807/1560-7917.es.2019.24.4.1800684. PubMed PMID: 30696531; PubMed Central PMCID: PMCPmc6351999.
110. Phoutrides EK, Coulibaly MB, George CM, Sacko A, Traore S, Bessoff K, et al. Dengue virus seroprevalence among febrile patients in Bamako, Mali: results of a 2006 surveillance study. Vector borne and zoonotic diseases (Larchmont, NY). 2011;11(11):1479-85. Epub 2011/07/20. doi: 10.1089/vbz.2011.0622. PubMed PMID: 21767159.
111. Ramos Junior AN, Heukelbach J. Yellow fever risk assessment in the Central African Republic. Transactions of the Royal Society of Tropical Medicine and Hygiene. 2015;109(4):231-2. Epub 2015/03/04. doi: 10.1093/trstmh/trv011. PubMed PMID: 25732754.
112. Raulino R, Thaurignac G, Butel C, Villabona-Arenas CJ. Multiplex detection of antibodies to Chikungunya, O'nyong-nyong, Zika, Dengue, West Nile and Usutu viruses in

diverse non-human primate species from Cameroon and the Democratic Republic of Congo. 2021;15(1):e0009028. doi: 10.1371/journal.pntd.0009028. PubMed PMID: 33476338.
113. Rojas A, Diagne CT, Stittleburg VD, Mohamed-Hadley A, de Guillén YA, Balmaseda A, et al. Internally Controlled, Multiplex Real-Time Reverse Transcription PCR for Dengue Virus and Yellow Fever Virus Detection. The American journal of tropical medicine and hygiene. 2018;98(6):1833-6. Epub 2018/04/04. doi: 10.4269/ajtmh.18-0024. PubMed PMID: 29611509; PubMed Central PMCID: PMCPmc6086146.
114. Rossetto EV, Luna EJ. REPORTING DELAY DURING THE YELLOW FEVER OUTBREAK, ANGOLA, 2016. Revista do Instituto de Medicina Tropical de Sao Paulo. 2016;58:91. Epub 2016/12/17. doi: 10.1590/s1678-9946201658091. PubMed PMID: 27982357; PubMed Central PMCID: PMCPmc5147721.
115. Rubbo PA, Soupé-Gilbert ME, Golongba DM, Mbombo F, Girault D, Nakouné E, et al. Evidence of human leptospirosis cases in a cohort of febrile patients in Bangui, Central African Republic: a retrospective study, 2012-2015. BMC infectious diseases. 2018;18(1):376. Epub 2018/08/09. doi: 10.1186/s12879-018-3298-z. PubMed PMID: 30086725; PubMed Central PMCID: PMCPmc6081884.
116. Sánchez-Seco MP, Rosario D, Hernández L, Domingo C, Valdés K, Guzmán MG, et al. Detection and subtyping of dengue 1-4 and yellow fever viruses by means of a multiplex RT-nested-PCR using degenerated primers. Tropical medicine & international health : TM & IH. 2006;11(9):1432-41. Epub 2006/08/26. doi: 10.1111/j.1365-3156.2006.01696.x. PubMed PMID: 16930266.
117. Scheck MK, Lehmann L, Zaucha M, Schwarzlmueller P, Huber K, Pritsch M, et al. FluoRNT: A robust, efficient assay for the detection of neutralising antibodies against yellow fever virus 17D. PloS one. 2022;17(2):e0262149. Epub 2022/02/10. doi: 10.1371/journal.pone.0262149. PubMed PMID: 35139078; PubMed Central PMCID: PMCPMC8827462.
118. Selemane I. Epidemiological monitoring of the last outbreak of yellow fever in Brazil - An outlook from Portugal. Travel medicine and infectious disease. 2019;28:46-51. Epub 2018/12/26. doi: 10.1016/j.tmaid.2018.12.008. PubMed PMID: 30583006.
119. Shi L, Fu S, Wang L, Li X, Gu D, Liu C, et al. Surveillance of mosquito-borne infectious diseases in febrile travelers entering China via Shenzhen ports, China, 2013. Travel medicine and infectious disease. 2016;14(2):123-30. Epub 2016/03/11. doi: 10.1016/j.tmaid.2016.02.002. PubMed PMID: 26960752.
120. Simões M, Camacho LA, Yamamura AM, Miranda EH, Cajaraville AC, da Silva Freire M. Evaluation of accuracy and reliability of the plaque reduction neutralization test (micro-PRNT) in detection of yellow fever virus antibodies. Biologicals : journal of the International Association of Biological Standardization. 2012;40(6):399-404. Epub 2012/10/05. doi: 10.1016/j.biologicals.2012.09.005. PubMed PMID: 23034357.
121. Simon-Loriere E, Faye O, Prot M, Casademont I, Fall G, Fernandez-Garcia MD, et al. Autochthonous Japanese Encephalitis with Yellow Fever Coinfection in Africa. The New England journal of medicine. 2017;376(15):1483-5. Epub 2017/04/14. doi: 10.1056/NEJMc1701600. PubMed PMID: 28402771.
122. Soghaier MA, Hagar A, Abbas MA, Elmangory MM, Eltahir KM, Sall AA. Yellow Fever outbreak in Darfur, Sudan in October 2012; the initial outbreak investigation report. Journal of infection and public health. 2013;6(5):370-6. Epub 2013/09/04. doi: 10.1016/j.jiph.2013.04.007. PubMed PMID: 23999341.

123. Sow SO, Chowdhury PR, Niedrig M, Saathoff E, Kampmann B, Seruyange E, et al. Seroreactivity to Chikungunya and West Nile Viruses in Rwandan Blood Donors. Expert review of vaccines. 2019;19(10):731-40. Epub 2019/07/05. doi: 10.1080/14760584.2019.1640118

10.1089/vbz.2018.2393. PubMed PMID: 31246538.
124. Stock NK, Escadafal C, Achazi K, Cissé M, Niedrig M. Development and characterization of polyclonal peptide antibodies for the detection of Yellow fever virus proteins. Journal of virological methods. 2015;222:110-6. Epub 2015/06/19. doi: 10.1016/j.jviromet.2015.06.006. PubMed PMID: 26086983.
125. Stock NK, Laraway H, Faye O, Diallo M, Niedrig M, Sall AA. Biological and phylogenetic characteristics of yellow fever virus lineages from West Africa. Journal of virology. 2013;87(5):2895-907. Epub 2012/12/28. doi: 10.1128/jvi.01116-12. PubMed PMID: 23269797; PubMed Central PMCID: PMCPmc3571399.
126. Sutherland LJ, Cash AA, Huang YJ, Sang RC, Malhotra I, Moormann AM, et al. Serologic evidence of arboviral infections among humans in Kenya. The American journal of tropical medicine and hygiene. 2011;85(1):158-61. Epub 2011/07/08. doi: 10.4269/ajtmh.2011.10-0203. PubMed PMID: 21734142; PubMed Central PMCID: PMCPmc3122361.
127. Tricou V, Bouscaillou J, Laghoe-Nguembe GL, Béré A, Konamna X, Sélékon B, et al. Hepatitis E virus outbreak associated with rainfall in the Central African Republic in 2008-2009. mBio. 2020;20(1):260. Epub 2020/04/09. doi: 10.1128/mBio.02494-19

10.1186/s12879-020-04961-4. PubMed PMID: 32245368; PubMed Central PMCID: PMCPmc7157777

Pmc7119096.
128. Trindade GF, de Lima SMB, Britto C, Fernandes-Monteiro AG. Detection of Yellow Fever Virus by Quantitative Real-Time PCR (qPCR). PLoS neglected tropical diseases. 2020;2065:65-77. Epub 2019/10/08. doi: 10.1371/journal.pntd.0007783

10.1007/978-1-4939-9833-3_6. PubMed PMID: 31578688; PubMed Central PMCID: PMCPmc6797215.
129. Umoke PCI, Umoke M, Eyo N, Ugwu Mbbs A, Okeke E, Nwalieji CA, et al. Delay in health-seeking behaviour: Implication to yellow fever outcome in the 2019 outbreak in Nigeria. Health & social care in the community. 2021;29(3):703-11. Epub 2021/03/25. doi: 10.1111/hsc.13329. PubMed PMID: 33761167.
130. Vianello S, Silva de Souza G, Maia M, Belfort R, Jr., de Oliveira Dias JR. Ocular Findings in Yellow Fever Infection. JAMA ophthalmology. 2019;137(3):300-4. Epub 2019/01/11. doi: 10.1001/jamaophthalmol.2018.6408. PubMed PMID: 30629101; PubMed Central PMCID: PMCPmc6439724.
131. von Lindern JJ, Aroner S, Barrett ND, Wicker JA, Davis CT, Barrett ADT. Genome analysis and phylogenetic relationships between east, central and west African isolates of Yellow fever virus. The Journal of general virology. 2006;87(Pt 4):895-907. Epub 2006/03/11. doi: 10.1099/vir.0.81236-0. PubMed PMID: 16528039.
132. Wat'senga Tezzo F, Fasine S, Manzambi Zola E, Marquetti MDC, Binene Mbuka G, Ilombe G, et al. High Aedes spp. larval indices in Kinshasa, Democratic Republic of Congo. Memorias do Instituto Oswaldo Cruz. 2021;14(1):92. Epub 2021/02/04. doi: 10.1590/0074-02760200313

10.1186/s13071-021-04588-7. PubMed PMID: 33522947; PubMed Central PMCID: PMCPmc7849183.

133. WHO. Yellow fever, Senegal (update). Releve epidemiologique hebdomadaire. 2002;77(45):373-4. Epub 2002/11/22. PubMed PMID: 12442552.
134. WHO. Yellow fever, Senegal--update. Releve epidemiologique hebdomadaire. 2002;77(42):349. Epub 2002/11/01. PubMed PMID: 12407817.
135. WHO. Outbreak news. Yellow fever, Cote d'Ivoire. Releve epidemiologique hebdomadaire. 2006;81(43):410-. PubMed PMID: MEDLINE:17072996.
136. WHO. Outbreak news. Yellow fever, Togo--update. Releve epidemiologique hebdomadaire. 2007;82(7):50. Epub 2007/02/21. PubMed PMID: 17310514.
137. WHO. Outbreak news. Yellow fever, Togo. Releve epidemiologique hebdomadaire. 2007;82(5):33-4. Epub 2007/02/06. PubMed PMID: 17278254.
138. WHO. Outbreak news. Yellow fever, Guinea. Releve epidemiologique hebdomadaire. 2008;83(40):358-9. Epub 2008/10/07. PubMed PMID: 18837116.
139. WHO. Outbreak news. Yellow fever, Liberia. Releve epidemiologique hebdomadaire. 2008;83(18):158. Epub 2008/05/06. PubMed PMID: 18453065.
140. WHO. Outbreak news. Yellow fever, Republic of the Congo. Releve epidemiologique hebdomadaire. 2009;84(19):161. Epub 2009/05/12. PubMed PMID: 19425251.
141. WHO. Yellow fever surveillance and outbreak response: revision of case definitions, October 2010. Releve epidemiologique hebdomadaire. 2010;85(47):465-72. Epub 2010/11/26. PubMed PMID: 21090394.
142. WHO. Yellow fever, Senegal. Releve epidemiologique hebdomadaire. 2010;85(41):401-2. Epub 2010/10/14. PubMed PMID: 20939151.
143. WHO. Outbreak news. Yellow fever, Sierra Leone. Releve epidemiologique hebdomadaire. 2011;86(12):101-2. Epub 2011/03/29. PubMed PMID: 21442796.
144. WHO. Outbreak news. Yellow fever, Uganda. Releve epidemiologique hebdomadaire. 2011;86(5):37-8. Epub 2011/02/09. PubMed PMID: 21302384.
145. WHO. Outbreak news. Yellow fever, Cote dIvoire -update. Releve epidemiologique hebdomadaire. 2011;86(6):45-. PubMed PMID: MEDLINE:21299034.
146. WHO. Outbreak news. Yellow fever, Cote dIvoire. Releve epidemiologique hebdomadaire. 2011;86(5):37-. PubMed PMID: MEDLINE:21299032.
147. WHO. Outbreak news. Yellow fever, Sudan. Releve epidemiologique hebdomadaire. 2012;87(46):449. Epub 2012/12/06. PubMed PMID: 23210145.
148. WHO. Outbreak news. Yellow fever, Sudan - update. Releve epidemiologique hebdomadaire. 2012;87(48):477-. PubMed PMID: MEDLINE:23210148.
149. Woodall JP. Another pandemic disaster looms: yellow fever spreading from Angola. The Pan African medical journal. 2016;24:107. Epub 2016/09/20. doi: 10.11604/pamj.2016.24.107.9921. PubMed PMID: 27642446; PubMed Central PMCID: PMCPmc5012773.
150. Woodall JP, Yuill TM. Why is the yellow fever outbreak in Angola a 'threat to the entire world'? International journal of infectious diseases : IJID : official publication of the International Society for Infectious Diseases. 2016;48:96-7. Epub 2016/05/11. doi: 10.1016/j.ijid.2016.05.001. PubMed PMID: 27163382.
151. Yang T-C, Casati S, Flacio E, Caminada AP, Ruggeri-Bernardi N, Demarta A, et al. Detection of Chikungunya Virus and Arboviruses in Mosquito Vectors. Journal of Entomological Science. 2010;45(3):272-82. doi: 10.18474/0749-8004-45.3.272. PubMed PMID: CCC:000280019600009.
152. Yao W, Yang Z, Lou X, Mao H, Yan H, Zhang Y. Simultaneous Detection of Ebola Virus and Pathogens Associated With Hemorrhagic Fever by an Oligonucleotide Microarray.

Frontiers in microbiology. 2021;12:713372. Epub 2021/08/17. doi: 10.3389/fmicb.2021.713372. PubMed PMID: 34394063; PubMed Central PMCID: PMCPMC8363200.
153. Yen TY, Trovoada dos Santos Mde J, Tseng LF, Chang SF, Cheng CF, Carvalho AV, et al. Seroprevalence of antibodies against dengue virus among pregnant women in the Democratic Republic of Sao Tome and Principe. Acta tropica. 2016;155:58-62. Epub 2016/01/08. doi: 10.1016/j.actatropica.2015.12.012. PubMed PMID: 26739653.
154. Yuill TM, Woodall JP, Baekeland S. Latest outbreak news from ProMED-mail. Yellow fever outbreak-Darfur Sudan and Chad. International Journal of Infectious Diseases. 2013;17(7):E476-E8. PubMed PMID: CCC:000318976300001.
155. Zhang Y, Ye F, Xia LX, Zhu LW, Kamara IL, Huang KQ, et al. Next-generation Sequencing Study of Pathogens in Serum from Patients with Febrile Jaundice in Sierra Leone. Biomedical and environmental sciences : BES. 2019;32(5):363-70. Epub 2019/06/28. doi: 10.1089/vbz.2018.2393

10.3967/bes2019.048. PubMed PMID: 31217053.
156. Zhao S, Musa SS, Hebert JT, Cao P, Ran J, Meng J, et al. Modelling the effective reproduction number of vector-borne diseases: the yellow fever outbreak in Luanda, Angola 2015-2016 as an example. PeerJ. 2020;8:e8601. Epub 2020/03/15. doi: 10.3390/vaccines8010124

10.7717/peerj.8601. PubMed PMID: 32149023; PubMed Central PMCID: PMCPmc7049463.
157. Zhao S, Stone L, Gao D, He D. Modelling the large-scale yellow fever outbreak in Luanda, Angola, and the impact of vaccination. International journal of environmental research and public health. 2018;12(1):e0006158. Epub 2018/02/01. doi: 10.3390/ijerph15020220

10.1371/journal.pntd.0006158. PubMed PMID: 29338001; PubMed Central PMCID: PMCPmc5858289.
