# Supplementary material for: Epidemiology of yellow fever virus in humans, arthropods, and non-human primates in sub-Saharan Africa: A systematic review and meta-analysis
Source: PLoS Negl Trop Dis. 2022 Jul 22;16(7):e0010610. doi: 10.1371/journal.pntd.0010610 (PMC9307179; doi:10.1371/journal.pntd.0010610)
Supplement: S5 Table — (PDF) [file pntd.0010610.s005.pdf]

S5 Table: Risk of bias assessment

Authors	Was the study's target population a close representation of the national population in relation to YFV prevalence or CFR?	Was the sampling frame a true or close representation of the target population?	Was some form of random selection used to select the sample, OR was acensus undertaken?	Were data collected directly from the subjects (as opposed to a proxy)?	Was an acceptable inclusion criteria definition used in the study?	Did the author calculate and respect the expected sample size?	Was the YFV detection assay shown to have reliability and validity?	Was the same mode of data collection used for all subjects?	Was the length of the study period > or = 1 year?	Were the numerator(s) and denominator(s) for the YFV prevalence appropriate?	Risk of bias	Species
Agwu, 2016	No	Yes	No	Not applicable	Yes	No	Yes	Yes	Unclear	Yes	Moderate risk of bias	Individual Mosquitoes
Ahmed, 2016	No	Yes	No	Yes	Yes	No	Yes	Yes	No	Yes	Moderate risk of bias	Humans
Ahmed, 2016	No	Yes	No	Yes	Yes	No	Yes	Yes	No	Yes	Moderate risk of bias	Humans
Akelew, 2022	No	Yes	No	Yes	Yes	No	Yes	Yes	Unclear	Yes	Moderate risk of bias	Humans
Akoua-Koffi, 2001	No	Yes	No	Yes	Yes	No	Yes	No	No	Yes	Moderate risk of bias	Humans
Akoua-Koffi, 2001	No	Yes	No	Yes	Yes	No	Yes	No	No	Yes	Moderate risk of bias	Humans
Akoua-Koffi, 2001	No	Yes	No	Yes	Yes	No	Yes	No	No	Yes	Moderate risk of bias	Humans
Akoua-Koffi, 2001	No	Yes	No	Yes	Yes	No	Yes	No	No	Yes	Moderate risk of bias	Humans
Akoua-Koffi, 2002	No	Yes	No	Yes	Yes	No	Yes	Yes	No	Yes	Moderate risk of bias	Humans
Akoua-Koffi, 2002	No	Yes	No	Yes	Yes	No	Yes	Yes	No	Yes	Moderate risk of bias	Humans
Akoua-Koffi, 2002	No	Yes	No	Yes	Yes	No	Yes	Yes	No	Yes	Moderate risk of bias	Humans
Akoua-Koffi, 2002	No	Yes	No	Yes	Yes	No	Yes	Yes	No	Yes	Moderate risk of bias	Humans
Akoua-Koffi, 2014	No	Yes	No	Yes	Yes	No	Yes	Yes	No	Yes	Moderate risk of bias	Humans
Akoua-Koffi, 2014	No	Yes	No	Yes	Yes	No	Yes	Yes	No	Yes	Moderate risk of bias	Humans
Akoua-Koffi, 2014	No	Yes	No	Yes	Yes	No	Yes	Yes	No	Yes	Moderate risk of bias	Humans
Alhakimi, 2015	No	Yes	No	Yes	Yes	No	Yes	Yes	No	Yes	Moderate risk of bias	Humans
Asebe, 2021	No	Yes	Yes	Yes	Yes	No	Yes	Yes	No	Yes	Low risk of bias	Humans
Baba, 2021	No	Yes	No	Yes	Yes	No	Yes	Yes	Unclear	Yes	Moderate risk of bias	Humans
Babaniyi, 2015	No	Yes	No	Yes	Yes	Yes	Yes	Yes	Unclear	Yes	Low risk of bias	Humans
Babaniyi, 2015	No	Yes	No	Yes	Yes	Yes	Yes	Yes	Unclear	Yes	Low risk of bias	Humans
Caux, 2020	No	Yes	No	Yes	Yes	No	Yes	Yes	Unclear	Yes	Moderate risk of bias	Humans
Chepkorir, 2019	No	Yes	Yes	Yes	Yes	No	Yes	Yes	Yes	Yes	Low risk of bias	Humans
Diagne, 2021	No	Yes	No	Yes	Yes	No	Yes	Yes	Yes	Yes	Low risk of bias	Humans
Diagne, 2021	No	Yes	No	Yes	Yes	No	Yes	Yes	Yes	Yes	Low risk of bias	Individual Mosquitoes
Diagne, 2021	No	Yes	No	Yes	Yes	No	Yes	Yes	Yes	Yes	Low risk of bias	Pooled population
Diallo, 2014	No	Yes	No	Not applicable	Yes	No	Yes	Yes	Yes	Yes	Moderate risk of bias	Pooled population
Diallo, 2020	No	Yes	No	Yes	Yes	No	Yes	Yes	No	Yes	Moderate risk of bias	Humans
Diallo, 2020	No	Yes	No	Yes	Yes	No	Yes	Yes	No	Yes	Moderate risk of bias	Humans
Diallo, 2020	No	Yes	No	Not applicable	Yes	No	Yes	Yes	No	Yes	Moderate risk of bias	Individual Mosquitoes
Diallo, 2020	No	Yes	No	Not applicable	Yes	No	Yes	Yes	No	Yes	Moderate risk of bias	Individual Mosquitoes
Diallo, 2020	No	Yes	No	Not applicable	Yes	No	Yes	Yes	No	Yes	Moderate risk of bias	Individual Mosquitoes
Diallo, 2020	No	Yes	No	Not applicable	Yes	No	Yes	Yes	No	Yes	Moderate risk of bias	Individual Mosquitoes
Diallo, 2020	No	Yes	No	Not applicable	Yes	No	Yes	Yes	No	Yes	Moderate risk of bias	Individual Mosquitoes
Diallo, 2020	No	Yes	No	Not applicable	Yes	No	Yes	Yes	No	Yes	Moderate risk of bias	Individual Mosquitoes
Diallo, 2020	No	Yes	No	Not applicable	Yes	No	Yes	Yes	No	Yes	Moderate risk of bias	Individual Mosquitoes
Diallo, 2020	No	Yes	No	Not applicable	Yes	No	Yes	Yes	No	Yes	Moderate risk of bias	Individual Mosquitoes
Diallo, 2020	No	Yes	No	Not applicable	Yes	No	Yes	Yes	No	Yes	Moderate risk of bias	Individual Mosquitoes
Diallo, 2020	No	Yes	No	Not applicable	Yes	No	Yes	Yes	No	Yes	Moderate risk of bias	Individual Mosquitoes
Diallo, 2020	No	Yes	No	Not applicable	Yes	No	Yes	Yes	No	Yes	Moderate risk of bias	Individual Mosquitoes
Diallo, 2020	No	Yes	No	Not applicable	Yes	No	Yes	Yes	No	Yes	Moderate risk of bias	Individual Mosquitoes
Diallo, 2020	No	Yes	No	Not applicable	Yes	No	Yes	Yes	No	Yes	Moderate risk of bias	Individual Mosquitoes
Diallo, 2020	No	Yes	No	Not applicable	Yes	No	Yes	Yes	No	Yes	Moderate risk of bias	Individual Mosquitoes
Diallo, 2020	No	Yes	No	Not applicable	Yes	No	Yes	Yes	No	Yes	Moderate risk of bias	Individual Mosquitoes
Diallo, 2020	No	Yes	No	Not applicable	Yes	No	Yes	Yes	No	Yes	Moderate risk of bias	Individual Mosquitoes
Diallo, 2020	No	Yes	No	Not applicable	Yes	No	Yes	Yes	No	Yes	Moderate risk of bias	Individual Mosquitoes
Diallo, 2020	No	Yes	No	Not applicable	Yes	No	Yes	Yes	No	Yes	Moderate risk of bias	Individual Mosquitoes
Diallo, 2020	No	Yes	No	Not applicable	Yes	No	Yes	Yes	No	Yes	Moderate risk of bias	Individual Mosquitoes
Diallo, 2020	No	Yes	No	Not applicable	Yes	No	Yes	Yes	No	Yes	Moderate risk of bias	Individual Mosquitoes

Diallo, 2020	No	Yes	No	Not applicable	Yes	No	Yes	Yes	No	Yes	Moderate risk of bias	Individual Mosquitoes
Diallo, 2020	No	Yes	No	Not applicable	Yes	No	Yes	Yes	No	Yes	Moderate risk of bias	Individual Mosquitoes
Diallo, 2020	No	Yes	No	Not applicable	Yes	No	Yes	Yes	No	Yes	Moderate risk of bias	Individual Mosquitoes
Diallo, 2020	No	Yes	No	Not applicable	Yes	No	Yes	Yes	No	Yes	Moderate risk of bias	Individual Mosquitoes
Diallo, 2020	No	Yes	No	Not applicable	Yes	No	Yes	Yes	No	Yes	Moderate risk of bias	Individual Mosquitoes
Diallo, 2020	No	Yes	No	Not applicable	Yes	No	Yes	Yes	No	Yes	Moderate risk of bias	Individual Mosquitoes
Diallo, 2020	No	Yes	No	Not applicable	Yes	No	Yes	Yes	No	Yes	Moderate risk of bias	Individual Mosquitoes
Diallo, 2020	No	Yes	No	Not applicable	Yes	No	Yes	Yes	No	Yes	Moderate risk of bias	Individual Mosquitoes
Diallo, 2020	No	Yes	No	Not applicable	Yes	No	Yes	Yes	No	Yes	Moderate risk of bias	Individual Mosquitoes
Diallo, 2020	No	Yes	No	Not applicable	Yes	No	Yes	Yes	No	Yes	Moderate risk of bias	Individual Mosquitoes
Diallo, 2020	No	Yes	No	Not applicable	Yes	No	Yes	Yes	No	Yes	Moderate risk of bias	Individual Mosquitoes
Ekenna, 2010	No	Yes	No	Yes	Yes	No	Yes	Yes	No	Yes	Moderate risk of bias	Humans
Endale, 2020	No	Yes	Yes	Yes	Yes	Yes	Yes	Yes	No	Yes	Low risk of bias	Humans
Farnon, 2010	No	Yes	Yes	Yes	Yes	No	Yes	Yes	No	Yes	Low risk of bias	Humans
Faye, 2007	No	Yes	No	Yes	Yes	No	Yes	Yes	Unclear	Yes	Moderate risk of bias	Humans
Faye, 2007	No	Yes	No	Not applicable	Yes	No	Yes	Yes	Unclear	Yes	Moderate risk of bias	Individual Mosquitoes
Faye, 2007	No	Yes	No	Not applicable	Yes	No	Yes	Yes	Unclear	Yes	Moderate risk of bias	Individual Mosquitoes
Faye, 2007	No	Yes	No	Not applicable	Yes	No	Yes	Yes	Unclear	Yes	Moderate risk of bias	Pooled population
Fokam, 2010	No	Yes	No	Yes	Yes	No	Yes	Yes	Unclear	Yes	Moderate risk of bias	Humans
Fokam, 2010	No	Yes	No	Yes	Yes	No	Yes	Yes	Unclear	Yes	Moderate risk of bias	Humans
Gould, 2008	No	Yes	No	Yes	Yes	No	Yes	Yes	No	Yes	Moderate risk of bias	Humans
Gould, 2008	No	Yes	No	Yes	Yes	No	Yes	Yes	No	Yes	Moderate risk of bias	Humans
Ingelbeen, 2018	No	Yes	No	Yes	Yes	No	Yes	Yes	No	Yes	Moderate risk of bias	Humans
Ingelbeen, 2018	No	Yes	No	Yes	Yes	No	Yes	Yes	No	Yes	Moderate risk of bias	Humans
Inziani, 2020	No	Yes	No	Yes	Yes	No	Yes	Yes	No	Yes	Moderate risk of bias	Humans
Jentes, 2010	No	Yes	No	Yes	Yes	No	Yes	Yes	No	Yes	Moderate risk of bias	Humans
Kading, 2013	No	Yes	No	Not applicable	Yes	No	Yes	Yes	Yes	Yes	Moderate risk of bias	Non-human primates
Kading, 2013	No	Yes	No	Not applicable	Yes	No	Yes	Yes	Yes	Yes	Moderate risk of bias	Non-human primates
Kading, 2013	No	Yes	No	Not applicable	Yes	No	Yes	Yes	Yes	Yes	Moderate risk of bias	Other animal species
Kading, 2013	No	Yes	No	Not applicable	Yes	No	Yes	Yes	Yes	Yes	Moderate risk of bias	Other animal species
Kading, 2013	No	Yes	No	Not applicable	Yes	No	Yes	Yes	Yes	Yes	Moderate risk of bias	Other animal species
Kading, 2018	No	Yes	No	Not applicable	Yes	No	Yes	Yes	Yes	Yes	Moderate risk of bias	Other animal species
Kayiwa, 2018	No	Yes	No	Yes	Yes	No	Yes	Yes	Yes	Yes	Low risk of bias	Humans
Konongoi, 2016	No	Yes	No	Yes	Yes	No	Yes	Yes	Yes	Yes	Low risk of bias	Humans
Kuniholm, 2016	No	Yes	No	Yes	Yes	No	Yes	Yes	Yes	Yes	Low risk of bias	Humans
Kwagonza, 2018	No	Yes	No	Yes	Yes	No	Yes	Yes	No	Yes	Moderate risk of bias	Humans
Kwagonza, 2018	No	Yes	No	Yes	Yes	No	Yes	Yes	No	Yes	Moderate risk of bias	Humans
Kwagonza, 2018	No	Yes	No	Not applicable	Yes	No	Yes	Yes	No	Yes	Moderate risk of bias	Individual Mosquitoes
Kwallah, 2015	No	Yes	No	Yes	Yes	No	Yes	Yes	Yes	Yes	Low risk of bias	Humans
Kwallah, 2015	No	Yes	No	Yes	Yes	No	Yes	Yes	Yes	Yes	Low risk of bias	Humans
Lilay, 2017	No	Yes	No	Yes	Yes	No	Yes	Yes	No	Yes	Moderate risk of bias	Humans
Mease, 2011	No	Yes	No	Yes	Yes	No	Yes	Yes	Unclear	Yes	Moderate risk of bias	Humans
Mengesha Tsegaye, 2018	No	Yes	Yes	Yes	Yes	Yes	Yes	Yes	No	Yes	Low risk of bias	Humans
Mohamed, 2019	No	Yes	No	Yes	Yes	No	Yes	Yes	No	Yes	Moderate risk of bias	Humans
Mulchandani, 2019	No	Yes	No	Not applicable	Yes	No	Yes	Yes	No	Yes	Moderate risk of bias	Individual Mosquitoes
Nakounne, 2001	No	Yes	No	Yes	Yes	Unclear	Yes	Unclear	Yes	Yes	Moderate risk of bias	Humans
Nakounne, 2001	No	Yes	No	Yes	Yes	Unclear	Yes	Unclear	Yes	Yes	Moderate risk of bias	Humans
Ngoagouni, 2012	No	Yes	No	Not applicable	Yes	No	Yes	No	Yes	Yes	Moderate risk of bias	Individual Mosquitoes
Ngoagouni, 2012	No	Yes	No	Not applicable	Yes	No	Yes	No	Yes	Yes	Moderate risk of bias	Individual Mosquitoes
Ngoagouni, 2012	No	Yes	No	Not applicable	Yes	No	Yes	No	Yes	Yes	Moderate risk of bias	Individual Mosquitoes
Ngoagouni, 2012	No	Yes	No	Not applicable	Yes	No	Yes	No	Yes	Yes	Moderate risk of bias	Individual Mosquitoes
Ngoagouni, 2012	No	Yes	No	Not applicable	Yes	No	Yes	No	Yes	Yes	Moderate risk of bias	Individual Mosquitoes
Ngoagouni, 2012	No	Yes	No	Not applicable	Yes	No	Yes	No	Yes	Yes	Moderate risk of bias	Individual Mosquitoes

Ngoagouni, 2012	No	Yes	No	Not applicable	Yes	No	Yes	No	Yes	Yes	Moderate risk of bias	Individual Mosquitoes
Ngoagouni, 2012	No	Yes	No	Not applicable	Yes	No	Yes	No	Yes	Yes	Moderate risk of bias	Individual Mosquitoes
Ngoagouni, 2012	No	Yes	No	Not applicable	Yes	No	Yes	No	Yes	Yes	Moderate risk of bias	Individual Mosquitoes
Ngoagouni, 2012	No	Yes	No	Not applicable	Yes	No	Yes	No	Yes	Yes	Moderate risk of bias	Individual Mosquitoes
Ngoagouni, 2012	No	Yes	No	Not applicable	Yes	No	Yes	No	Yes	Yes	Moderate risk of bias	Individual Mosquitoes
Ngoagouni, 2012	No	Yes	No	Not applicable	Yes	No	Yes	No	Yes	Yes	Moderate risk of bias	Individual Mosquitoes
Ngoagouni, 2012	No	Yes	No	Not applicable	Yes	No	Yes	No	Yes	Yes	Moderate risk of bias	Individual Mosquitoes
Ngoagouni, 2012	No	Yes	No	Not applicable	Yes	No	Yes	No	Yes	Yes	Moderate risk of bias	Individual Mosquitoes
Ngoagouni, 2012	No	Yes	No	Not applicable	Yes	No	Yes	No	Yes	Yes	Moderate risk of bias	Individual Mosquitoes
Ngoagouni, 2012	No	Yes	No	Not applicable	Yes	No	Yes	No	Yes	Yes	Moderate risk of bias	Individual Mosquitoes
Ngoagouni, 2012	No	Yes	No	Not applicable	Yes	No	Yes	No	Yes	Yes	Moderate risk of bias	Individual Mosquitoes
Ngoagouni, 2012	No	Yes	No	Not applicable	Yes	No	Yes	No	Yes	Yes	Moderate risk of bias	Individual Mosquitoes
Ngoagouni, 2012	No	Yes	No	Not applicable	Yes	No	Yes	No	Yes	Yes	Moderate risk of bias	Individual Mosquitoes
Ngoagouni, 2012	No	Yes	No	Not applicable	Yes	No	Yes	No	Yes	Yes	Moderate risk of bias	Individual Mosquitoes
Ngoagouni, 2012	No	Yes	No	Not applicable	Yes	No	Yes	No	Yes	Yes	Moderate risk of bias	Individual Mosquitoes
Ngoagouni, 2012	No	Yes	No	Not applicable	Yes	No	Yes	No	Yes	Yes	Moderate risk of bias	Individual Mosquitoes
Ngoagouni, 2012	No	Yes	No	Not applicable	Yes	No	Yes	No	Yes	Yes	Moderate risk of bias	Individual Mosquitoes
Ngoagouni, 2012	No	Yes	No	Not applicable	Yes	No	Yes	No	Yes	Yes	Moderate risk of bias	Individual Mosquitoes
Nwachukwu, 2020	No	Yes	No	Yes	Yes	No	Yes	Yes	No	Yes	Moderate risk of bias	Humans
Nwachukwu, 2020	No	Yes	No	Yes	Yes	No	Yes	Yes	No	Yes	Moderate risk of bias	Humans
Onyango, 2004	No	Yes	No	Yes	Yes	No	Yes	Yes	No	Yes	Moderate risk of bias	Humans
Otshudiema, 2017	No	Yes	No	Yes	Yes	No	Yes	Yes	No	Yes	Moderate risk of bias	Humans
Otshudiema, 2017	No	Yes	No	Yes	Yes	No	Yes	Yes	No	Yes	Moderate risk of bias	Humans
Otshudiema, 2017	No	Yes	No	Yes	Yes	No	Yes	Yes	No	Yes	Moderate risk of bias	Humans
Proesmans, 2019	No	Yes	No	Yes	Yes	No	Yes	Yes	No	Yes	Moderate risk of bias	Humans
Rachas, 2014	No	Yes	No	Yes	Yes	No	Yes	Yes	Yes	Yes	Low risk of bias	Humans
Rachas, 2014	No	Yes	No	Yes	Yes	No	Yes	Yes	Yes	Yes	Low risk of bias	Humans
Rugarabamu, 2021	No	Yes	Yes	Yes	Yes	Yes	Yes	Yes	No	Yes	Low risk of bias	Humans
Rugarabamu, 2021	No	Yes	Yes	Yes	Yes	Yes	Yes	Yes	No	Yes	Low risk of bias	Humans
Schoepp, 2014	No	Yes	No	Yes	Yes	No	Yes	Unclear	Yes	Yes	Moderate risk of bias	Humans
Simo Tchetgna, 2018	No	Yes	Yes	Yes	Yes	No	Yes	Yes	Yes	Yes	Low risk of bias	Humans
Sow, 2016	No	Yes	No	Yes	Yes	No	Yes	Yes	Yes	Yes	Low risk of bias	Humans
Sow, 2016	No	Yes	No	Yes	Yes	No	Yes	Yes	Yes	Yes	Low risk of bias	Humans
Staples, 2014	No	Yes	Yes	Yes	Yes	Yes	Yes	Yes	No	Yes	Low risk of bias	Humans
Staples, 2014	No	Yes	Yes	Yes	Yes	Yes	Yes	Yes	No	Yes	Low risk of bias	Humans
Staples, 2014	No	Yes	Yes	Yes	Yes	Yes	Yes	Yes	No	Yes	Low risk of bias	Humans
Staples, 2014	No	Yes	Yes	Not applicable	Yes	Yes	Yes	Yes	No	Yes	Low risk of bias	Individual Mosquitoes
Staples, 2014	No	Yes	Yes	Not applicable	Yes	Yes	Yes	Yes	No	Yes	Low risk of bias	Non-human primates
Sutherland, 2011	No	Yes	No	Yes	Yes	No	Yes	Yes	Yes	Yes	Low risk of bias	Humans
Sutherland, 2011	No	Yes	No	Yes	Yes	No	Yes	Yes	Yes	Yes	Low risk of bias	Humans
Ushijima, 2021	No	Yes	No	Yes	Yes	No	Yes	Yes	Yes	Yes	Low risk of bias	Humans
Wamala, 2012	No	Yes	No	Yes	Yes	No	Yes	Yes	No	Yes	Moderate risk of bias	Humans
Wamala, 2012	No	Yes	No	Yes	Yes	No	Yes	Yes	No	Yes	Moderate risk of bias	Humans
Wastika, 2019	No	Yes	No	Not applicable	Yes	No	Yes	Yes	Unclear	Yes	Moderate risk of bias	Non-human primates
Wastika, 2019	No	Yes	No	Not applicable	Yes	No	Yes	Yes	Unclear	Yes	Moderate risk of bias	Non-human primates
Wastika, 2019	No	Yes	No	Not applicable	Yes	No	Yes	Yes	Unclear	Yes	Moderate risk of bias	Non-human primates
Wastika, 2019	No	Yes	No	Not applicable	Yes	No	Yes	Yes	Unclear	Yes	Moderate risk of bias	Non-human primates
Willcox, 2018	No	Yes	Yes	Yes	Yes	No	No	Yes	Unclear	Yes	Moderate risk of bias	Humans
Yaro, 2010	No	Yes	No	Yes	Yes	No	Yes	Yes	Yes	Yes	Low risk of bias	Humans
Yaro, 2010	No	Yes	No	Yes	Yes	No	Yes	Yes	Yes	Yes	Low risk of bias	Humans

Reference

1. Agwu EJ, Igbinosa IB, Isaac C. Entomological assessment of yellow fever-epidemic risk indices in Benue State, Nigeria, 2010-2011. Acta tropica. 2016;161:18-25. Epub 2016/05/18. doi: 10.1016/j.actatropica.2016.05.005. PubMed PMID: 27189925.
2. Ahmed SS, Soghaier MA, Mohammed S, Khogali HS, Osman MM, Abdalla AM. Concomitant outbreaks of yellow fever and hepatitis E virus in Darfur States, Sudan, 2012. Journal of infection in developing countries. 2016;10(1):24-9. Epub 2016/02/02. doi: 10.3855/jidc.6342. PubMed PMID: 26829534.
3. Akelew Y, Pareyn M, Lemma M, Negash M, Bewket G, Derbew A, et al. Aetiologies of acute undifferentiated febrile illness at the emergency ward of the University of Gondar Hospital, Ethiopia. Tropical medicine & international health : TM & IH. 2022. Epub 2022/01/15. doi: 10.1111/tmi.13721. PubMed PMID: 35029010.
4. Akoua-Koffi C, Akran V, kata Faye O, Grandadam M, Ekaza E, Kouassi KS, et al. Yellow fever and dengue fever serotype 3 viruses cocirculation in Côte d'Ivoire in 2008. African Journal of Pathology and Microbiology. 2014.
5. Akoua-Koffi C, Diarrassouba S, Benie VB, Ngbichi JM, Bozoua T, Bosson A, et al. Inquiry into a fatal case of yellow fever in Cote d'lvoire in 1999. Bulletin De La Societe De Pathologie Exotique. 2001;94(3):227-30. PubMed PMID: CCC:000171401500001.
6. Akoua-Koffi C, Ekra KD, Kone AB, Dagnan NS, Akran V, Kouadio KL, et al. [Detection and management of the yellow fever epidemic in the Ivory Coast, 2001]. Medecine tropicale : revue du Corps de sante colonial. 2002;62(3):305-9. Epub 2002/09/25. PubMed PMID: 12244930.
7. Alhakimi HA, Mohamed OG, Khogaly HSE, Arafa KAO, Ahmed WA. Epidemiological, Clinical and Entomological Characteristics of Yellow Fever Outbreak in Darfur 2012. AIMS public health. 2015;2(1):132-41. Epub 2015/03/25. doi: 10.3934/publichealth.2015.1.132. PubMed PMID: 29546100; PubMed Central PMCID: PMCPmc5690374.
8. Asebe G, Michlmayr D, Mamo G, Abegaz WE, Endale A, Medhin G, et al. Seroprevalence of Yellow fever, Chikungunya, and Zika virus at a community level in the Gambella Region, South West Ethiopia. PloS one. 2021;16(7):e0253953. Epub 2021/07/09. doi: 10.1371/journal.pone.0253953. PubMed PMID: 34237098; PubMed Central PMCID: PMCPMC8266044.
9. Baba MM, Yahaya KM, Ezra EM, Adamu M, Kulloma BM, Ikusemoran M, et al. Assessment of immunity against Yellow Fever virus infections in northeastern Nigeria using three serological assays. Journal of medical virology. 2021;93(8):4856-64. Epub 2021/03/31. doi: 10.1002/jmv.26978. PubMed PMID: 33783842.
10. Babaniyi OA, Mwaba P, Mulenga D, Monze M, Songolo P, Mazaba-Liwewe ML, et al. Risk assessment for yellow Fever in Western and north-Western provinces of zambia. Journal of global infectious diseases. 2015;7(1):11-7. Epub 2015/02/28. doi: 10.4103/0974-777x.150884. PubMed PMID: 25722614; PubMed Central PMCID: PMCPmc4338443.
11. Caux C, Etxeberrria I, Teijeira A, Marabelle A, Ajogbasile FV, Oguzie JU, et al. Real-time Metagenomic Analysis of Undiagnosed Fever Cases Unveils a Yellow Fever Outbreak in Edo State, Nigeria. Journal for immunotherapy of cancer. 2020;10(1):3180. Epub 2020/02/28. doi: 10.1136/jitc-2019-000443
10.1038/s41598-020-59880-w. PubMed PMID: 32081931; PubMed Central PMCID: PMCPmc7057427
Pmc7035389.

12. Chepkorir E. Serological evidence of Flavivirus circulation in human populations in Northern Kenya: an assessment of disease risk 2016-2017. Pharmaceutical research. 2019;16(1):65. Epub 2019/05/19. doi: 10.1007/s11095-019-2629-0
10.1186/s12985-019-1176-y. PubMed PMID: 31101058; PubMed Central PMCID: PMCPmc6668022.
13. Diagne MM, Ndione MHD, Gaye A, Barry MA, Diallo D, Diallo A, et al. Yellow Fever Outbreak in Eastern Senegal, 2020-2021. Viruses. 2021;13(8). Epub 2021/08/29. doi: 10.3390/v13081475. PubMed PMID: 34452343; PubMed Central PMCID: PMCPMC8402698.
14. Diallo BI, Bah MB, Yattara F, Keleba RG, MacDonald PDM, Dieng I. Mobile Laboratory Reveals the Circulation of Dengue Virus Serotype I of Asian Origin in Medina Gounass (Guediawaye), Senegal. PloS one. 2020;10(6). Epub 2020/06/26. doi: 10.1371/journal.pone.0234796
10.3390/diagnostics10060408. PubMed PMID: 32560073; PubMed Central PMCID: PMCPmc7316275.
15. Diallo D, Fall G, Diagne CT, Gaye A, Ba Y, Dia I, et al. Concurrent amplification of Zika, chikungunya, and yellow fever virus in a sylvatic focus of arboviruses in Southeastern Senegal, 2015. BMC microbiology. 2020;20(1):181. Epub 2020/07/14. doi: 10.1016/j.vaccine.2020.06.079
10.1186/s12866-020-01866-9. PubMed PMID: 32590939; PubMed Central PMCID: PMCPmc7318437.
16. Diallo D, Sall AA, Diagne CT, Faye O, Hanley KA, Buenemann M, et al. Patterns of a sylvatic yellow fever virus amplification in southeastern Senegal, 2010. The American journal of tropical medicine and hygiene. 2014;90(6):1003-13. Epub 2014/03/13. doi: 10.4269/ajtmh.13-0404. PubMed PMID: 24615140; PubMed Central PMCID: PMCPmc4047721.
17. Ekenna O, Chikwem JO, Mohammed I, Durojaiye SO. Epidemic yellow fever in Borno State of Nigeria: characterisation of hospitalised patients. West African journal of medicine. 2010;29(2):91-7. Epub 2010/06/15. PubMed PMID: 20544633.
18. Endale A. Community-based sero-prevalence of chikungunya and yellow fever in the South Omo Valley of Southern Ethiopia. BMJ open. 2020;14(9):e0008549. Epub 2020/09/05. doi: 10.1136/bmjopen-2020-037903
10.1371/journal.pntd.0008549. PubMed PMID: 32881913; PubMed Central PMCID: PMCPmc7473631.
19. Farnon EC, Gould LH, Griffith KS, Osman MS, Kholy AE, Brair ME, et al. Household-based sero-epidemiologic survey after a yellow fever epidemic, Sudan, 2005. The American journal of tropical medicine and hygiene. 2010;82(6):1146-52. Epub 2010/06/04. doi: 10.4269/ajtmh.2010.09-0105. PubMed PMID: 20519615; PubMed Central PMCID: PMCPmc2877426.
20. Faye O, Diallo M, Dia I, Ba Y, Faye O, Mondo M, et al. [Integrated approach to yellow fever surveillance: pilot study in Senegal in 2003-2004]. Bulletin de la Societe de pathologie exotique (1990). 2007;100(3):187-92. Epub 2007/09/11. PubMed PMID: 17824313.
21. Fokam EB, Levai LD, Guzman H, Amelia PA, Titanji VP, Tesh RB, et al. Silent circulation of arboviruses in Cameroon. East African medical journal. 2010;87(6):262-8. Epub 2010/06/01. doi: 10.4314/eamj.v87i6.63085. PubMed PMID: 23057269.
22. Gould LH, Osman MS, Farnon EC, Griffith KS, Godsey MS, Karch S, et al. An outbreak of yellow fever with concurrent chikungunya virus transmission in South Kordofan, Sudan, 2005. Transactions of the Royal Society of Tropical Medicine and Hygiene. 2008;102(12):1247-54. Epub 2008/05/27. doi: 10.1016/j.trstmh.2008.04.014. PubMed PMID: 18502458.

23. Ingelbeen B, Weregemere NA, Noel H, Tshapenda GP, Mossoko M, Nsio J, et al. Urban yellow fever outbreak-Democratic Republic of the Congo, 2016: Towards more rapid case detection. PLoS neglected tropical diseases. 2018;12(12):e0007029. Epub 2018/12/12. doi: 10.1371/journal.pntd.0007029. PubMed PMID: 30532188; PubMed Central PMCID: PMCPMC6300298.
24. Inziani M, Adungo F, Awando J, Kihoro R, Inoue S, Morita K, et al. Seroprevalence of yellow fever, dengue, West Nile and chikungunya viruses in children in Teso South Sub-County, Western Kenya. International journal of infectious diseases : IJID : official publication of the International Society for Infectious Diseases. 2020;91:104-10. Epub 2019/11/13. doi: 10.4102/phcfm.v11i1.2063
10.1016/j.ijid.2019.11.004. PubMed PMID: 31712089.
25. Jentes ES, Robinson J, Johnson BW, Conde I, Sakouvougui Y, Iverson J, et al. Acute arboviral infections in Guinea, West Africa, 2006. The American journal of tropical medicine and hygiene. 2010;83(2):388-94. Epub 2010/08/05. doi: 10.4269/ajtmh.2010.09-0688. PubMed PMID: 20682888; PubMed Central PMCID: PMCPmc2911191.
26. Kading RC, Borland EM, Cranfield M, Powers AM. Prevalence of antibodies to alphaviruses and flaviviruses in free-ranging game animals and nonhuman primates in the greater Congo basin. Journal of wildlife diseases. 2013;49(3):587-99. Epub 2013/06/20. doi: 10.7589/2012-08-212. PubMed PMID: 23778608.
27. Kading RC, Kityo RM, Mossel EC, Borland EM, Nakayiki T, Nalikka B, et al. Neutralizing antibodies against flaviviruses, Babanki virus, and Rift Valley fever virus in Ugandan bats. PLoS neglected tropical diseases. 2018;8(1):1439215. Epub 2018/03/16. doi: 10.1371/journal.pntd.0006284
10.1080/20008686.2018.1439215. PubMed PMID: 29511459; PubMed Central PMCID: PMCPmc5854243.
28. Kayiwa JT, Nankya AM, Ataliba IJ, Mossel EC, Crabtree MB, Lutwama JJ. Confirmation of Zika virus infection through hospital-based sentinel surveillance of acute febrile illness in Uganda, 2014-2017. Journal of General Virology. 2018;99(9):1248-52. PubMed PMID: CCC:000443388700010.
29. Konongoi L, Ofula V, Nyunja A, Owaka S, Koka H, Makio A, et al. Detection of dengue virus serotypes 1, 2 and 3 in selected regions of Kenya: 2011-2014. Virology journal. 2016;13(1):182. Epub 2016/11/07. doi: 10.1186/s12985-016-0641-0. PubMed PMID: 27814732; PubMed Central PMCID: PMCPmc5097412.
30. Kuniholm MH, Wolfe ND, Huang CY, Mpoudi-Ngole E, Tamoufe U, LeBreton M, et al. Seroprevalence and distribution of Flaviviridae, Togaviridae, and Bunyaviridae arboviral infections in rural Cameroonian adults. The American journal of tropical medicine and hygiene. 2006;74(6):1078-83. Epub 2006/06/09. PubMed PMID: 16760524.
31. Kwagonza L, Masiira B, Kyobe-Bosa H, Kadobera D, Atuheire EB, Lubwama B, et al. Outbreak of yellow fever in central and southwestern Uganda, February-may 2016. BMC infectious diseases. 2018;18(1):548. Epub 2018/11/08. doi: 10.3390/diseases6040099
10.1186/s12879-018-3440-y. PubMed PMID: 30390621; PubMed Central PMCID: PMCPmc6215607.
32. Kwallah A, Inoue S, Thairu-Muigai AW, Kuttoh N, Morita K, Mwau M. Seroprevalence of yellow fever virus in selected health facilities in Western Kenya from 2010 to 2012. Japanese journal of infectious diseases. 2015;68(3):230-4. Epub 2015/02/13. doi: 10.7883/yoken.JJID.2014.288. PubMed PMID: 25672346.

33. Lilay A, Asamene N, Bekele A, Mengesha M, Wendabeku M, Tareke I, et al. Reemergence of yellow fever in Ethiopia after 50 years, 2013: epidemiological and entomological investigations. BMC infectious diseases. 2017;17(1):343. Epub 2017/05/17. doi: 10.1007/s13149-017-0557-y
10.1186/s12879-017-2435-4. PubMed PMID: 28506254; PubMed Central PMCID: PMCPmc5432991.
34. Mease LE, Coldren RL, Musila LA, Prosser T, Ogolla F, Ofula VO, et al. Seroprevalence and distribution of arboviral infections among rural Kenyan adults: a cross-sectional study. Virology journal. 2011;8:371. Epub 2011/07/29. doi: 10.1186/1743-422x-8-371. PubMed PMID: 21794131; PubMed Central PMCID: PMCPmc3161961.
35. Mengesha Tsegaye M, Beyene B, Ayele W, Abebe A, Tareke I, Sall A, et al. Sero-prevalence of yellow fever and related Flavi viruses in Ethiopia: a public health perspective. BMC public health. 2018;18(1):1011. Epub 2018/08/21. doi: 10.1080/17441692.2018.1512144
10.1186/s12889-018-5726-9. PubMed PMID: 30107830; PubMed Central PMCID: PMCPmc6092792.
36. Mohamed N, Magzoub M, Mohamed REH, Aleanizy FS, Alqahtani FY, Nour BYM, et al. Prevalence and identification of arthropod-transmitted viruses in Kassala state, Eastern Sudan. The Libyan journal of medicine. 2019;14(1):1564511-. PubMed PMID: MEDLINE:30716013.
37. Mulchandani R, Massebo F, Bocho F, Jeffries CL, Walker T, Messenger LA. A community-level investigation following a yellow fever virus outbreak in South Omo Zone, South-West Ethiopia. PeerJ. 2019;7:e6466. Epub 2019/02/28. doi: 10.7717/peerj.6466. PubMed PMID: 30809451; PubMed Central PMCID: PMCPMC6387579.
38. Nakounne E, Selekon B, Morvan J. Microbiological surveillance: viral haemorrhagic fevers in the Central African Republic; updated serological data for human beings. Bulletin De La Societe De Pathologie Exotique. 2001;93(5):340-7. PubMed PMID: CCC:000170522100010.
39. Ngoagouni C, Kamgang B, Manirakiza A, Nangouma A, Paupy C, Nakoune E, et al. Entomological profile of yellow fever epidemics in the Central African Republic, 2006-2010. Parasites & vectors. 2012;5:175. Epub 2012/08/18. doi: 10.1186/1756-3305-5-175. PubMed PMID: 22897918; PubMed Central PMCID: PMCPmc3436863.
40. Nwachukwu WE, Yusuff H, Nwangwu U, Okon A, Ogunniyi A, Imuetinyan-Clement J, et al. The response to re-emergence of yellow fever in Nigeria, 2017. International journal of infectious diseases : IJID : official publication of the International Society for Infectious Diseases. 2020;92:189-96. Epub 2020/01/15. doi: 10.1016/j.ijid.2019.12.034. PubMed PMID: 31935537.
41. Onyango CO, Ofula VO, Sang RC, Konongoi SL, Sow A, De Cock KM, et al. Yellow fever outbreak, Imatong, southern Sudan. Emerging infectious diseases. 2004;10(6):1063-8. Epub 2004/06/23. doi: 10.3201/eid1006.030738. PubMed PMID: 15207058; PubMed Central PMCID: PMCPmc3323161.
42. Otshudiema JO, Ndakala NG, Mawanda EK, Tshapenda GP, Kimfuta JM, Nsibu LN, et al. Yellow Fever Outbreak - Kongo Central Province, Democratic Republic of the Congo, August 2016. MMWR Morbidity and mortality weekly report. 2017;66(12):335-8. Epub 2017/04/04 2017/03/31. doi: 10.1016/j.vaccine.2017.03.032
10.15585/mmwr.mm6612a5. PubMed PMID: 28358796; PubMed Central PMCID: PMCPmc5657954.
43. Proesmans S, Katshongo F, Milambu J, Fungula B, Muhindo Mavoko H, Ahuka-Mundeke S, et al. Dengue and chikungunya among outpatients with acute undifferentiated fever

in Kinshasa, Democratic Republic of Congo: A cross-sectional study. 2019;13(9):e0007047. Epub 2019/09/24. doi: 10.1016/s1473-3099(19)30323-8
10.1371/journal.pntd.0007047. PubMed PMID: 31487279; PubMed Central PMCID: PMCPmc6892259.
44. Rachas A, Nakouné E, Bouscaillou J, Paireau J, Selekon B, Senekian D, et al. Timeliness of yellow fever surveillance, Central African Republic. Emerging infectious diseases. 2014;20(6):1004-8. Epub 2014/05/27. doi: 10.3201/eid2006.130671. PubMed PMID: 24857597; PubMed Central PMCID: PMCPmc4036780.
45. Rugarabamu S, Mwanyika GO, Rumisha SF, Sindato C, Lim HY, Misinzo G, et al. Seroprevalence and associated risk factors of selected zoonotic viral hemorrhagic fevers in Tanzania. International journal of infectious diseases : IJID : official publication of the International Society for Infectious Diseases. 2021;109:174-81. Epub 2021/07/10. doi: 10.1016/j.ijid.2021.07.006. PubMed PMID: 34242761.
46. Schoepp RJ, Rossi CA, Khan SH, Goba A, Fair JN. Undiagnosed acute viral febrile illnesses, Sierra Leone. Emerging infectious diseases. 2014;20(7):1176-82. Epub 2014/06/25. doi: 10.3201/eid2007.131265. PubMed PMID: 24959946; PubMed Central PMCID: PMCPmc4073864.
47. Simo Tchetgna H, Sem Ouilibona R, Nkili-Meyong AA, Caron M, Labouba I, Selekon B, et al. Viral Exploration of Negative Acute Febrile Cases Observed during Chikungunya Outbreaks in Gabon. Intervirology. 2018;61(4):174-84. Epub 2019/01/10. doi: 10.1159/000495136. PubMed PMID: 30625488.
48. Sow A, Loucoubar C, Diallo D, Faye O, Ndiaye Y, Senghor CS, et al. Concurrent malaria and arbovirus infections in Kedougou, southeastern Senegal. Malaria journal. 2016;15:47. Epub 2016/01/30. doi: 10.1186/s12936-016-1100-5. PubMed PMID: 26821709; PubMed Central PMCID: PMCPmc4730666.
49. Staples JE, Diallo M, Janusz KB, Manengu C, Lewis RF, Perea W, et al. Yellow fever risk assessment in the Central African Republic. Transactions of the Royal Society of Tropical Medicine and Hygiene. 2014;108(10):608-15. Epub 2014/06/21. doi: 10.1093/trstmh/tru086. PubMed PMID: 24947520; PubMed Central PMCID: PMCPmc4653062.
50. Sutherland LJ, Cash AA, Huang Y-JS, Sang RC, Malhotra I, Moormann AM, et al. Short Report: Serologic Evidence of Arboviral Infections among Humans in Kenya. American Journal of Tropical Medicine and Hygiene. 2011;85(1):158-61. PubMed PMID: CCC:000292433200028.
51. Ushijima Y, Abe H, Nguema Ondo G, Bikangui R, Massinga Loembé M, Zadeh VR, et al. Surveillance of the major pathogenic arboviruses of public health concern in Gabon, Central Africa: increased risk of West Nile virus and dengue virus infections. BMC infectious diseases. 2021;21(1):265. Epub 2021/03/19. doi: 10.1186/s12879-021-05960-9. PubMed PMID: 33731022; PubMed Central PMCID: PMCPMC7966894.
52. Wamala JF, Malimbo M, Okot CL, Atai-Omoruto AD, Tenywa E, Miller JR, et al. Epidemiological and laboratory characterization of a yellow fever outbreak in northern Uganda, October 2010-January 2011. International journal of infectious diseases : IJID : official publication of the International Society for Infectious Diseases. 2012;16(7):e536-42. Epub 2012/05/12. doi: 10.1016/j.ijid.2012.03.004. PubMed PMID: 22575876.
53. Wastika CE, Sasaki M, Yoshii K, Anindita PD, Hang'ombe BM, Mweene AS, et al. Serological evidence of Zika virus infection in non-human primates in Zambia. 2019;164(8):2165-70. doi: 10.1007/s00705-019-04302-0. PubMed PMID: 31154511.
54. Willcox AC, Collins MH, Jadi R, Keeler C, Parr JB, Mumba D, et al. Seroepidemiology of Dengue, Zika, and Yellow Fever Viruses among Children in the Democratic Republic of the

Congo. The American journal of tropical medicine and hygiene. 2018;99(3):756-63. Epub 2018/07/13
2018/07/11. doi: 10.1186/s12889-018-5762-5
10.4269/ajtmh.18-0156. PubMed PMID: 29988000; PubMed Central PMCID: PMCPmc6169194.
55. Yaro S, Zango A, Rouamba J, Diabaté A, Dabiré R, Kambiré C, et al. [Epidemiological situation of yellow fever in Burkina Faso from 2003 to 2008]. Bulletin de la Societe de pathologie exotique (1990). 2010;103(1):44-7. Epub 2010/01/27. doi: 10.1007/s13149-009-0032-5. PubMed PMID: 20101488.
