# Supplementary material for: Epidemiology of yellow fever virus in humans, arthropods, and non-human primates in sub-Saharan Africa: A systematic review and meta-analysis
Source: PLoS Negl Trop Dis. 2022 Jul 22;16(7):e0010610. doi: 10.1371/journal.pntd.0010610 (PMC9307179; doi:10.1371/journal.pntd.0010610)
Supplement: S6 Table — (PDF) [file pntd.0010610.s006.pdf]

S6 Table. Characteristics of included studies

| **Characteristics** | **Overall (151)** | **CFR (4)** | **Human prevalence (71)** | **Mosquitoes (65)** | **Non human primates (7)** | **Other animal species (4)** |
|---|---|---|---|---|---|---|
| **Year of publication; range** | 2001-2022 | 2012-2020 | 2001-2022 | 2007-2021 | 2013-2019 | 2013-2018 |
| **Period of inclusion of participants; range** | 1990-2021 | 2010-2016 | 1990-2021 | 2003-2021 | 1991-2010 | 1991-2013 |
| **Study Design** | | | | | | |
| Cross sectional | 135 (88.7) | 1 (25.0) | 58 (81.7) | 65 (100) | 7 (100) | 4 (100) |
| Outbreak | 16 (11.3) | 3 (75.0) | 13 (18.3) | | | |
| **Sampling** | | | | | | |
| Non probabilistic | 137 (90.7) | 4 (100) | 59 (83.1) | 64 (98.5) | 6 (85.7) | 4 (100) |
| Probabilistic | 14 (9.3) | | 12 (16.9) | 1 (1.5) | 1 (14.3) | |
| **Setting** | | | | | | |
| Community-based | 115 (76.2) | 2 (50.0) | 37 (52.1) | 65 (100) | 7 (100) | 4 (100) |
| Hospital-based | 27 (17.9) | 1 (25.0) | 26 (36.6) | | | |
| Hospital/community based | 9 (5.3) | 1 (25.0) | 8 (9.9) | | | |
| **Hospitalization** | | | | | | |
| Ambulatory | 16 (11.9) | 1 (25.0) | 15 (21.1) | | | |
| Hospitalized | 11 (7.3) | | 11 (15.5) | | | |
| Hospitalized/ambulatory | 2 (1.3) | 1 (25.0) | 1 (1.4) | | | |
| Not applicable | 113 (73.5) | 2 (50.0) | 35 (49.3) | 65 (100) | 7 (100) | 4 (100) |
| Unclear/Not reported | 9 (6.0) | | 9 (12.7) | | | |
| **Number of sites** | | | | | | |
| Monocenter | 15 (9.9) | | 10 (14.1) | 1 (1.5) | 4 (57.1) | |
| Multicenter | 136 (90.1) | 4 (100) | 61 (85.9) | 64 (98.5) | 3 (42.9) | 4 (100) |
| **Timing of samples collection** | | | | | | |
| Prospetively | 134 (88.7) | 4 (100) | 63 (88.7) | 65 (100) | 1 (14.3) | 1 (25.0) |
| Retrospectively | 17 (11.3) | | 8 (11.3) | | 6 (85.7) | 3 (75.0) |
| **Countries** | | | | | | |
| Senegal | 43 (28.5) | | 6 (8.5) | 37 (56.9) | | |
| Central African Republic | 33 (21.9) | | 7 (9.9) | 25 (38.5) | 1 (14.3) | |
| Côte d'Ivoire | 11 (7.3) | | 11 (15.5) | | | |
| Democratic Republic of the Congo | 10 (6.6) | 1 (25.0) | 6 (8.5) | | 3 (75.0) | 3 (75.0) |
| Kenya | 9 (6.0) | | 8 (11.3) | 1 (1.5) | | |
| Sudan | 7 (4.6) | 1 (25.0) | 6 (8.5) | | | |
| Uganda | 7 (4.6) | 1 (25.0) | 4 (5.6) | 1 (1.5) | 1 (25.0) | 1 (25.0) |
| Ethiopia | 6 (4.0) | | 5 (7.0) | 1 (1.5) | | |

| | | | | | | |
|---|---|---|---|---|---|---|
| Zambia | 6 (4.0) | | 2 (2.8) | | 4 (57.1) | |
| Nigeria | 5 (3.3) | 1 (25.0) | 4 (5.6) | | | |
| Cameroon | 3 (2.0) | | 3 (4.2) | | | |
| Gabon | 3 (2.0) | | 2 (2.8) | | 1 (14.3) | |
| Burkina Faso | 2 (1.3) | | 2 (2.8) | | | |
| Tanzania | 2 (1.3) | | 2 (2.8) | | | |
| Guinea | 1 (0.7) | | 1 (1.4) | | | |
| Rwanda | 1 (0.7) | | | | 1 (14.3) | |
| Sierra Leone | 1 (0.7) | | 1 (1.4) | | | |
| South Sudan | 1 (0.7) | | 1 (1.4) | | | |
| **UNSD Region** | | | | | | |
| West Africa | 63 (41.7) | 1 (25.0) | 25 (35.2) | 37 (56.9) | | |
| Central Africa | 49 (32.5) | 1 (25.0) | 18 (25.4) | 25 (38.5) | 2 (28.6) | 3 (75.0) |
| Eastern Africa | 32 (21.2) | 1 (25.0) | 22 (31.0) | 3 (4.6) | 5 (71.4) | 1 (25.0) |
| Northern Africa | 7 (4.6) | 1 (25.0) | 6 (8.5) | | | |
| **Country income level** | | | | | | |
| Low-income economies | 69 (45.7) | 3 (75.0) | 33 (46.5) | 27 (41.5) | 2 (28.6) | 4 (100) |
| Lower-middle income economies | 79 (52.3) | 1 (25.0) | 36 (50.7) | 38 (58.5) | 4 (57.1) | |
| Upper-middle-income economies | 3 (2.0) | | 2 (2.8) | | 1 (14.3) | |
| **Age range** | | | | | | |
| Adults | 9 (6.0) | 1 (25.0) | 8 (11.3) | | | |
| All ages | 31 (20.5) | 2 (50.0) | 29 (40.9) | | | |
| Children | 3 (2.0) | | 3 (4.2) | | | |
| Not applicable | 72 (49.0) | | | 65 (100) | 7 (100) | 4 (100) |
| Unclear/Not reported | 34 (22.5) | 1 (25.0) | 9 (12.7) | | | |
| **YFV vaccine** | | | | | | |
| No | 18 (11.9) | 1 (25.0) | 17 (23.9) | | | |
| Yes/No | 10 (6.6) | | 10 (14.1) | | | |
| Not applicable | 75 (49.7) | | | 65 (100) | 7 (100) | 4 (100) |
| Unclear/Not reported | 47 (31.1) | 1 (25.0) | 9 (12.7) | | | |
| **Species** | | | | | | |
| Humans | 75 (49.7) | 4 (100) | 71 (100) | | | |
| Individual Mosquitoes | 62 (41.1) | | | 62 (95.4) | | |
| Pooled mosquitoes | 3 (2.0) | | | 3 (4.6) | | |
| Non-human primates | 7 (4.6) | | | | 7 (100) | |
| Other animal species | 4 (2.7) | | | | | 4 (100) |
| **Humans** | | | | | | |

| | | | | | | |
|---|---|---|---|---|---|---|
| Apparently healthy individuals | 23 (15.2) | | 23 (32.4) | | | |
| Febrile patients | 14 (9.3) | | 14 (19.7) | | | |
| General population | 2 (1.3) | | 2 (2.8) | | | |
| Mixed human categories | 4 (2.7) | | 4 (5.6) | | | |
| Positive among YFV suspected cases | 4 (2.7) | 4 (100) | | | | |
| Pregnant women | 1 (0.7) | | 1 (1.4) | | | |
| YFV positive case contact | 4 (2.7) | | 4 (5.6) | | | |
| YFV suspected cases | 23 (14.6) | | 23 (31.0) | | | |
| **Individual mosquitoes** | | | | | | |
| Aedes aegypti | 1 (0.7) | | | 1 (1.5) | | |
| Aedes Aegypti | 3 (2.0) | | | 1 (1.5) | | |
| Aedes aegypti formosus | 1 (0.7) | | | 1 (1.5) | | |
| Aedes Aegypti, Aedes africanus, Mansonia africana | 1 (0.7) | | | 1 (1.5) | | |
| Aedes africanus | 2 (1.3) | | | 2 (3.1) | | |
| Aedes argenteopunctatus | 2 (1.3) | | | 2 (3.1) | | |
| Aedes centropunctatus | 1 (0.7) | | | 1 (1.5) | | |
| Aedes circumluteolus | 1 (0.7) | | | 1 (1.5) | | |
| Aedes cumminsi | 1 (0.7) | | | 1 (1.5) | | |
| Aedes dalzieli | 1 (0.7) | | | 1 (1.5) | | |
| Aedes fowleri | 1 (0.7) | | | 1 (1.5) | | |
| Aedes furcifer | 1 (0.7) | | | 1 (1.5) | | |
| Aedes hirsutus | 1 (0.7) | | | 1 (1.5) | | |
| Aedes luteocephalus | 1 (0.7) | | | 1 (1.5) | | |
| Aedes Luteocephalus | 1 (0.7) | | | 1 (1.5) | | |
| Aedes metallicus | 1 (0.7) | | | 1 (1.5) | | |
| Aedes minutus | 1 (0.7) | | | 1 (1.5) | | |
| Aedes opok | 1 (0.7) | | | 1 (1.5) | | |
| Aedes simpsoni | 1 (0.7) | | | 1 (1.5) | | |
| Aedes Simpsoni | 1 (0.7) | | | 1 (1.5) | | |
| Aedes taylori | 2 (1.3) | | | 2 (3.1) | | |
| Aedes unilineatus | 1 (0.7) | | | 1 (1.5) | | |
| Aedes vittatus | 2 (1.3) | | | 2 (3.1) | | |
| Anopheles bambusae | 1 (0.7) | | | 1 (1.5) | | |
| Anopheles coustani | 2 (1.3) | | | 2 (3.1) | | |
| Anopheles flavicosta | 1 (0.7) | | | 1 (1.5) | | |
| Anopheles funestus | 2 (1.3) | | | 2 (3.1) | | |
| Anopheles gambiae | 1 (0.7) | | | 1 (1.5) | | |

| | | | | | | |
|---|---|---|---|---|---|---|
| Anopheles gambiae s.l. | 1 (0.7) | | | 1 (1.5) | | |
| Anopheles implexus | 1 (0.7) | | | 1 (1.5) | | |
| Anopheles paludis | 1 (0.7) | | | 1 (1.5) | | |
| Anopheles pharoensis | 1 (0.7) | | | 1 (1.5) | | |
| Anopheles rufipes | 1 (0.7) | | | 1 (1.5) | | |
| Anopheles ziemanni | 1 (0.7) | | | 1 (1.5) | | |
| Culex annulioris | 2 (1.3) | | | 2 (3.1) | | |
| Culex antennatus | 1 (0.7) | | | 1 (1.5) | | |
| Culex bitaeniorhynchus | 1 (0.7) | | | 1 (1.5) | | |
| Culex cinereus | 1 (0.7) | | | 1 (1.5) | | |
| Culex neavei | 1 (0.7) | | | 1 (1.5) | | |
| Culex perfuscus | 2 (1.3) | | | 2 (3.1) | | |
| Culex poicilipes | 2 (1.3) | | | 2 (3.1) | | |
| Culex quinquefasciatus | 2 (1.3) | | | 2 (3.1) | | |
| Culex tigripes | 1 (0.7) | | | 1 (1.5) | | |
| Culex sp. | 1 (0.7) | | | 1 (1.5) | | |
| Eretmapodites chrysogaster | 1 (0.7) | | | 1 (1.5) | | |
| Eretmapodites inornatus | 1 (0.7) | | | 1 (1.5) | | |
| Mansonia africana | 2 (1.3) | | | 2 (3.1) | | |
| Mansonia uniformis | 2 (1.3) | | | 2 (3.1) | | |
| **Mosquitoes gender** | | | | | | |
| Aedes | 28 (18.5) | | | 28 (43.1) | | |
| Anopheles | 13 (8.6) | | | 13 (20.0) | | |
| Culex | 14 (9.3) | | | 14 (21.5) | | |
| Mansonia | 4 (2.7) | | | 4 (6.2) | | |
| Eretmapodites | 2 (1.3) | | | 2 (3.1) | | |
| Multiple species | 1 (0.7) | | | 1 (1.5) | | |
| **Pooled mosquitoes** | | | | | | |
| Aedes aegypti, Aedes furcifer | 1 (0.7) | | | 1 (1.5) | | |
| Aedes africanus, Aedes centropunctatus, Aedes dalzieli, Aedes furcifer–taylori, Aedes Luteocephalus, Aedes mcintoshi, Aedes taylori, Aedes vittatus, Anopheles funestus | 1 (0.7) | | | 1 (1.5) | | |
| Aedes Furcifer; Aedes Luteocephalus; Aedes Taylori; Aedes Vittatus; Aedes africanus | 1 (0.7) | | | 1 (1.5) | | |
| **Pooled mosquitoes family** | | | | | | |
| Culicidae | 3 (2.0) | | | 3 (4.6) | | |
| **Non-human primates** | | | | | | |

| | | | | | | |
|---|---|---|---|---|---|---|
| Baboons | 2 (1.3) | | | | 2 (28.6) | |
| Chlorocebus spp, Cercopitheque spp, Cynocephalus spp, and Erythrocebus spp | 1 (0.7) | | | | 1 (14.3) | |
| Gorilla beringei beringei | 1 (0.7) | | | | 1 (14.3) | |
| Mandrills | 1 (0.7) | | | | 1 (14.3) | |
| Monkeys | 2 (1.3) | | | | 2 (28.6) | |
| **Non-human primates Family** | | | | | | |
| Hominidae | 1 (0.7) | | | | 1 (14.3) | |
| Cercopithecidae | 6 (4.0) | | | | 6 (85.7) | |
| **Other animal species** | | | | | | |
| Bats | 1 (0.7) | | | | | 1 (25.0) |
| Buffalo | 1 (0.7) | | | | | 1 (25.0) |
| Duike | 1 (0.7) | | | | | 1 (25.0) |
| Elephant | 1 (0.7) | | | | | 1 (25.0) |
| **Other animal species order** | | | | | | |
| Artiodactyla | 2 (1.3) | | | | | 2 (50.0) |
| Chiroptera | 1 (0.7) | | | | | 1 (25.0) |
| Proboscidea | 1 (0.7) | | | | | 1 (25.0) |
| **Detection assay** | | | | | | |
| Indirect ELISA | 38 (25.2) | 1 (25.0) | 36 (50.7) | | 1 (14.3) | 1 (14.3) |
| Real Time RT-PCR | 37 (24.5) | | 7 (9.9) | 28 (43.1) | 2 (28.6) | 2 (28.6) |
| Culture | 35 (23.2) | | 2 (2.8) | 33 (50.8) | | |
| Plaque reduction neutralization test (PRNT) | 12 (8.0) | | 4 (5.6) | | 4 (57.1) | 4 (57.1) |
| Classical RT-PCR | 7 (4.6) | | 3 (4.2) | 4 (6.2) | | |
| Indirect ELISA, Plaque reduction neutralization test (PRNT) | 6 (4.0) | | 6 (8.5) | | | |
| Indirect ELISA, Real Time RT-PCR | 4 (2.7) | 1 (25.0) | 3 (4.2) | | | |
| Indirect ELISA, Plaque reduction neutralization test (PRNT), Classical RT-PCR | 3 (2.0) | 1 (25.0) | 2 (2.8) | | | |
| Enzyme immunoassay | 2 (1.3) | | 2 (2.8) | | | |
| Indirect immunofluorescence assay | 2 (1.3) | | 2 (2.8) | | | |
| Next-generation sequencing, Plaque reduction neutralization test (PRNT), Real Time RT-PCR | 2 (1.3) | 1 (25.0) | 1 (1.4) | | | |
| Complement fixation test | 1 (0.7) | | 1 (1.4) | | | |
| Hemagglutination inhibition test | 1 (0.7) | | 1 (1.4) | | | |
| Indirect ELISA, Classical RT-PCR | 1 (0.7) | | 1 (1.4) | | | |
| **Target detected** | | | | | | |
| Viral RNA | 44 (29.1) | | 10 (14.1) | 32 (49.2) | 2 (28.6) | |

| | | | | | | |
|---|---|---|---|---|---|---|
| Live virus | 34 (22.5) | | 2 (2.8) | 32 (49.2) | | |
| IgM | 32 (21.2) | 1 (25.0) | 30 (42.3) | | 1 (14.3) | |
| Antibodies | 15 (9.9) | | 7 (9.9) | | 4 (57.1) | 4 (100) |
| IgG | 15 (9.9) | | 15 (21.1) | | | |
| IgM, Viral RNA | 10 (6.6) | 3 (75.0) | 7 (9.9) | | | |
| **Infection Status** | | | | | | |
| Current infection | 89 (58.9) | 3 (75.0) | 19 (26.8) | 65 (100) | 2 (28.6) | |
| Past infection | 30 (19.9) | | 22 (31.0) | | 4 (57.1) | 4 (100) |
| Recent infection | 32 (21.2) | 1 (25.0) | 30 (42.3) | | 1 (14.3) | |
| **Sample types** | | | | | | |
| Dried blood spots | 1 (0.7) | | 1 (1.4) | | | |
| Mosquitoes | 65 (43.1) | | | 65 (100) | | |
| Organ tissue | 2 (1.3) | | | | 2 (28.6) | |
| Serum | 83 (55.0) | 4 (100) | 70 (98.6) | | 5 (71.4) | 4 (100) |
| **Risk of bias** | | | | | | |
| Low risk of bias | 32 (21.2) | | 28 (39.4) | 3 (4.6) | 1 (14.3) | |
| Moderate risk of bias | 119 (78.8) | 4 (100) | 43 (60.6) | 62 (95.4) | 6 (85.7) | 4 (100) |
