# Supplementary material for: Epidemiology of yellow fever virus in humans, arthropods, and non-human primates in sub-Saharan Africa: A systematic review and meta-analysis
Source: PLoS Negl Trop Dis. 2022 Jul 22;16(7):e0010610. doi: 10.1371/journal.pntd.0010610 (PMC9307179; doi:10.1371/journal.pntd.0010610)
Supplement: S7 Table — (PDF) [file pntd.0010610.s007.pdf]

S7 Table: Individual characteristics of included studies

Author, Year of publication	Title	Study Design	Sampling	Setting	Hospitalization	Number of sites	Timing of samples collection	Countries	UNSD Region	Country income level	Study period	Age range	YFV vaccine	Species	Detection assay	Target detected	Infection Status	Sample types	Number tested for YFV	Number with YFV
Agwu, 2016	Entomological assessment of yellow fever-epidemic risk indices in Benue State, Nigeria, 2010-2011.	Cross sectional	Non probabilistic	Community-based	Not applicable (if not in the hospital)	Multicenter	Prospetively	Kenya	Eastern Africa	Lower-middle income economies	2010-2011	Not applicable	Not applicable	Individual Mosquitoes	Classical RT-PCR	Viral RNA	Current infection	Mosquitoes	50	6
Ahmed, 2016	Concomitant outbreaks of yellow fever and hepatitis E virus in Darfur States, Sudan, 2012.	Outbreak	Non probabilistic	Community-based	Not applicable (if not in the hospital)	Multicenter	Prospetively	Sudan	Northern Africa	Low-income economies	Dec/2012	Adults	Unclear/Not reported	Humans	Indirect ELISA	IgM	Recent infection	Serum	27	10
Ahmed, 2016	Concomitant outbreaks of yellow fever and hepatitis E virus in Darfur States, Sudan, 2012.	Outbreak	Non probabilistic	Community-based	Not applicable (if not in the hospital)	Multicenter	Prospetively	Sudan	Northern Africa	Low-income economies	Dec/2012	Adults	Unclear/Not reported	Humans	Indirect ELISA	IgM	Recent infection	Serum	80	37
Akelew, 2022	Aetiologies of acute undifferentiated febrile illness at the emergency ward of the University of Gondar Hospital, Ethiopia.	Cross sectional	Non probabilistic	Hospital-based	Ambulatory	Multicenter	Prospetively	Ethiopia	Eastern Africa	Low-income economies	Unclear/Not reported	Unclear/Not reported	Unclear/Not reported	Humans	Real Time RT-PCR	Viral RNA	Current infection	Serum	200	0
Akoua-Koffi, 2014	Yellow fever and dengue fever serotype 3 viruses cocirculation in Côte d'Ivoire in 2008.	Cross sectional	Non probabilistic	Hospital-based	Unclear/Not reported	Multicenter	Prospetively	Côte d'Ivoire	West Africa	Lower-middle income economies	Jan/2008-Dec/2008	Unclear/Not reported	Unclear/Not reported	Humans	Classical RT-PCR	Viral RNA	Current infection	Serum	182	1
Akoua-Koffi, 2014	Yellow fever and dengue fever serotype 3 viruses cocirculation in Côte d'Ivoire in 2008.	Cross sectional	Non probabilistic	Hospital-based	Unclear/Not reported	Multicenter	Prospetively	Côte d'Ivoire	West Africa	Lower-middle income economies	Jan/2008-Dec/2008	Unclear/Not reported	Unclear/Not reported	Humans	Indirect ELISA	IgM	Recent infection	Serum	185	3
Akoua-Koffi, 2014	Yellow fever and dengue fever serotype 3 viruses cocirculation in Côte d'Ivoire in 2008.	Cross sectional	Non probabilistic	Hospital-based	Unclear/Not reported	Multicenter	Prospetively	Côte d'Ivoire	West Africa	Lower-middle income economies	Jan/2008-Dec/2008	Unclear/Not reported	Unclear/Not reported	Humans	Indirect ELISA	IgM	Recent infection	Serum	505	18
Akoua-Koffi, 2001	Inquiry into a fatal case of yellow fever in Cote d'Ivoire in 1999.	Cross sectional	Non probabilistic	Hospital/community based	Hospitalized	Multicenter	Prospetively	Côte d'Ivoire	West Africa	Lower-middle income economies	Aug/1999	All ages	Unclear/Not reported	Humans	Indirect ELISA	IgG	Past infection	Serum	24	21
Akoua-Koffi, 2001	Inquiry into a fatal case of yellow fever in Cote d'Ivoire in 1999.	Cross sectional	Non probabilistic	Hospital/community based	Hospitalized	Multicenter	Prospetively	Côte d'Ivoire	West Africa	Lower-middle income economies	Aug/1999	All ages	Unclear/Not reported	Humans	Indirect ELISA	IgG	Past infection	Serum	18	18
Akoua-Koffi, 2001	Inquiry into a fatal case of yellow fever in Cote d'Ivoire in 1999.	Cross sectional	Non probabilistic	Hospital/community based	Hospitalized	Multicenter	Prospetively	Côte d'Ivoire	West Africa	Lower-middle income economies	Aug/1999	All ages	Unclear/Not reported	Humans	Indirect ELISA	IgM	Recent infection	Serum	24	1
Akoua-Koffi, 2001	Inquiry into a fatal case of yellow fever in Cote d'Ivoire in 1999.	Cross sectional	Non probabilistic	Hospital/community based	Hospitalized	Multicenter	Prospetively	Côte d'Ivoire	West Africa	Lower-middle income economies	Aug/1999	All ages	Unclear/Not reported	Humans	Indirect ELISA	IgM	Recent infection	Serum	18	0
Akoua-Koffi, 2002	[Detection and management of the yellow fever epidemic in the Ivory Coast, 2001].	Outbreak	Non probabilistic	Community-based	Not applicable (if not in the hospital)	Multicenter	Prospetively	Côte d'Ivoire	West Africa	Lower-middle income economies	Mar/2001-Dec/2001	Unclear/Not reported	Unclear/Not reported	Humans	Classical RT-PCR	Viral RNA	Current infection	Serum	266	2
Akoua-Koffi, 2002	[Detection and management of the yellow fever epidemic in the Ivory Coast, 2001].	Outbreak	Non probabilistic	Community-based	Not applicable (if not in the hospital)	Multicenter	Prospetively	Côte d'Ivoire	West Africa	Lower-middle income economies	Mar/2001-Dec/2001	Unclear/Not reported	Unclear/Not reported	Humans	Plaque reduction neutralization test (PRNT)	Antibodies	Past infection	Serum	605	218
Akoua-Koffi, 2002	[Detection and management of the yellow fever epidemic in the Ivory Coast, 2001].	Outbreak	Non probabilistic	Community-based	Not applicable (if not in the hospital)	Multicenter	Prospetively	Côte d'Ivoire	West Africa	Lower-middle income economies	Mar/2001-Dec/2001	Unclear/Not reported	Unclear/Not reported	Humans	Indirect ELISA	IgM	Recent infection	Serum	163	32
Akoua-Koffi, 2002	[Detection and management of the yellow fever epidemic in the Ivory Coast, 2001].	Outbreak	Non probabilistic	Community-based	Not applicable (if not in the hospital)	Multicenter	Prospetively	Côte d'Ivoire	West Africa	Lower-middle income economies	Mar/2001-Dec/2001	Unclear/Not reported	Unclear/Not reported	Humans	Indirect ELISA	IgM	Recent infection	Serum	266	30
Alhakimi, 2015	Epidemiological, Clinical and Entomological Characteristics of Yellow Fever Outbreak in Darfur 2012.	Cross sectional	Non probabilistic	Hospital-based	Hospitalized	Multicenter	Prospetively	Sudan	Northern Africa	Low-income economies	Oct/2012	All ages	Unclear/Not reported	Humans	Indirect ELISA, Classical RT-PCR	IgM, Viral RNA	Current infection	Serum	134	48
Asebe, 2021	Seroprevalence of Yellow fever, Chikungunya, and Zika virus at a community level in the Gambella Region, South West Ethiopia.	Cross sectional	Probabilistic	Community-based	Ambulatory	Multicenter	Prospetively	Ethiopia	Eastern Africa	Low-income economies	Oct/2018-Jun/2019	Adults	No	Humans	Indirect ELISA	IgG	Past infection	Serum	135	4
Baba, 2021	Assessment of immunity against Yellow Fever virus infections in northeastern Nigeria using three serological assays.	Cross sectional	Non probabilistic	Hospital-based	Ambulatory	Multicenter	Prospetively	Nigeria	West Africa	Lower-middle income economies	Unclear/Not reported	All ages	Yes/No	Humans	Indirect ELISA	IgM	Recent infection	Serum	600	255
Babaniyi, 2015	Risk assessment for yellow Fever in Western and north-Western provinces of zambia.	Cross sectional	Non probabilistic	Community-based	Not applicable (if not in the hospital)	Multicenter	Prospetively	Zambia	Eastern Africa	Lower-middle income economies	2010	All ages	No	Humans	Indirect ELISA	IgG	Past infection	Serum	3624	12
Babaniyi, 2015	Risk assessment for yellow Fever in Western and north-Western provinces of zambia.	Cross sectional	Non probabilistic	Community-based	Not applicable (if not in the hospital)	Multicenter	Prospetively	Zambia	Eastern Africa	Lower-middle income economies	2010	All ages	No	Humans	Indirect ELISA	IgM	Recent infection	Serum	3630	6
Caux, 2020	Real-time Metagenomic Analysis of Undiagnosed Fever Cases Unveils a Yellow Fever Outbreak in Edo State, Nigeria.	Cross sectional	Non probabilistic	Hospital-based	Hospitalized	Monocenter	Prospetively	Nigeria	West Africa	Lower-middle income economies	2018	All ages	Unclear/Not reported	Humans	Real Time RT-PCR	Viral RNA	Current infection	Serum	50	29
Chepkorir, 2019	Serological evidence of Flavivirus circulation in human populations in Northern Kenya: an assessment of disease risk 2016-2017.	Cross sectional	Probabilistic	Community-based	Not applicable (if not in the hospital)	Multicenter	Prospetively	Kenya	Eastern Africa	Lower-middle income economies	Feb/2016-Aug/2017	Adults	Yes/No	Humans	Plaque reduction neutralization test (PRNT)	Antibodies	Past infection	Serum	877	44
Diagne, 2021	Yellow Fever Outbreak in Eastern Senegal, 2020-2021.	Cross sectional	Non probabilistic	Community-based	Ambulatory	Multicenter	Prospetively	Senegal	West Africa	Lower-middle income economies	Nov/2020-May/2021	Unclear/Not reported	No	Humans	Plaque reduction neutralization test (PRNT)	IgM	Recent infection	Serum	546	20
Diagne, 2021	Yellow Fever Outbreak in Eastern Senegal, 2020-2021.	Cross sectional	Non probabilistic	Community-based	Not applicable (if not in the hospital)	Multicenter	Prospetively	Senegal	West Africa	Lower-middle income economies	Nov/2020-May/2021	Not applicable	Not applicable	Individual Mosquitoes	Culture	Live virus	Current infection	Mosquitoes	1158	0
Diagne, 2021	Yellow Fever Outbreak in Eastern Senegal, 2020-2021.	Cross sectional	Non probabilistic	Community-based	Not applicable (if not in the hospital)	Multicenter	Prospetively	Senegal	West Africa	Lower-middle income economies	Nov/2020-May/2021	Not applicable	Not applicable	Pooled population	Culture		Current infection	Mosquitoes		
Diallo, 2020	Mobile Laboratory Reveals the Circulation of Dengue Virus Serotype I of Asian Origin in Medina Gounass (Guediawaye), Senegal.	Cross sectional	Non probabilistic	Hospital-based	Ambulatory	Monocenter	Prospetively	Senegal	West Africa	Lower-middle income economies	Sep/2015-Mar/2016	Children	Unclear/Not reported	Humans	Real Time RT-PCR	Viral RNA	Current infection	Serum	104	0
Diallo, 2020	Concurrent amplification of Zika, chikungunya, and yellow fever virus in a sylvatic focus of arboviruses in Southeastern Senegal, 2015.	Cross sectional	Non probabilistic	Community-based	Not applicable (if not in the hospital)	Multicenter	Prospetively	Senegal	West Africa	Lower-middle income economies	Jul/2015-Dec/2015	Unclear/Not reported	Unclear/Not reported	Humans	Indirect ELISA	IgM	Recent infection	Serum	47	0
Diallo, 2020	Concurrent amplification of Zika, chikungunya, and yellow fever virus in a sylvatic focus of arboviruses in Southeastern Senegal, 2015.	Cross sectional	Non probabilistic	Community-based	Not applicable (if not in the hospital)	Multicenter	Prospetively	Senegal	West Africa	Lower-middle income economies	Jul/2015-Dec/2015	Not applicable	Not applicable	Individual Mosquitoes	Culture	Live virus	Current infection	Mosquitoes	2823	8
Diallo, 2020	Concurrent amplification of Zika, chikungunya, and yellow fever virus in a sylvatic focus of arboviruses in Southeastern Senegal, 2015.	Cross sectional	Non probabilistic	Community-based	Not applicable (if not in the hospital)	Multicenter	Prospetively	Senegal	West Africa	Lower-middle income economies	Jul/2015-Dec/2015	Not applicable	Not applicable	Individual Mosquitoes	Culture	Live virus	Current infection	Mosquitoes	1107	7
Diallo, 2020	Concurrent amplification of Zika, chikungunya, and yellow fever virus in a sylvatic focus of arboviruses in Southeastern Senegal, 2015.	Cross sectional	Non probabilistic	Community-based	Not applicable (if not in the hospital)	Multicenter	Prospetively	Senegal	West Africa	Lower-middle income economies	Jul/2015-Dec/2015	Not applicable	Not applicable	Individual Mosquitoes	Culture	Live virus	Current infection	Mosquitoes	106	4
Diallo, 2020	Concurrent amplification of Zika, chikungunya, and yellow fever virus in a sylvatic focus of arboviruses in Southeastern Senegal, 2015.	Cross sectional	Non probabilistic	Community-based	Not applicable (if not in the hospital)	Multicenter	Prospetively	Senegal	West Africa	Lower-middle income economies	Jul/2015-Dec/2015	Not applicable	Not applicable	Individual Mosquitoes	Culture	Live virus	Current infection	Mosquitoes	2034	4
Diallo, 2020	Concurrent amplification of Zika, chikungunya, and yellow fever virus in a sylvatic focus of arboviruses in Southeastern Senegal, 2015.	Cross sectional	Non probabilistic	Community-based	Not applicable (if not in the hospital)	Multicenter	Prospetively	Senegal	West Africa	Lower-middle income economies	Jul/2015-Dec/2015	Not applicable	Not applicable	Individual Mosquitoes	Culture	Live virus	Current infection	Mosquitoes	130	1
Diallo, 2020	Concurrent amplification of Zika, chikungunya, and yellow fever virus in a sylvatic focus of arboviruses in Southeastern Senegal, 2015.	Cross sectional	Non probabilistic	Community-based	Not applicable (if not in the hospital)	Multicenter	Prospetively	Senegal	West Africa	Lower-middle income economies	Jul/2015-Dec/2015	Not applicable	Not applicable	Individual Mosquitoes	Culture	Live virus	Current infection	Mosquitoes	236	0
Diallo, 2020	Concurrent amplification of Zika, chikungunya, and yellow fever virus in a sylvatic focus of arboviruses in Southeastern Senegal, 2015.	Cross sectional	Non probabilistic	Community-based	Not applicable (if not in the hospital)	Multicenter	Prospetively	Senegal	West Africa	Lower-middle income economies	Jul/2015-Dec/2015	Not applicable	Not applicable	Individual Mosquitoes	Culture	Live virus	Current infection	Mosquitoes	264	0
Diallo, 2020	Concurrent amplification of Zika, chikungunya, and yellow fever virus in a sylvatic focus of arboviruses in Southeastern Senegal, 2015.	Cross sectional	Non probabilistic	Community-based	Not applicable (if not in the hospital)	Multicenter	Prospetively	Senegal	West Africa	Lower-middle income economies	Jul/2015-Dec/2015	Not applicable	Not applicable	Individual Mosquitoes	Culture	Live virus	Current infection	Mosquitoes	86	0
Diallo, 2020	Concurrent amplification of Zika, chikungunya, and yellow fever virus in a sylvatic focus of arboviruses in Southeastern Senegal, 2015.	Cross sectional	Non probabilistic	Community-based	Not applicable (if not in the hospital)	Multicenter	Prospetively	Senegal	West Africa	Lower-middle income economies	Jul/2015-Dec/2015	Not applicable	Not applicable	Individual Mosquitoes	Culture	Live virus	Current infection	Mosquitoes	2286	0
Diallo, 2020	Concurrent amplification of Zika, chikungunya, and yellow fever virus in a sylvatic focus of arboviruses in Southeastern Senegal, 2015.	Cross sectional	Non probabilistic	Community-based	Not applicable (if not in the hospital)	Multicenter	Prospetively	Senegal	West Africa	Lower-middle income economies	Jul/2015-Dec/2015	Not applicable	Not applicable	Individual Mosquitoes	Culture	Live virus	Current infection	Mosquitoes	20	0
Diallo, 2020	Concurrent amplification of Zika, chikungunya, and yellow fever virus in a sylvatic focus of arboviruses in Southeastern Senegal, 2015.	Cross sectional	Non probabilistic	Community-based	Not applicable (if not in the hospital)	Multicenter	Prospetively	Senegal	West Africa	Lower-middle income economies	Jul/2015-Dec/2015	Not applicable	Not applicable	Individual Mosquitoes	Culture	Live virus	Current infection	Mosquitoes	45	0
Diallo, 2020	Concurrent amplification of Zika, chikungunya, and yellow fever virus in a sylvatic focus of arboviruses in Southeastern Senegal, 2015.	Cross sectional	Non probabilistic	Community-based	Not applicable (if not in the hospital)	Multicenter	Prospetively	Senegal	West Africa	Lower-middle income economies	Jul/2015-Dec/2015	Not applicable	Not applicable	Individual Mosquitoes	Culture	Live virus	Current infection	Mosquitoes	54	0
Diallo, 2020	Concurrent amplification of Zika, chikungunya, and yellow fever virus in a sylvatic focus of arboviruses in Southeastern Senegal, 2015.	Cross sectional	Non probabilistic	Community-based	Not applicable (if not in the hospital)	Multicenter	Prospetively	Senegal	West Africa	Lower-middle income economies	Jul/2015-Dec/2015	Not applicable	Not applicable	Individual Mosquitoes	Culture	Live virus	Current infection	Mosquitoes	198	0
Diallo, 2020	Concurrent amplification of Zika, chikungunya, and yellow fever virus in a sylvatic focus of arboviruses in Southeastern Senegal, 2015.	Cross sectional	Non probabilistic	Community-based	Not applicable (if not in the hospital)	Multicenter	Prospetively	Senegal	West Africa	Lower-middle income economies	Jul/2015-Dec/2015	Not applicable	Not applicable	Individual Mosquitoes	Culture	Live virus	Current infection	Mosquitoes	49	0
Diallo, 2020	Concurrent amplification of Zika, chikungunya, and yellow fever virus in a sylvatic focus of arboviruses in Southeastern Senegal, 2015.	Cross sectional	Non probabilistic	Community-based	Not applicable (if not in the hospital)	Multicenter	Prospetively	Senegal	West Africa	Lower-middle income economies	Jul/2015-Dec/2015	Not applicable	Not applicable	Individual Mosquitoes	Culture	Live virus	Current infection	Mosquitoes	422	0
Diallo, 2020	Concurrent amplification of Zika, chikungunya, and yellow fever virus in a sylvatic focus of arboviruses in Southeastern Senegal, 2015.	Cross sectional	Non probabilistic	Community-based	Not applicable (if not in the hospital)	Multicenter	Prospetively	Senegal	West Africa	Lower-middle income economies	Jul/2015-Dec/2015	Not applicable	Not applicable	Individual Mosquitoes	Culture	Live virus	Current infection	Mosquitoes	36	0
Diallo, 2020	Concurrent amplification of Zika, chikungunya, and yellow fever virus in a sylvatic focus of arboviruses in Southeastern Senegal, 2015.	Cross sectional	Non probabilistic	Community-based	Not applicable (if not in the hospital)	Multicenter	Prospetively	Senegal	West Africa	Lower-middle income economies	Jul/2015-Dec/2015	Not applicable	Not applicable	Individual Mosquitoes	Culture	Live virus	Current infection	Mosquitoes	74	0
Diallo, 2020	Concurrent amplification of Zika, chikungunya, and yellow fever virus in a sylvatic focus of arboviruses in Southeastern Senegal, 2015.	Cross sectional	Non probabilistic	Community-based	Not applicable (if not in the hospital)	Multicenter	Prospetively	Senegal	West Africa	Lower-middle income economies	Jul/2015-Dec/2015	Not applicable	Not applicable	Individual Mosquitoes	Culture	Live virus	Current infection	Mosquitoes	183	0
Diallo, 2020	Concurrent amplification of Zika, chikungunya, and yellow fever virus in a sylvatic focus of arboviruses in Southeastern Senegal, 2015.	Cross sectional	Non probabilistic	Community-based	Not applicable (if not in the hospital)	Multicenter	Prospetively	Senegal	West Africa	Lower-middle income economies	Jul/2015-Dec/2015	Not applicable	Not applicable	Individual Mosquitoes	Culture	Live virus	Current infection	Mosquitoes	11	0
Diallo, 2020	Concurrent amplification of Zika, chikungunya, and yellow fever virus in a sylvatic focus of arboviruses in Southeastern Senegal, 2015.	Cross sectional	Non probabilistic	Community-based	Not applicable (if not in the hospital)	Multicenter	Prospetively	Senegal	West Africa	Lower-middle income economies	Jul/2015-Dec/2015	Not applicable	Not applicable	Individual Mosquitoes	Culture	Live virus	Current infection	Mosquitoes	20	0
Diallo, 2020	Concurrent amplification of Zika, chikungunya, and yellow fever virus in a sylvatic focus of arboviruses in Southeastern Senegal, 2015.	Cross sectional	Non probabilistic	Community-based	Not applicable (if not in the hospital)	Multicenter	Prospetively	Senegal	West Africa	Lower-middle income economies	Jul/2015-Dec/2015	Not applicable	Not applicable	Individual Mosquitoes	Culture	Live virus	Current infection	Mosquitoes	41	0
Diallo, 2020	Concurrent amplification of Zika, chikungunya, and yellow fever virus in a sylvatic focus of arboviruses in Southeastern Senegal, 2015.	Cross sectional	Non probabilistic	Community-based	Not applicable (if not in the hospital)	Multicenter	Prospetively	Senegal	West Africa	Lower-middle income economies	Jul/2015-Dec/2015	Not applicable	Not applicable	Individual Mosquitoes	Culture	Live virus	Current infection	Mosquitoes	10	0
Diallo, 2020	Concurrent amplification of Zika, chikungunya, and yellow fever virus in a sylvatic focus of arboviruses in Southeastern Senegal, 2015.	Cross sectional	Non probabilistic	Community-based	Not applicable (if not in the hospital)	Multicenter	Prospetively	Senegal	West Africa	Lower-middle income economies	Jul/2015-Dec/2015	Not applicable	Not applicable	Individual Mosquitoes	Culture	Live virus	Current infection	Mosquitoes	19	0
Diallo, 2020	Concurrent amplification of Zika, chikungunya, and yellow fever virus in a sylvatic focus of arboviruses in Southeastern Senegal, 2015.	Cross sectional	Non probabilistic	Community-based	Not applicable (if not in the hospital)	Multicenter	Prospetively	Senegal	West Africa	Lower-middle income economies	Jul/2015-Dec/2015	Not applicable	Not applicable	Individual Mosquitoes	Culture	Live virus	Current infection	Mosquitoes	45	0
Diallo, 2020	Concurrent amplification of Zika, chikungunya, and yellow fever virus in a sylvatic focus of arboviruses in Southeastern Senegal, 2015.	Cross sectional	Non probabilistic	Community-based	Not applicable (if not in the hospital)	Multicenter	Prospetively	Senegal	West Africa	Lower-middle income economies	Jul/2015-Dec/2015	Not applicable	Not applicable	Individual Mosquitoes	Culture	Live virus	Current infection	Mosquitoes	40	0
Diallo, 2020	Concurrent amplification of Zika, chikungunya, and yellow fever virus in a sylvatic focus of arboviruses in Southeastern Senegal, 2015.	Cross sectional	Non probabilistic	Community-based	Not applicable (if not in the hospital)	Multicenter	Prospetively	Senegal	West Africa	Lower-middle income economies	Jul/2015-Dec/2015	Not applicable	Not applicable	Individual Mosquitoes	Culture	Live virus	Current infection	Mosquitoes	13	0
Diallo, 2020	Concurrent amplification of Zika, chikungunya, and yellow fever virus in a sylvatic focus of arboviruses in Southeastern Senegal, 2015.	Cross sectional	Non probabilistic	Community-based	Not applicable (if not in the hospital)	Multicenter	Prospetively	Senegal	West Africa	Lower-middle income economies	Jul/2015-Dec/2015	Not applicable	Not applicable	Individual Mosquitoes	Culture	Live virus	Current infection	Mosquitoes	17	0
Diallo, 2020	Concurrent amplification of Zika, chikungunya, and yellow fever virus in a sylvatic focus of arboviruses in Southeastern Senegal, 2015.	Cross sectional	Non probabilistic	Community-based	Not applicable (if not in the hospital)	Multicenter	Prospetively	Senegal	West Africa	Lower-middle income economies	Jul/2015-Dec/2015	Not applicable	Not applicable	Individual Mosquitoes	Culture	Live virus	Current infection	Mosquitoes	30	0
Diallo, 2020	Concurrent amplification of Zika, chikungunya, and yellow fever virus in a sylvatic focus of arboviruses in Southeastern Senegal, 2015.	Cross sectional	Non probabilistic	Community-based	Not applicable (if not in the hospital)	Multicenter	Prospetively	Senegal	West Africa	Lower-middle income economies	Jul/2015-Dec/2015	Not applicable	Not applicable	Individual Mosquitoes	Culture	Live virus	Current infection	Mosquitoes	98	0
Diallo, 2020	Concurrent amplification of Zika, chikungunya, and yellow fever virus in a sylvatic focus of arboviruses in Southeastern Senegal, 2015.	Cross sectional	Non probabilistic	Community-based	Not applicable (if not in the hospital)	Multicenter	Prospetively	Senegal	West Africa	Lower-middle income economies	Jul/2015-Dec/2015	Not applicable	Not applicable	Individual Mosquitoes	Culture	Live virus	Current infection	Mosquitoes	48	0
Diallo, 2020	Concurrent amplification of Zika, chikungunya, and yellow fever virus in a sylvatic focus of arboviruses in Southeastern Senegal, 2015.	Cross sectional	Non probabilistic	Community-based	Not applicable (if not in the hospital)	Multicenter	Prospetively	Senegal	West Africa	Lower-middle income economies	Jul/2015-Dec/2015	Not applicable	Not applicable	Individual Mosquitoes	Culture	Live virus	Current infection	Mosquitoes	182	0
Diallo, 2014	Patterns of a sylvatic yellow fever virus amplification in southeastern Senegal, 2010.	Cross sectional	Non probabilistic	Community-based	Not applicable (if not in the hospital)	Monocenter	Prospetively	Senegal	West Africa	Lower-middle income economies	May/2010-Mar/2011	Not applicable	Not applicable	Pooled population	Real Time RT-PCR	Viral RNA	Current infection	Mosquitoes		
Ekenna, 2010	Epidemic yellow fever in Borno State of Nigeria: characterisation of hospitalised patients.	Cross sectional	Non probabilistic	Hospital-based	Hospitalized	Multicenter	Prospetively	Nigeria	West Africa	Lower-middle income economies	Aug/1990-Dec/1990	All ages	Unclear/Not reported	Humans	Indirect ELISA	IgM	Recent infection	Serum	35	16
Endale, 2020	Community-based sero-prevalence of chikungunya and yellow fever in the South Omo Valley of Southern Ethiopia.	Cross sectional	Probabilistic	Community-based	Not applicable (if not in the hospital)	Multicenter	Prospetively	Ethiopia	Eastern Africa	Low-income economies	Fev/2018-Jun/2018	All ages	No	Humans	Indirect ELISA	IgG	Past infection	Serum	313	155
Farnon, 2010	Household-based sero-epidemiologic survey after a yellow fever epidemic, Sudan, 2005.	Cross sectional	Probabilistic	Community-based	Not applicable (if not in the hospital)	Multicenter	Prospetively	Sudan	Northern Africa	Low-income economies	Dec/2005	All ages	Yes/No	Humans	Indirect ELISA	IgM	Recent infection	Serum	87	45
Faye, 2007	[Integrated approach to yellow fever surveillance: pilot study in Senegal in 2003-2004].	Cross sectional	Non probabilistic	Community-based	Not applicable (if not in the hospital)	Multicenter	Prospetively	Senegal	West Africa	Lower-middle income economies	2003-2004	Unclear/Not reported	Unclear/Not reported	Humans	Indirect ELISA	IgM	Recent infection	Serum	342	2
Faye, 2007	[Integrated approach to yellow fever surveillance: pilot study in Senegal in 2003-2004].	Cross sectional	Non probabilistic	Community-based	Not applicable (if not in the hospital)	Multicenter	Prospetively	Senegal	West Africa	Lower-middle income economies	2003-2004	Not applicable	Not applicable	Individual Mosquitoes	Classical RT-PCR	Viral RNA	Current infection	Mosquitoes	161	0
Faye, 2007	[Integrated approach to yellow fever surveillance: pilot study in Senegal in 2003-2004].	Cross sectional	Non probabilistic	Community-based	Not applicable (if not in the hospital)	Multicenter	Prospetively	Senegal	West Africa	Lower-middle income economies	2003-2004	Not applicable	Not applicable	Individual Mosquitoes	Classical RT-PCR	Viral RNA	Current infection	Mosquitoes	184	0
Faye, 2007	[Integrated approach to yellow fever surveillance: pilot study in Senegal in 2003-2004].	Cross sectional	Non probabilistic	Community-based	Not applicable (if not in the hospital)	Multicenter	Prospetively	Senegal	West Africa	Lower-middle income economies	2003-2004	Not applicable	Not applicable	Pooled population	Classical RT-PCR	Viral RNA	Current infection	Mosquitoes		
Fokam, 2010	Silent circulation of arboviruses in Cameroon.	Cross sectional	Non probabilistic	Hospital-based	Ambulatory	Multicenter	Prospetively	Cameroon	Central Africa	Lower-middle income economies	Unclear/Not reported	Unclear/Not reported	Unclear/Not reported	Humans	Complement fixation test	Antibodies	Past infection	Serum	18	1
Fokam, 2010	Silent circulation of arboviruses in Cameroon.	Cross sectional	Non probabilistic	Hospital-based	Ambulatory	Multicenter	Prospetively	Cameroon	Central Africa	Lower-middle income economies	Unclear/Not reported	Unclear/Not reported	Unclear/Not reported	Humans	Hemagglutination inhibition test	Antibodies	Past infection	Serum	79	34
Gould, 2008	An outbreak of yellow fever with concurrent chikungunya virus transmission in South Kordofan, Sudan, 2005.	Cross sectional	Non probabilistic	Community-based	Not applicable (if not in the hospital)	Multicenter	Prospetively	Sudan	Northern Africa	Low-income economies	Sep/2005-Dec/2005	Unclear/Not reported	No	Humans	Indirect ELISA	IgM	Recent infection	Serum	16	3
Gould, 2008	An outbreak of yellow fever with concurrent chikungunya virus transmission in South Kordofan, Sudan, 2005.	Cross sectional	Non probabilistic	Community-based	Not applicable (if not in the hospital)	Multicenter	Prospetively	Sudan	Northern Africa	Low-income economies	Sep/2005-Dec/2005	Unclear/Not reported	No	Humans	Indirect ELISA	IgM	Recent infection	Serum	18	4
Ingelbeen, 2018	Urban yellow fever outbreak-Democratic Republic of the Congo, 2016: Towards more rapid case detection.	Cross sectional	Non probabilistic	Hospital-based	Ambulatory	Multicenter	Prospetively	Democratic Republic of the Congo	Central Africa	Low-income economies	Jan/2016-Aug/2016	Unclear/Not reported	Unclear/Not reported	Humans	Indirect ELISA, Plaque reduction neutralization test (PRNT), Classical RT-PCR	IgM, Viral RNA	Current infection	Serum	78	18
Ingelbeen, 2018	Urban yellow fever outbreak-Democratic Republic of the Congo, 2016: Towards more rapid case detection.	Cross sectional	Non probabilistic	Hospital-based	Ambulatory	Multicenter	Prospetively	Democratic Republic of the Congo	Central Africa	Low-income economies	Jan/2016-Aug/2016	Unclear/Not reported	Unclear/Not reported	Humans	Indirect ELISA, Plaque reduction neutralization test (PRNT), Classical RT-PCR	IgM, Viral RNA	Current infection	Serum	2025	78
Inziani, 2020	Seroprevalence of yellow fever, dengue, West Nile and chikungunya viruses in children in Teso South Sub-County, Western Kenya.	Cross sectional	Non probabilistic	Hospital-based	Ambulatory	Multicenter	Prospetively	Kenya	Eastern Africa	Lower-middle income economies	Aug/2010-Feb/2011	Children	Yes/No	Humans	Indirect ELISA	Antibodies	Past infection	Serum	656	29
Jentes, 2010	Acute arboviral infections in Guinea, West Africa, 2006.	Cross sectional	Non probabilistic	Hospital-based	Ambulatory	Multicenter	Prospetively	Guinea	West Africa	Low-income economies	Aug/2006-Jan/2007	Unclear/Not reported	Unclear/Not reported	Humans	Indirect ELISA	IgM	Recent infection	Serum	47	0
Kading, 2013	Prevalence of antibodies to alphaviruses and flaviviruses in free-ranging game animals and nonhuman primates in the greater Congo basin.	Cross sectional	Non probabilistic	Community-based	Not applicable (if not in the hospital)	Multicenter	Retrospectively	Gabon	Central Africa	Upper-middle-income economies	1991-2009	Not applicable	Not applicable	Non-human primates	Plaque reduction neutralization test (PRNT)	Antibodies	Past infection	Serum	25	12
Kading, 2013	Prevalence of antibodies to alphaviruses and flaviviruses in free-ranging game animals and nonhuman primates in the greater Congo basin.	Cross sectional	Non probabilistic	Community-based	Not applicable (if not in the hospital)	Multicenter	Retrospectively	Rwanda	Eastern Africa	Low-income economies	1991-2009	Not applicable	Not applicable	Non-human primates	Plaque reduction neutralization test (PRNT)	Antibodies	Past infection	Serum	22	0
Kading, 2013	Prevalence of antibodies to alphaviruses and flaviviruses in free-ranging game animals and nonhuman primates in the greater Congo basin.	Cross sectional	Non probabilistic	Community-based	Not applicable (if not in the hospital)	Multicenter	Retrospectively	Democratic Republic of the Congo	Central Africa	Low-income economies	1991-2009	Not applicable	Not applicable	Other animal species	Plaque reduction neutralization test (PRNT)	Antibodies	Past infection	Serum	17	0
Kading, 2013	Prevalence of antibodies to alphaviruses and flaviviruses in free-ranging game animals and nonhuman primates in the greater Congo basin.	Cross sectional	Non probabilistic	Community-based	Not applicable (if not in the hospital)	Multicenter	Retrospectively	Democratic Republic of the Congo	Central Africa	Low-income economies	1991-2009	Not applicable	Not applicable	Other animal species	Plaque reduction neutralization test (PRNT)	Antibodies	Past infection	Serum	33	0
Kading, 2013	Prevalence of antibodies to alphaviruses and flaviviruses in free-ranging game animals and nonhuman primates in the greater Congo basin.	Cross sectional	Non probabilistic	Community-based	Not applicable (if not in the hospital)	Multicenter	Retrospectively	Democratic Republic of the Congo	Central Africa	Low-income economies	1991-2009	Not applicable	Not applicable	Other animal species	Plaque reduction neutralization test (PRNT)	Antibodies	Past infection	Serum	21	0
Kading, 2018	Neutralizing antibodies against flaviviruses, Babanki virus, and Rift Valley fever virus in Ugandan bats.	Cross sectional	Non probabilistic	Community-based	Not applicable (if not in the hospital)	Multicenter	Prospetively	Uganda	Eastern Africa	Low-income economies	2011-2013	Not applicable	Not applicable	Other animal species	Plaque reduction neutralization test (PRNT)	Antibodies	Past infection	Serum	625	1
Kayiwa, 2018	Confirmation of Zika virus infection through hospital-based sentinel surveillance of acute febrile illness in Uganda, 2014-2017.	Cross sectional	Non probabilistic	Hospital-based	Ambulatory	Monocenter	Prospetively	Uganda	Eastern Africa	Low-income economies	Feb/2014-Oct/2017,	Unclear/Not reported	Unclear/Not reported	Humans	Indirect ELISA	IgM	Recent infection	Serum	384	2
Konongoi, 2016	Detection of dengue virus serotypes 1, 2 and 3 in selected regions of Kenya: 2011-2014.	Cross sectional	Non probabilistic	Hospital-based	Ambulatory	Multicenter	Prospetively	Kenya	Eastern Africa	Lower-middle income economies	Sep/2011-Dec/2014	Unclear/Not reported	Unclear/Not reported	Humans	Indirect ELISA	IgM	Recent infection	Serum	868	0
Kuniholm, 2016	Seroprevalence and distribution of Flaviviridae, Togaviridae, and Bunyaviridae arboviral infections in rural Cameroonian adults.	Cross sectional	Non probabilistic	Community-based	Not applicable (if not in the hospital)	Multicenter	Prospetively	Cameroon	Central Africa	Lower-middle income economies	2000-2003	Adults	Unclear/Not reported	Humans	Plaque reduction neutralization test (PRNT)	Antibodies	Past infection	Serum	256	69
Kwagonza, 2018	Outbreak of yellow fever in central and southwestern Uganda, February-may 2016.	Outbreak	Non probabilistic	Community-based	Not applicable (if not in the hospital)	Multicenter	Prospetively	Uganda	Eastern Africa	Low-income economies	Jan/2016-Jun/2016	Adults	No	Humans	Real Time RT-PCR	Viral RNA	Current infection	Serum	22	5
Kwagonza, 2018	Outbreak of yellow fever in central and southwestern Uganda, February-may 2016.	Outbreak	Non probabilistic	Community-based	Not applicable (if not in the hospital)	Multicenter	Prospetively	Uganda	Eastern Africa	Low-income economies	Jan/2016-Jun/2016	All ages	No	Humans	Indirect ELISA	IgM	Recent infection	Serum	22	2
Kwagonza, 2018	Outbreak of yellow fever in central and southwestern Uganda, February-may 2016.	Cross sectional	Non probabilistic	Community-based	Not applicable (if not in the hospital)	Multicenter	Prospetively	Uganda	Eastern Africa	Low-income economies	Jan/2016-Jun/2016	Not applicable	Not applicable	Individual Mosquitoes	Real Time RT-PCR	Viral RNA	Current infection	Mosquitoes	3964	0
Kwallah, 2015	Seroprevalence of yellow fever virus in selected health facilities in Western Kenya from 2010 to 2012.	Cross sectional	Non probabilistic	Hospital-based	Unclear/Not reported	Multicenter	Prospetively	Kenya	Eastern Africa	Lower-middle income economies	2010-2012	All ages	Unclear/Not reported	Humans	Indirect ELISA	IgG	Past infection	Serum	469	28
Kwallah, 2015	Seroprevalence of yellow fever virus in selected health facilities in Western Kenya from 2010 to 2012.	Cross sectional	Non probabilistic	Hospital-based	Unclear/Not reported	Multicenter	Prospetively	Kenya	Eastern Africa	Lower-middle income economies	2010-2012	All ages	Unclear/Not reported	Humans	Indirect ELISA	IgM	Recent infection	Serum	469	0
Lilay, 2017	Reemergence of yellow fever in Ethiopia after 50 years, 2013: epidemiological and entomological investigations.	Cross sectional	Non probabilistic	Community-based	Not applicable (if not in the hospital)	Monocenter	Prospetively	Ethiopia	Eastern Africa	Low-income economies	Nov/2012-Oct/2013	All ages	Unclear/Not reported	Humans	Indirect ELISA	IgM	Recent infection	Serum	21	7
Mease, 2011	Seroprevalence and distribution of arboviral infections among rural Kenyan adults: a cross-sectional study.	Cross sectional	Non probabilistic	Community-based	Not applicable (if not in the hospital)	Multicenter	Prospetively	Kenya	Eastern Africa	Lower-middle income economies	Unclear/Not reported	Adults	No	Humans	Indirect ELISA	IgG	Past infection	Serum	1141	104
Mengesha Tsegaye, 2018	Sero-prevalence of yellow fever and related Flavi viruses in Ethiopia: a public health perspective.	Cross sectional	Probabilistic	Community-based	Not applicable (if not in the hospital)	Multicenter	Prospetively	Ethiopia	Eastern Africa	Low-income economies	May/2014-Jul/2014	All ages	No	Humans	Indirect ELISA	IgG	Past infection	Serum	1643	112
Mohamed, 2019	Prevalence and identification of arthropod-transmitted viruses in Kassala state, Eastern Sudan.	Cross sectional	Non probabilistic	Hospital-based	Unclear/Not reported	Monocenter	Prospetively	Sudan	Northern Africa	Low-income economies	Jun/2015-Nov/2015	All ages	Unclear/Not reported	Humans	Real Time RT-PCR	Viral RNA	Current infection	Serum	119	0
Mulchandani, 2019	A community-level investigation following a yellow fever virus outbreak in South Omo Zone, South-West Ethiopia.	Cross sectional	Non probabilistic	Community-based	Not applicable (if not in the hospital)	Multicenter	Prospetively	Ethiopia	Eastern Africa	Low-income economies	Jun/2017-Aug/2017	Not applicable	Not applicable	Individual Mosquitoes	Real Time RT-PCR	Viral RNA	Current infection	Mosquitoes	133	0
Nakounne, 2001	Microbiological surveillance: viral haemorrhagic fevers in the Central African Republic; updated serological data for human beings.	Cross sectional	Non probabilistic	Community-based	Not applicable (if not in the hospital)	Multicenter	Prospetively	Central African Republic	Central Africa	Low-income economies	1994-1997	Unclear/Not reported	Unclear/Not reported	Humans	Enzyme immunoassay	IgG	Past infection	Serum	1762	114
Nakounne, 2001	Microbiological surveillance: viral haemorrhagic fevers in the Central African Republic; updated serological data for human beings.	Cross sectional	Non probabilistic	Community-based	Not applicable (if not in the hospital)	Multicenter	Prospetively	Central African Republic	Central Africa	Low-income economies	1994-1997	Unclear/Not reported	Unclear/Not reported	Humans	Enzyme immunoassay	IgM	Recent infection	Serum	1762	0
Ngoagouni, 2012	Entomological profile of yellow fever epidemics in the Central African Republic, 2006-2010.	Cross sectional	Non probabilistic	Community-based	Not applicable (if not in the hospital)	Multicenter	Prospetively	Central African Republic	Central Africa	Low-income economies	2006-2010	Not applicable	Not applicable	Individual Mosquitoes	Real Time RT-PCR	Viral RNA	Current infection	Mosquitoes	434	0
Ngoagouni, 2012	Entomological profile of yellow fever epidemics in the Central African Republic, 2006-2010.	Cross sectional	Non probabilistic	Community-based	Not applicable (if not in the hospital)	Multicenter	Prospetively	Central African Republic	Central Africa	Low-income economies	2006-2010	Not applicable	Not applicable	Individual Mosquitoes	Real Time RT-PCR	Viral RNA	Current infection	Mosquitoes	777	0
Ngoagouni, 2012	Entomological profile of yellow fever epidemics in the Central African Republic, 2006-2010.	Cross sectional	Non probabilistic	Community-based	Not applicable (if not in the hospital)	Multicenter	Prospetively	Central African Republic	Central Africa	Low-income economies	2006-2010	Not applicable	Not applicable	Individual Mosquitoes	Real Time RT-PCR	Viral RNA	Current infection	Mosquitoes	49	0
Ngoagouni, 2012	Entomological profile of yellow fever epidemics in the Central African Republic, 2006-2010.	Cross sectional	Non probabilistic	Community-based	Not applicable (if not in the hospital)	Multicenter	Prospetively	Central African Republic	Central Africa	Low-income economies	2006-2010	Not applicable	Not applicable	Individual Mosquitoes	Real Time RT-PCR	Viral RNA	Current infection	Mosquitoes	15	0
Ngoagouni, 2012	Entomological profile of yellow fever epidemics in the Central African Republic, 2006-2010.	Cross sectional	Non probabilistic	Community-based	Not applicable (if not in the hospital)	Multicenter	Prospetively	Central African Republic	Central Africa	Low-income economies	2006-2010	Not applicable	Not applicable	Individual Mosquitoes	Real Time RT-PCR	Viral RNA	Current infection	Mosquitoes	352	0
Ngoagouni, 2012	Entomological profile of yellow fever epidemics in the Central African Republic, 2006-2010.	Cross sectional	Non probabilistic	Community-based	Not applicable (if not in the hospital)	Multicenter	Prospetively	Central African Republic	Central Africa	Low-income economies	2006-2010	Not applicable	Not applicable	Individual Mosquitoes	Real Time RT-PCR	Viral RNA	Current infection	Mosquitoes	17	0
Ngoagouni, 2012	Entomological profile of yellow fever epidemics in the Central African Republic, 2006-2010.	Cross sectional	Non probabilistic	Community-based	Not applicable (if not in the hospital)	Multicenter	Prospetively	Central African Republic	Central Africa	Low-income economies	2006-2010	Not applicable	Not applicable	Individual Mosquitoes	Real Time RT-PCR	Viral RNA	Current infection	Mosquitoes	79	0
Ngoagouni, 2012	Entomological profile of yellow fever epidemics in the Central African Republic, 2006-2010.	Cross sectional	Non probabilistic	Community-based	Not applicable (if not in the hospital)	Multicenter	Prospetively	Central African Republic	Central Africa	Low-income economies	2006-2010	Not applicable	Not applicable	Individual Mosquitoes	Real Time RT-PCR	Viral RNA	Current infection	Mosquitoes	16	0
Ngoagouni, 2012	Entomological profile of yellow fever epidemics in the Central African Republic, 2006-2010.	Cross sectional	Non probabilistic	Community-based	Not applicable (if not in the hospital)	Multicenter	Prospetively	Central African Republic	Central Africa	Low-income economies	2006-2010	Not applicable	Not applicable	Individual Mosquitoes	Real Time RT-PCR	Viral RNA	Current infection	Mosquitoes	30	0
Ngoagouni, 2012	Entomological profile of yellow fever epidemics in the Central African Republic, 2006-2010.	Cross sectional	Non probabilistic	Community-based	Not applicable (if not in the hospital)	Multicenter	Prospetively	Central African Republic	Central Africa	Low-income economies	2006-2010	Not applicable	Not applicable	Individual Mosquitoes	Real Time RT-PCR	Viral RNA	Current infection	Mosquitoes	11	0
Ngoagouni, 2012	Entomological profile of yellow fever epidemics in the Central African Republic, 2006-2010.	Cross sectional	Non probabilistic	Community-based	Not applicable (if not in the hospital)	Multicenter	Prospetively	Central African Republic	Central Africa	Low-income economies	2006-2010	Not applicable	Not applicable	Individual Mosquitoes	Real Time RT-PCR	Viral RNA	Current infection	Mosquitoes	981	0
Ngoagouni, 2012	Entomological profile of yellow fever epidemics in the Central African Republic, 2006-2010.	Cross sectional	Non probabilistic	Community-based	Not applicable (if not in the hospital)	Multicenter	Prospetively	Central African Republic	Central Africa	Low-income economies	2006-2010	Not applicable	Not applicable	Individual Mosquitoes	Real Time RT-PCR	Viral RNA	Current infection	Mosquitoes	188	0
Ngoagouni, 2012	Entomological profile of yellow fever epidemics in the Central African Republic, 2006-2010.	Cross sectional	Non probabilistic	Community-based	Not applicable (if not in the hospital)	Multicenter	Prospetively	Central African Republic	Central Africa	Low-income economies	2006-2010	Not applicable	Not applicable	Individual Mosquitoes	Real Time RT-PCR	Viral RNA	Current infection	Mosquitoes	349	0
Ngoagouni, 2012	Entomological profile of yellow fever epidemics in the Central African Republic, 2006-2010.	Cross sectional	Non probabilistic	Community-based	Not applicable (if not in the hospital)	Multicenter	Prospetively	Central African Republic	Central Africa	Low-income economies	2006-2010	Not applicable	Not applicable	Individual Mosquitoes	Real Time RT-PCR	Viral RNA	Current infection	Mosquitoes	109	0
Ngoagouni, 2012	Entomological profile of yellow fever epidemics in the Central African Republic, 2006-2010.	Cross sectional	Non probabilistic	Community-based	Not applicable (if not in the hospital)	Multicenter	Prospetively	Central African Republic	Central Africa	Low-income economies	2006-2010	Not applicable	Not applicable	Individual Mosquitoes	Real Time RT-PCR	Viral RNA	Current infection	Mosquitoes	190	0
Ngoagouni, 2012	Entomological profile of yellow fever epidemics in the Central African Republic, 2006-2010.	Cross sectional	Non probabilistic	Community-based	Not applicable (if not in the hospital)	Multicenter	Prospetively	Central African Republic	Central Africa	Low-income economies	2006-2010	Not applicable	Not applicable	Individual Mosquitoes	Real Time RT-PCR	Viral RNA	Current infection	Mosquitoes	22	0
Ngoagouni, 2012	Entomological profile of yellow fever epidemics in the Central African Republic, 2006-2010.	Cross sectional	Non probabilistic	Community-based	Not applicable (if not in the hospital)	Multicenter	Prospetively	Central African Republic	Central Africa	Low-income economies	2006-2010	Not applicable	Not applicable	Individual Mosquitoes	Real Time RT-PCR	Viral RNA	Current infection	Mosquitoes	50	0
Ngoagouni, 2012	Entomological profile of yellow fever epidemics in the Central African Republic, 2006-2010.	Cross sectional	Non probabilistic	Community-based	Not applicable (if not in the hospital)	Multicenter	Prospetively	Central African Republic	Central Africa	Low-income economies	2006-2010	Not applicable	Not applicable	Individual Mosquitoes	Real Time RT-PCR	Viral RNA	Current infection	Mosquitoes	75	0
Ngoagouni, 2012	Entomological profile of yellow fever epidemics in the Central African Republic, 2006-2010.	Cross sectional	Non probabilistic	Community-based	Not applicable (if not in the hospital)	Multicenter	Prospetively	Central African Republic	Central Africa	Low-income economies	2006-2010	Not applicable	Not applicable	Individual Mosquitoes	Real Time RT-PCR	Viral RNA	Current infection	Mosquitoes	10	0
Ngoagouni, 2012	Entomological profile of yellow fever epidemics in the Central African Republic, 2006-2010.	Cross sectional	Non probabilistic	Community-based	Not applicable (if not in the hospital)	Multicenter	Prospetively	Central African Republic	Central Africa	Low-income economies	2006-2010	Not applicable	Not applicable	Individual Mosquitoes	Real Time RT-PCR	Viral RNA	Current infection	Mosquitoes	110	0
Ngoagouni, 2012	Entomological profile of yellow fever epidemics in the Central African Republic, 2006-2010.	Cross sectional	Non probabilistic	Community-based	Not applicable (if not in the hospital)	Multicenter	Prospetively	Central African Republic	Central Africa	Low-income economies	2006-2010	Not applicable	Not applicable	Individual Mosquitoes	Real Time RT-PCR	Viral RNA	Current infection	Mosquitoes	569	0
Ngoagouni, 2012	Entomological profile of yellow fever epidemics in the Central African Republic, 2006-2010.	Cross sectional	Non probabilistic	Community-based	Not applicable (if not in the hospital)	Multicenter	Prospetively	Central African Republic	Central Africa	Low-income economies	2006-2010	Not applicable	Not applicable	Individual Mosquitoes	Real Time RT-PCR	Viral RNA	Current infection	Mosquitoes	468	0
Ngoagouni, 2012	Entomological profile of yellow fever epidemics in the Central African Republic, 2006-2010.	Cross sectional	Non probabilistic	Community-based	Not applicable (if not in the hospital)	Multicenter	Prospetively	Central African Republic	Central Africa	Low-income economies	2006-2010	Not applicable	Not applicable	Individual Mosquitoes	Real Time RT-PCR	Viral RNA	Current infection	Mosquitoes	55	0
Ngoagouni, 2012	Entomological profile of yellow fever epidemics in the Central African Republic, 2006-2010.	Cross sectional	Non probabilistic	Community-based	Not applicable (if not in the hospital)	Multicenter	Prospetively	Central African Republic	Central Africa	Low-income economies	2006-2010	Not applicable	Not applicable	Individual Mosquitoes	Real Time RT-PCR	Viral RNA	Current infection	Mosquitoes	38	0
Nwachukwu, 2020	The response to re-emergence of yellow fever in Nigeria, 2017.	Outbreak	Non probabilistic	Hospital/community based	Hospitalized/ambulatory	Multicenter	Prospetively	Nigeria	West Africa	Lower-middle income economies	Jul/2016-Oct/2016	All ages	No	Humans	Indirect ELISA, Real Time RT-PCR	IgM, Viral RNA	Current infection	Serum	10	2
Nwachukwu, 2020	The response to re-emergence of yellow fever in Nigeria, 2017.	Outbreak	Non probabilistic	Hospital/community based	Hospitalized/ambulatory	Multicenter	Prospetively	Nigeria	West Africa	Lower-middle income economies	Jul/2016-Oct/2016	All ages	No	Humans	Indirect ELISA, Real Time RT-PCR	IgM, Viral RNA	Current infection	Serum	31	10
Onyango, 2004	Yellow fever outbreak, Imatong, southern Sudan.	Outbreak	Non probabilistic	Community-based	Not applicable (if not in the hospital)	Multicenter	Prospetively	South Sudan	Eastern Africa	Low-income economies	May/2003	All ages	Unclear/Not reported	Humans	Culture	Live virus	Current infection	Serum	28	5
Otshudiema, 2017	Yellow Fever Outbreak - Kongo Central Province, Democratic Republic of the Congo, August 2016.	Outbreak	Non probabilistic	Hospital-based	Hospitalized	Multicenter	Prospetively	Democratic Republic of the Congo	Central Africa	Low-income economies	Jan/2016-Aug/2016	Unclear/Not reported	Yes/No	Humans	Classical RT-PCR	Viral RNA	Current infection	Serum	383	5
Otshudiema, 2017	Yellow Fever Outbreak - Kongo Central Province, Democratic Republic of the Congo, August 2016.	Outbreak	Non probabilistic	Hospital-based	Hospitalized	Multicenter	Prospetively	Democratic Republic of the Congo	Central Africa	Low-income economies	Jan/2016-Aug/2016	Unclear/Not reported	Yes/No	Humans	Indirect ELISA, Plaque reduction neutralization test (PRNT), Classical RT-PCR	IgM, Viral RNA	Current infection	Serum	37	8
Otshudiema, 2017	Yellow Fever Outbreak - Kongo Central Province, Democratic Republic of the Congo, August 2016.	Outbreak	Non probabilistic	Hospital-based	Hospitalized	Multicenter	Prospetively	Democratic Republic of the Congo	Central Africa	Low-income economies	Jan/2016-Aug/2016	Unclear/Not reported	Yes/No	Humans	Indirect ELISA, Plaque reduction neutralization test (PRNT)	IgM	Recent infection	Serum	383	32
Proesmans, 2019	Dengue and chikungunya among outpatients with acute undifferentiated fever in Kinshasa, Democratic Republic of Congo: A cross-sectional study.	Cross sectional	Non probabilistic	Hospital-based	Hospitalized	Monocenter	Prospetively	Democratic Republic of the Congo	Central Africa	Low-income economies	Nov/2015-Jun/2016	All ages	Unclear/Not reported	Humans	Real Time RT-PCR	Viral RNA	Current infection	Serum	227	0
Rachas, 2014	Timeliness of yellow fever surveillance, Central African Republic.	Cross sectional	Non probabilistic	Community-based	Not applicable (if not in the hospital)	Multicenter	Prospetively	Central African Republic	Central Africa	Low-income economies	Jan/2007-Jul/2012	Adults	Yes/No	Humans	Indirect ELISA, Plaque reduction neutralization test (PRNT)	IgM	Recent infection	Serum	3220	55
Rachas, 2014	Timeliness of yellow fever surveillance, Central African Republic.	Cross sectional	Non probabilistic	Community-based	Not applicable (if not in the hospital)	Multicenter	Prospetively	Central African Republic	Central Africa	Low-income economies	Jan/2007-Jul/2012	Adults	Yes/No	Humans	Indirect ELISA, Plaque reduction neutralization test (PRNT)	IgM	Recent infection	Serum	55	11
Rugarabamu, 2021	Seroprevalence and associated risk factors of selected zoonotic viral hemorrhagic fevers in Tanzania.	Cross sectional	Probabilistic	Hospital/community based	Ambulatory	Multicenter	Prospetively	Tanzania	Eastern Africa	Lower-middle income economies	Jun/2018-Nov/2018	All ages	No	Humans	Indirect ELISA	IgG	Past infection	Serum	500	4
Rugarabamu, 2021	Seroprevalence and associated risk factors of selected zoonotic viral hemorrhagic fevers in Tanzania.	Cross sectional	Probabilistic	Hospital/community based	Ambulatory	Multicenter	Prospetively	Tanzania	Eastern Africa	Lower-middle income economies	Jun/2018-Nov/2018	All ages	No	Humans	Indirect ELISA	IgM	Recent infection	Serum	500	3
Schoepp, 2014	Undiagnosed acute viral febrile illnesses, Sierra Leone.	Cross sectional	Non probabilistic	Community-based	Not applicable (if not in the hospital)	Multicenter	Retrospectively	Sierra Leone	West Africa	Low-income economies	Oct/2006-Oct/2008	Unclear/Not reported	Unclear/Not reported	Humans	Indirect ELISA	IgM	Recent infection	Serum	201	5
Simo Tchetgna, 2018	Viral Exploration of Negative Acute Febrile Cases Observed during Chikungunya Outbreaks in Gabon.	Cross sectional	Probabilistic	Hospital-based	Unclear/Not reported	Monocenter	Retrospectively	Gabon	Central Africa	Upper-middle-income economies	2007-2013	Unclear/Not reported	Unclear/Not reported	Humans	Culture	Live virus	Current infection	Serum	436	0
Sow, 2016	Concurrent malaria and arbovirus infections in Kedougou, southeastern Senegal.	Cross sectional	Non probabilistic	Hospital-based	Unclear/Not reported	Multicenter	Prospetively	Senegal	West Africa	Lower-middle income economies	Jul/2009-Marc/2013	All ages	Unclear/Not reported	Humans	Real Time RT-PCR	Viral RNA	Current infection	Serum	13845	2
Sow, 2016	Concurrent malaria and arbovirus infections in Kedougou, southeastern Senegal.	Cross sectional	Non probabilistic	Hospital-based	Unclear/Not reported	Multicenter	Prospetively	Senegal	West Africa	Lower-middle income economies	Jul/2009-Marc/2013	All ages	Unclear/Not reported	Humans	Indirect ELISA	IgM	Recent infection	Serum	13845	11
Staples, 2014	Yellow fever risk assessment in the Central African Republic.	Cross sectional	Probabilistic	Community-based	Not applicable (if not in the hospital)	Multicenter	Prospetively	Central African Republic	Central Africa	Low-income economies	2006; Mar/2009	Unclear/Not reported	No	Humans	Indirect ELISA, Plaque reduction neutralization test (PRNT)	IgG	Past infection	Serum	928	118
Staples, 2014	Yellow fever risk assessment in the Central African Republic.	Cross sectional	Probabilistic	Community-based	Not applicable (if not in the hospital)	Multicenter	Retrospectively	Central African Republic	Central Africa	Low-income economies	2006; Mar/2009	Unclear/Not reported	No	Humans	Indirect ELISA, Plaque reduction neutralization test (PRNT)	Antibodies	Past infection	Serum	549	13
Staples, 2014	Yellow fever risk assessment in the Central African Republic.	Cross sectional	Probabilistic	Community-based	Not applicable (if not in the hospital)	Multicenter	Prospetively	Central African Republic	Central Africa	Low-income economies	2006; Mar/2009	Unclear/Not reported	No	Humans	Indirect ELISA, Plaque reduction neutralization test (PRNT)	IgM	Recent infection	Serum	928	10
Staples, 2014	Yellow fever risk assessment in the Central African Republic.	Cross sectional	Probabilistic	Community-based	Not applicable (if not in the hospital)	Multicenter	Prospetively	Central African Republic	Central Africa	Low-income economies	2006; Mar/2009	Not applicable	Not applicable	Individual Mosquitoes	Real Time RT-PCR	Viral RNA	Current infection	Mosquitoes	243	0
Staples, 2014	Yellow fever risk assessment in the Central African Republic.	Cross sectional	Probabilistic	Community-based	Not applicable (if not in the hospital)	Multicenter	Prospetively	Central African Republic	Central Africa	Low-income economies	2006; Mar/2009	Not applicable	Not applicable	Non-human primates	Indirect ELISA	IgM	Recent infection	Serum	56	1
Sutherland, 2011	Short Report: Serologic Evidence of Arboviral Infections among Humans in Kenya.	Cross sectional	Non probabilistic	Community-based	Not applicable (if not in the hospital)	Multicenter	Retrospectively	Kenya	Eastern Africa	Lower-middle income economies	2000–2004	All ages	Unclear/Not reported	Humans	Indirect immunofluorescence assay	IgG	Past infection	Serum	122	7
Sutherland, 2011	Short Report: Serologic Evidence of Arboviral Infections among Humans in Kenya.	Cross sectional	Non probabilistic	Community-based	Not applicable (if not in the hospital)	Multicenter	Retrospectively	Kenya	Eastern Africa	Lower-middle income economies	2000–2004	All ages	Unclear/Not reported	Humans	Indirect immunofluorescence assay	IgG	Past infection	Serum	419	176
Ushijima, 2021	Surveillance of the major pathogenic arboviruses of public health concern in Gabon, Central Africa: increased risk of West Nile virus and dengue virus infections.	Cross sectional	Non probabilistic	Hospital/community based	Ambulatory	Multicenter	Prospetively	Gabon	Central Africa	Upper-middle-income economies	Nov/2014-Jan/2017; Jan/2015-Mar/2020	All ages	Yes/No	Humans	Indirect ELISA	IgG	Past infection	Serum	462	275
Wamala, 2012	Epidemiological and laboratory characterization of a yellow fever outbreak in northern Uganda, October 2010-January 2011.	Outbreak	Non probabilistic	Community-based	Not applicable (if not in the hospital)	Multicenter	Prospetively	Uganda	Eastern Africa	Low-income economies	Nov/2010-Feb/2011	All ages	Unclear/Not reported	Humans	Next-generation sequencing, Plaque reduction neutralization test (PRNT), Real Time RT-PCR	IgM, Viral RNA	Current infection	Serum	13	7
Wamala, 2012	Epidemiological and laboratory characterization of a yellow fever outbreak in northern Uganda, October 2010-January 2011.	Outbreak	Non probabilistic	Community-based	Not applicable (if not in the hospital)	Multicenter	Prospetively	Uganda	Eastern Africa	Low-income economies	Nov/2010-Feb/2011	All ages	Unclear/Not reported	Humans	Next-generation sequencing, Plaque reduction neutralization test (PRNT), Real Time RT-PCR	IgM, Viral RNA	Current infection	Serum	173	13
Wastika, 2019	Serological evidence of Zika virus infection in non-human primates in Zambia.	Cross sectional	Non probabilistic	Community-based	Not applicable (if not in the hospital)	Monocenter	Retrospectively	Zambia	Eastern Africa	Lower-middle income economies	2009-2010	Not applicable	Not applicable	Non-human primates	Real Time RT-PCR	Viral RNA	Current infection	Organ tissue	48	0
Wastika, 2019	Serological evidence of Zika virus infection in non-human primates in Zambia.	Cross sectional	Non probabilistic	Community-based	Not applicable (if not in the hospital)	Monocenter	Retrospectively	Zambia	Eastern Africa	Lower-middle income economies	2009-2010	Not applicable	Not applicable	Non-human primates	Real Time RT-PCR	Viral RNA	Current infection	Organ tissue	48	0
Wastika, 2019	Serological evidence of Zika virus infection in non-human primates in Zambia.	Cross sectional	Non probabilistic	Community-based	Not applicable (if not in the hospital)	Monocenter	Retrospectively	Zambia	Eastern Africa	Lower-middle income economies	2009-2010	Not applicable	Not applicable	Non-human primates	Plaque reduction neutralization test (PRNT)	Antibodies	Past infection	Serum	48	0
Wastika, 2019	Serological evidence of Zika virus infection in non-human primates in Zambia.	Cross sectional	Non probabilistic	Community-based	Not applicable (if not in the hospital)	Monocenter	Retrospectively	Zambia	Eastern Africa	Lower-middle income economies	2009-2010	Not applicable	Not applicable	Non-human primates	Plaque reduction neutralization test (PRNT)	Antibodies	Past infection	Serum	48	0
Willcox, 2018	Seroepidemiology of Dengue, Zika, and Yellow Fever Viruses among Children in the Democratic Republic of the Congo.	Cross sectional	Probabilistic	Community-based	Not applicable (if not in the hospital)	Monocenter	Retrospectively	Democratic Republic of the Congo	Central Africa	Low-income economies	2013-2014	Children	Unclear/Not reported	Humans	Indirect ELISA	IgG	Past infection	Dried blood spots	978	167
Yaro, 2010	[Epidemiological situation of yellow fever in Burkina Faso from 2003 to 2008].	Cross sectional	Non probabilistic	Community-based	Not applicable (if not in the hospital)	Monocenter	Retrospectively	Burkina Faso	West Africa	Low-income economies	2003-2008	Unclear/Not reported	Unclear/Not reported	Humans	Indirect ELISA, Real Time RT-PCR	IgM, Viral RNA	Current infection	Serum	33	9
Yaro, 2010	[Epidemiological situation of yellow fever in Burkina Faso from 2003 to 2008].	Cross sectional	Non probabilistic	Community-based	Not applicable (if not in the hospital)	Monocenter	Retrospectively	Burkina Faso	West Africa	Low-income economies	2003-2008	Unclear/Not reported	Unclear/Not reported	Humans	Indirect ELISA, Real Time RT-PCR	IgM, Viral RNA	Current infection	Serum	3958	43

Reference

1. Agwu EJ, Igbinosa IB, Isaac C. Entomological assessment of yellow fever-epidemic risk indices in Benue State, Nigeria, 2010-2011. Acta tropica. 2016;161:18-25. Epub 2016/05/18. doi: 10.1016/j.actatropica.2016.05.005. PubMed PMID: 27189925.
2. Ahmed SS, Soghaier MA, Mohammed S, Khogali HS, Osman MM, Abdalla AM. Concomitant outbreaks of yellow fever and hepatitis E virus in Darfur States, Sudan, 2012. Journal of infection in developing countries. 2016;10(1):24-9. Epub 2016/02/02. doi: 10.3855/jidc.6342. PubMed PMID: 26829534.
3. Akelew Y, Pareyn M, Lemma M, Negash M, Bewket G, Derbew A, et al. Aetiologies of acute undifferentiated febrile illness at the emergency ward of the University of Gondar Hospital, Ethiopia. Tropical medicine & international health : TM & IH. 2022. Epub 2022/01/15. doi: 10.1111/tmi.13721. PubMed PMID: 35029010.
4. Akoua-Koffi C, Akran V, kata Faye O, Grandadam M, Ekaza E, Kouassi KS, et al. Yellow fever and dengue fever serotype 3 viruses cocirculation in Côte d'Ivoire in 2008. African Journal of Pathology and Microbiology. 2014.
5. Akoua-Koffi C, Diarrassouba S, Benie VB, Ngbichi JM, Bozoua T, Bosson A, et al. Inquiry into a fatal case of yellow fever in Cote d'lvoire in 1999. Bulletin De La Societe De Pathologie Exotique. 2001;94(3):227-30. PubMed PMID: CCC:000171401500001.
6. Akoua-Koffi C, Ekra KD, Kone AB, Dagnan NS, Akran V, Kouadio KL, et al. [Detection and management of the yellow fever epidemic in the Ivory Coast, 2001]. Medecine tropicale : revue du Corps de sante colonial. 2002;62(3):305-9. Epub 2002/09/25. PubMed PMID: 12244930.
7. Alhakimi HA, Mohamed OG, Khogaly HSE, Arafa KAO, Ahmed WA. Epidemiological, Clinical and Entomological Characteristics of Yellow Fever Outbreak in Darfur 2012. AIMS public health. 2015;2(1):132-41. Epub 2015/03/25. doi: 10.3934/publichealth.2015.1.132. PubMed PMID: 29546100; PubMed Central PMCID: PMCPmc5690374.
8. Asebe G, Michlmayr D, Mamo G, Abegaz WE, Endale A, Medhin G, et al. Seroprevalence of Yellow fever, Chikungunya, and Zika virus at a community level in the Gambella Region, South West Ethiopia. PloS one. 2021;16(7):e0253953. Epub 2021/07/09. doi: 10.1371/journal.pone.0253953. PubMed PMID: 34237098; PubMed Central PMCID: PMCPMC8266044.
9. Baba MM, Yahaya KM, Ezra EM, Adamu M, Kulloma BM, Ikusemoran M, et al. Assessment of immunity against Yellow Fever virus infections in northeastern Nigeria using three serological assays. Journal of medical virology. 2021;93(8):4856-64. Epub 2021/03/31. doi: 10.1002/jmv.26978. PubMed PMID: 33783842.
10. Babaniyi OA, Mwaba P, Mulenga D, Monze M, Songolo P, Mazaba-Liwewe ML, et al. Risk assessment for yellow Fever in Western and north-Western provinces of zambia. Journal of global infectious diseases. 2015;7(1):11-7. Epub 2015/02/28. doi: 10.4103/0974-777x.150884. PubMed PMID: 25722614; PubMed Central PMCID: PMCPmc4338443.
11. Caux C, Etxeberrria I, Teijeira A, Marabelle A, Ajogbasile FV, Oguzie JU, et al. Real-time Metagenomic Analysis of Undiagnosed Fever Cases Unveils a Yellow Fever Outbreak in Edo State, Nigeria. Journal for immunotherapy of cancer. 2020;10(1):3180. Epub 2020/02/28. doi: 10.1136/jitc-2019-000443
10.1038/s41598-020-59880-w. PubMed PMID: 32081931; PubMed Central PMCID: PMCPmc7057427
Pmc7035389.

12. Chepkorir E. Serological evidence of Flavivirus circulation in human populations in Northern Kenya: an assessment of disease risk 2016-2017. Pharmaceutical research. 2019;16(1):65. Epub 2019/05/19. doi: 10.1007/s11095-019-2629-0
10.1186/s12985-019-1176-y. PubMed PMID: 31101058; PubMed Central PMCID: PMCPmc6668022.
13. Diagne MM, Ndione MHD, Gaye A, Barry MA, Diallo D, Diallo A, et al. Yellow Fever Outbreak in Eastern Senegal, 2020-2021. Viruses. 2021;13(8). Epub 2021/08/29. doi: 10.3390/v13081475. PubMed PMID: 34452343; PubMed Central PMCID: PMCPMC8402698.
14. Diallo BI, Bah MB, Yattara F, Keleba RG, MacDonald PDM, Dieng I. Mobile Laboratory Reveals the Circulation of Dengue Virus Serotype I of Asian Origin in Medina Gounass (Guediawaye), Senegal. PloS one. 2020;10(6). Epub 2020/06/26. doi: 10.1371/journal.pone.0234796
10.3390/diagnostics10060408. PubMed PMID: 32560073; PubMed Central PMCID: PMCPmc7316275.
15. Diallo D, Fall G, Diagne CT, Gaye A, Ba Y, Dia I, et al. Concurrent amplification of Zika, chikungunya, and yellow fever virus in a sylvatic focus of arboviruses in Southeastern Senegal, 2015. BMC microbiology. 2020;20(1):181. Epub 2020/07/14. doi: 10.1016/j.vaccine.2020.06.079
10.1186/s12866-020-01866-9. PubMed PMID: 32590939; PubMed Central PMCID: PMCPmc7318437.
16. Diallo D, Sall AA, Diagne CT, Faye O, Hanley KA, Buenemann M, et al. Patterns of a sylvatic yellow fever virus amplification in southeastern Senegal, 2010. The American journal of tropical medicine and hygiene. 2014;90(6):1003-13. Epub 2014/03/13. doi: 10.4269/ajtmh.13-0404. PubMed PMID: 24615140; PubMed Central PMCID: PMCPmc4047721.
17. Ekenna O, Chikwem JO, Mohammed I, Durojaiye SO. Epidemic yellow fever in Borno State of Nigeria: characterisation of hospitalised patients. West African journal of medicine. 2010;29(2):91-7. Epub 2010/06/15. PubMed PMID: 20544633.
18. Endale A. Community-based sero-prevalence of chikungunya and yellow fever in the South Omo Valley of Southern Ethiopia. BMJ open. 2020;14(9):e0008549. Epub 2020/09/05. doi: 10.1136/bmjopen-2020-037903
10.1371/journal.pntd.0008549. PubMed PMID: 32881913; PubMed Central PMCID: PMCPmc7473631.
19. Farnon EC, Gould LH, Griffith KS, Osman MS, Kholy AE, Brair ME, et al. Household-based sero-epidemiologic survey after a yellow fever epidemic, Sudan, 2005. The American journal of tropical medicine and hygiene. 2010;82(6):1146-52. Epub 2010/06/04. doi: 10.4269/ajtmh.2010.09-0105. PubMed PMID: 20519615; PubMed Central PMCID: PMCPmc2877426.
20. Faye O, Diallo M, Dia I, Ba Y, Faye O, Mondo M, et al. [Integrated approach to yellow fever surveillance: pilot study in Senegal in 2003-2004]. Bulletin de la Societe de pathologie exotique (1990). 2007;100(3):187-92. Epub 2007/09/11. PubMed PMID: 17824313.
21. Fokam EB, Levai LD, Guzman H, Amelia PA, Titanji VP, Tesh RB, et al. Silent circulation of arboviruses in Cameroon. East African medical journal. 2010;87(6):262-8. Epub 2010/06/01. doi: 10.4314/eamj.v87i6.63085. PubMed PMID: 23057269.
22. Gould LH, Osman MS, Farnon EC, Griffith KS, Godsey MS, Karch S, et al. An outbreak of yellow fever with concurrent chikungunya virus transmission in South Kordofan, Sudan, 2005. Transactions of the Royal Society of Tropical Medicine and Hygiene. 2008;102(12):1247-54. Epub 2008/05/27. doi: 10.1016/j.trstmh.2008.04.014. PubMed PMID: 18502458.

23. Ingelbeen B, Weregemere NA, Noel H, Tshapenda GP, Mossoko M, Nsio J, et al. Urban yellow fever outbreak-Democratic Republic of the Congo, 2016: Towards more rapid case detection. PLoS neglected tropical diseases. 2018;12(12):e0007029. Epub 2018/12/12. doi: 10.1371/journal.pntd.0007029. PubMed PMID: 30532188; PubMed Central PMCID: PMCPMC6300298.
24. Inziani M, Adungo F, Awando J, Kihoro R, Inoue S, Morita K, et al. Seroprevalence of yellow fever, dengue, West Nile and chikungunya viruses in children in Teso South Sub-County, Western Kenya. International journal of infectious diseases : IJID : official publication of the International Society for Infectious Diseases. 2020;91:104-10. Epub 2019/11/13. doi: 10.4102/phcfm.v11i1.2063
10.1016/j.ijid.2019.11.004. PubMed PMID: 31712089.
25. Jentes ES, Robinson J, Johnson BW, Conde I, Sakouvougui Y, Iverson J, et al. Acute arboviral infections in Guinea, West Africa, 2006. The American journal of tropical medicine and hygiene. 2010;83(2):388-94. Epub 2010/08/05. doi: 10.4269/ajtmh.2010.09-0688. PubMed PMID: 20682888; PubMed Central PMCID: PMCPmc2911191.
26. Kading RC, Borland EM, Cranfield M, Powers AM. Prevalence of antibodies to alphaviruses and flaviviruses in free-ranging game animals and nonhuman primates in the greater Congo basin. Journal of wildlife diseases. 2013;49(3):587-99. Epub 2013/06/20. doi: 10.7589/2012-08-212. PubMed PMID: 23778608.
27. Kading RC, Kityo RM, Mossel EC, Borland EM, Nakayiki T, Nalikka B, et al. Neutralizing antibodies against flaviviruses, Babanki virus, and Rift Valley fever virus in Ugandan bats. PLoS neglected tropical diseases. 2018;8(1):1439215. Epub 2018/03/16. doi: 10.1371/journal.pntd.0006284
10.1080/20008686.2018.1439215. PubMed PMID: 29511459; PubMed Central PMCID: PMCPmc5854243.
28. Kayiwa JT, Nankya AM, Ataliba IJ, Mossel EC, Crabtree MB, Lutwama JJ. Confirmation of Zika virus infection through hospital-based sentinel surveillance of acute febrile illness in Uganda, 2014-2017. Journal of General Virology. 2018;99(9):1248-52. PubMed PMID: CCC:000443388700010.
29. Konongoi L, Ofula V, Nyunja A, Owaka S, Koka H, Makio A, et al. Detection of dengue virus serotypes 1, 2 and 3 in selected regions of Kenya: 2011-2014. Virology journal. 2016;13(1):182. Epub 2016/11/07. doi: 10.1186/s12985-016-0641-0. PubMed PMID: 27814732; PubMed Central PMCID: PMCPmc5097412.
30. Kuniholm MH, Wolfe ND, Huang CY, Mpoudi-Ngole E, Tamoufe U, LeBreton M, et al. Seroprevalence and distribution of Flaviviridae, Togaviridae, and Bunyaviridae arboviral infections in rural Cameroonian adults. The American journal of tropical medicine and hygiene. 2006;74(6):1078-83. Epub 2006/06/09. PubMed PMID: 16760524.
31. Kwagonza L, Masiira B, Kyobe-Bosa H, Kadobera D, Atuheire EB, Lubwama B, et al. Outbreak of yellow fever in central and southwestern Uganda, February-may 2016. BMC infectious diseases. 2018;18(1):548. Epub 2018/11/08. doi: 10.3390/diseases6040099
10.1186/s12879-018-3440-y. PubMed PMID: 30390621; PubMed Central PMCID: PMCPmc6215607.
32. Kwallah A, Inoue S, Thairu-Muigai AW, Kuttoh N, Morita K, Mwau M. Seroprevalence of yellow fever virus in selected health facilities in Western Kenya from 2010 to 2012. Japanese journal of infectious diseases. 2015;68(3):230-4. Epub 2015/02/13. doi: 10.7883/yoken.JJID.2014.288. PubMed PMID: 25672346.

33. Lilay A, Asamene N, Bekele A, Mengesha M, Wendabeku M, Tareke I, et al. Reemergence of yellow fever in Ethiopia after 50 years, 2013: epidemiological and entomological investigations. BMC infectious diseases. 2017;17(1):343. Epub 2017/05/17. doi: 10.1007/s13149-017-0557-y
10.1186/s12879-017-2435-4. PubMed PMID: 28506254; PubMed Central PMCID: PMCPmc5432991.
34. Mease LE, Coldren RL, Musila LA, Prosser T, Ogolla F, Ofula VO, et al. Seroprevalence and distribution of arboviral infections among rural Kenyan adults: a cross-sectional study. Virology journal. 2011;8:371. Epub 2011/07/29. doi: 10.1186/1743-422x-8-371. PubMed PMID: 21794131; PubMed Central PMCID: PMCPmc3161961.
35. Mengesha Tsegaye M, Beyene B, Ayele W, Abebe A, Tareke I, Sall A, et al. Sero-prevalence of yellow fever and related Flavi viruses in Ethiopia: a public health perspective. BMC public health. 2018;18(1):1011. Epub 2018/08/21. doi: 10.1080/17441692.2018.1512144
10.1186/s12889-018-5726-9. PubMed PMID: 30107830; PubMed Central PMCID: PMCPmc6092792.
36. Mohamed N, Magzoub M, Mohamed REH, Aleanizy FS, Alqahtani FY, Nour BYM, et al. Prevalence and identification of arthropod-transmitted viruses in Kassala state, Eastern Sudan. The Libyan journal of medicine. 2019;14(1):1564511-. PubMed PMID: MEDLINE:30716013.
37. Mulchandani R, Massebo F, Bocho F, Jeffries CL, Walker T, Messenger LA. A community-level investigation following a yellow fever virus outbreak in South Omo Zone, South-West Ethiopia. PeerJ. 2019;7:e6466. Epub 2019/02/28. doi: 10.7717/peerj.6466. PubMed PMID: 30809451; PubMed Central PMCID: PMCPMC6387579.
38. Nakounne E, Selekon B, Morvan J. Microbiological surveillance: viral haemorrhagic fevers in the Central African Republic; updated serological data for human beings. Bulletin De La Societe De Pathologie Exotique. 2001;93(5):340-7. PubMed PMID: CCC:000170522100010.
39. Ngoagouni C, Kamgang B, Manirakiza A, Nangouma A, Paupy C, Nakoune E, et al. Entomological profile of yellow fever epidemics in the Central African Republic, 2006-2010. Parasites & vectors. 2012;5:175. Epub 2012/08/18. doi: 10.1186/1756-3305-5-175. PubMed PMID: 22897918; PubMed Central PMCID: PMCPmc3436863.
40. Nwachukwu WE, Yusuff H, Nwangwu U, Okon A, Ogunniyi A, Imuetinyan-Clement J, et al. The response to re-emergence of yellow fever in Nigeria, 2017. International journal of infectious diseases : IJID : official publication of the International Society for Infectious Diseases. 2020;92:189-96. Epub 2020/01/15. doi: 10.1016/j.ijid.2019.12.034. PubMed PMID: 31935537.
41. Onyango CO, Ofula VO, Sang RC, Konongoi SL, Sow A, De Cock KM, et al. Yellow fever outbreak, Imatong, southern Sudan. Emerging infectious diseases. 2004;10(6):1063-8. Epub 2004/06/23. doi: 10.3201/eid1006.030738. PubMed PMID: 15207058; PubMed Central PMCID: PMCPmc3323161.
42. Otshudiema JO, Ndakala NG, Mawanda EK, Tshapenda GP, Kimfuta JM, Nsibu LN, et al. Yellow Fever Outbreak - Kongo Central Province, Democratic Republic of the Congo, August 2016. MMWR Morbidity and mortality weekly report. 2017;66(12):335-8. Epub 2017/04/04 2017/03/31. doi: 10.1016/j.vaccine.2017.03.032
10.15585/mmwr.mm6612a5. PubMed PMID: 28358796; PubMed Central PMCID: PMCPmc5657954.
43. Proesmans S, Katshongo F, Milambu J, Fungula B, Muhindo Mavoko H, Ahuka-Mundeke S, et al. Dengue and chikungunya among outpatients with acute undifferentiated fever

in Kinshasa, Democratic Republic of Congo: A cross-sectional study. 2019;13(9):e0007047. Epub 2019/09/24. doi: 10.1016/s1473-3099(19)30323-8
10.1371/journal.pntd.0007047. PubMed PMID: 31487279; PubMed Central PMCID: PMCPmc6892259.
44. Rachas A, Nakouné E, Bouscaillou J, Paireau J, Selekon B, Senekian D, et al. Timeliness of yellow fever surveillance, Central African Republic. Emerging infectious diseases. 2014;20(6):1004-8. Epub 2014/05/27. doi: 10.3201/eid2006.130671. PubMed PMID: 24857597; PubMed Central PMCID: PMCPmc4036780.
45. Rugarabamu S, Mwanyika GO, Rumisha SF, Sindato C, Lim HY, Misinzo G, et al. Seroprevalence and associated risk factors of selected zoonotic viral hemorrhagic fevers in Tanzania. International journal of infectious diseases : IJID : official publication of the International Society for Infectious Diseases. 2021;109:174-81. Epub 2021/07/10. doi: 10.1016/j.ijid.2021.07.006. PubMed PMID: 34242761.
46. Schoepp RJ, Rossi CA, Khan SH, Goba A, Fair JN. Undiagnosed acute viral febrile illnesses, Sierra Leone. Emerging infectious diseases. 2014;20(7):1176-82. Epub 2014/06/25. doi: 10.3201/eid2007.131265. PubMed PMID: 24959946; PubMed Central PMCID: PMCPmc4073864.
47. Simo Tchetgna H, Sem Ouilibona R, Nkili-Meyong AA, Caron M, Labouba I, Selekon B, et al. Viral Exploration of Negative Acute Febrile Cases Observed during Chikungunya Outbreaks in Gabon. Intervirology. 2018;61(4):174-84. Epub 2019/01/10. doi: 10.1159/000495136. PubMed PMID: 30625488.
48. Sow A, Loucoubar C, Diallo D, Faye O, Ndiaye Y, Senghor CS, et al. Concurrent malaria and arbovirus infections in Kedougou, southeastern Senegal. Malaria journal. 2016;15:47. Epub 2016/01/30. doi: 10.1186/s12936-016-1100-5. PubMed PMID: 26821709; PubMed Central PMCID: PMCPmc4730666.
49. Staples JE, Diallo M, Janusz KB, Manengu C, Lewis RF, Perea W, et al. Yellow fever risk assessment in the Central African Republic. Transactions of the Royal Society of Tropical Medicine and Hygiene. 2014;108(10):608-15. Epub 2014/06/21. doi: 10.1093/trstmh/tru086. PubMed PMID: 24947520; PubMed Central PMCID: PMCPmc4653062.
50. Sutherland LJ, Cash AA, Huang Y-JS, Sang RC, Malhotra I, Moormann AM, et al. Short Report: Serologic Evidence of Arboviral Infections among Humans in Kenya. American Journal of Tropical Medicine and Hygiene. 2011;85(1):158-61. PubMed PMID: CCC:000292433200028.
51. Ushijima Y, Abe H, Nguema Ondo G, Bikangui R, Massinga Loembé M, Zadeh VR, et al. Surveillance of the major pathogenic arboviruses of public health concern in Gabon, Central Africa: increased risk of West Nile virus and dengue virus infections. BMC infectious diseases. 2021;21(1):265. Epub 2021/03/19. doi: 10.1186/s12879-021-05960-9. PubMed PMID: 33731022; PubMed Central PMCID: PMCPMC7966894.
52. Wamala JF, Malimbo M, Okot CL, Atai-Omoruto AD, Tenywa E, Miller JR, et al. Epidemiological and laboratory characterization of a yellow fever outbreak in northern Uganda, October 2010-January 2011. International journal of infectious diseases : IJID : official publication of the International Society for Infectious Diseases. 2012;16(7):e536-42. Epub 2012/05/12. doi: 10.1016/j.ijid.2012.03.004. PubMed PMID: 22575876.
53. Wastika CE, Sasaki M, Yoshii K, Anindita PD, Hang'ombe BM, Mweene AS, et al. Serological evidence of Zika virus infection in non-human primates in Zambia. 2019;164(8):2165-70. doi: 10.1007/s00705-019-04302-0. PubMed PMID: 31154511.
54. Willcox AC, Collins MH, Jadi R, Keeler C, Parr JB, Mumba D, et al. Seroepidemiology of Dengue, Zika, and Yellow Fever Viruses among Children in the Democratic Republic of the

Congo. The American journal of tropical medicine and hygiene. 2018;99(3):756-63. Epub 2018/07/13
2018/07/11. doi: 10.1186/s12889-018-5762-5
10.4269/ajtmh.18-0156. PubMed PMID: 29988000; PubMed Central PMCID: PMCPmc6169194.
55. Yaro S, Zango A, Rouamba J, Diabaté A, Dabiré R, Kambiré C, et al. [Epidemiological situation of yellow fever in Burkina Faso from 2003 to 2008]. Bulletin de la Societe de pathologie exotique (1990). 2010;103(1):44-7. Epub 2010/01/27. doi: 10.1007/s13149-009-0032-5. PubMed PMID: 20101488.
