# Supplementary material for: Epidemiology of yellow fever virus in humans, arthropods, and non-human primates in sub-Saharan Africa: A systematic review and meta-analysis
Source: PLoS Negl Trop Dis. 2022 Jul 22;16(7):e0010610. doi: 10.1371/journal.pntd.0010610 (PMC9307179; doi:10.1371/journal.pntd.0010610)
Supplement: S8 Table — (PDF) [file pntd.0010610.s008.pdf]

S8 Table. Subgroup analyses of case fatality rate and prevalence of yellow fever virus in humans in Africa.

| | Prevalence. % (95%CI) | 95% Prediction interval | N Studies | N Participants | H (95%CI) | I² (95%CI) | P heterogeneity | P difference subtypes |
|---|---|---|---|---|---|---|---|---|
| **YFV case fatality rate in humans** | | | | | | | | |
| **Current infection** | | | | | | | | |
| **Study Design** | | | | | | | | 0.35 |
| Cross sectional | 23.1 [14.3-33.1] | NA | 1 | 78 | NA | NA | 1 | |
| Outbreak | 37.2 [8.5-71.1] | NA | 2 | 23 | 1.6 [1-3.3] | 60.2 [0-90.7] | 0.113 | |
| **Setting** | | | | | | | | 0.032 |
| Community-based | 53.8 [26.1-80.5] | NA | 1 | 13 | NA | NA | 1 | |
| Hospital-based | 23.1 [14.3-33.1] | NA | 1 | 78 | NA | NA | 1 | |
| **Countries** | | | | | | | | 0.094 |
| Democratic Republic of the Congo | 23.1 [14.3-33.1] | NA | 1 | 78 | NA | NA | 1 | |
| Nigeria | 20 [0.5-51.3] | NA | 1 | 10 | NA | NA | 1 | |
| Uganda | 53.8 [26.1-80.5] | NA | 1 | 13 | NA | NA | 1 | |
| **UNSD Region** | | | | | | | | 0.094 |
| Central Africa | 23.1 [14.3-33.1] | NA | 1 | 78 | NA | NA | 1 | |
| Eastern Africa | 53.8 [26.1-80.5] | NA | 1 | 13 | NA | NA | 1 | |
| West Africa | 20 [0.5-51.3] | NA | 1 | 10 | NA | NA | 1 | |
| **Country income level** | | | | | | | | 0.512 |
| Low-income economies | 34.9 [9-66.4] | NA | 2 | 91 | 2.1 [1-4.5] | 78.3 [5.5-95] | 0.032 | |
| Lower-middle income economies | 20 [0.5-51.3] | NA | 1 | 10 | NA | NA | 1 | |
| **YFV prevalence in humans** | | | | | | | | |
| **Current infection** | | | | | | | | |
| **Study Design** | | | | | | | | 0.055 |
| Cross sectional | 3.6 [1.3-6.8] | [0-20.8] | 12 | 21313 | 7.7 [6.8-8.8] | 98.3 [97.8-98.7] | <0.001 | |
| Outbreak | 10.6 [3.6-20.2] | [0-50.5] | 7 | 940 | 3.6 [2.7-4.7] | 92.1 [86.2-95.4] | <0.001 | |
| **Sampling** | | | | | | | | <0.001 |
| Non probabilistic | 6 [3.2-9.6] | [0-25.9] | 18 | 21817 | 6.8 [6.1-7.6] | 97.9 [97.3-98.3] | <0.001 | |
| Probabilistic | 0 [0-0.4] | NA | 1 | 436 | NA | NA | 1 | |
| **Setting** | | | | | | | | 0.196 |
| Community-based | 8 [2.4-16] | [0-42.3] | 6 | 4480 | 3.9 [2.9-5.1] | 93.3 [88-96.2] | <0.001 | |
| Hospital-based | 3.8 [1-8] | [0-27.4] | 12 | 17742 | 7.5 [6.6-8.5] | 98.2 [97.7-98.6] | <0.001 | |
| **Hospitalization** | | | | | | | | 0.039 |

| | Prevalence. % (95%CI) | 95% Prediction interval | N Studies | N Participants | H (95%CI) | I² (95%CI) | P heterogeneity | P difference subtypes |
|---|---|---|---|---|---|---|---|---|
| Ambulatory | 0.7 [0-4.7] | [0-100] | 3 | 2329 | 3.6 [2.3-5.8] | 92.5 [81.3-97] | <0.001 | |
| Hospitalized | 16.7 [1.4-42.4] | [0-100] | 5 | 831 | 7.7 [6.2-9.5] | 98.3 [97.4-98.9] | <0.001 | |
| **Timing of samples collection** | | | | | | | | 0.276 |
| Prospetively | 6.3 [2.7-11.2] | [0-33.9] | 16 | 17826 | 7 [6.2-7.8] | 97.9 [97.4-98.4] | <0.001 | |
| Retrospectively | 2.7 [0-8.7] | [0-100] | 3 | 4427 | 4.4 [2.9-6.6] | 94.8 [88.2-97.7] | <0.001 | |
| **Countries** | | | | | | | | <0.001 |
| Burkina Faso | 9.5 [0-47.9] | NA | 2 | 3991 | 5.2 [3.1-8.7] | 96.3 [89.8-98.7] | <0.001 | |
| Côte d'Ivoire | 0.7 [0.1-1.7] | NA | 2 | 448 | 1 NA | 0 NA | 0.883 | |
| Democratic Republic of the Congo | 2.9 [0.3-7.6] | [0-36.9] | 4 | 2672 | 3.8 [2.6-5.5] | 92.9 [85.1-96.6] | <0.001 | |
| Ethiopia | 0 [0-0.9] | NA | 1 | 200 | NA | NA | 1 | |
| Gabon | 0 [0-0.4] | NA | 1 | 436 | NA | NA | 1 | |
| Nigeria | 45.6 [21.8-70.6] | NA | 2 | 81 | 2.2 [1.1-4.6] | 80.1 [14.4-95.4] | 0.025 | |
| Senegal | 0 [0-0] | NA | 2 | 13949 | 1 NA | 0 NA | 0.47 | |
| South Sudan | 17.9 [5.5-34.5] | NA | 1 | 28 | NA | NA | 1 | |
| Sudan | 11.1 [0-64.3] | NA | 2 | 253 | 9.5 [6.7-13.5] | 98.9 [97.7-99.5] | <0.001 | |
| Uganda | 12.5 [1.3-31] | NA | 2 | 195 | 2 [1-4.3] | 75.9 [0-94.5] | 0.042 | |
| **UNSD Region** | | | | | | | | 0.37 |
| Central Africa | 1.9 [0-5.8] | [0-24.7] | 5 | 3108 | 4.4 [3.3-5.9] | 94.8 [90.7-97.1] | <0.001 | |
| Eastern Africa | 8 [0.2-23] | [0-91.2] | 4 | 423 | 3.7 [2.6-5.4] | 92.9 [85-96.6] | <0.001 | |
| Northern Africa | 11.1 [0-64.3] | NA | 2 | 253 | 9.5 [6.7-13.5] | 98.9 [97.7-99.5] | <0.001 | |
| West Africa | 5.5 [2.2-9.9] | [0-24.7] | 8 | 18469 | 6.8 [5.7-8.1] | 97.8 [97-98.5] | <0.001 | |
| **Country income level** | | | | | | | | <0.001 |
| Low-income economies | 6.1 [2.8-10.4] | [0-26.7] | 12 | 7339 | 5.1 [4.3-6] | 96.1 [94.6-97.2] | <0.001 | |
| Lower-middle income economies | 6.6 [0.6-17.4] | [0-58.5] | 6 | 14478 | 6.3 [5.1-7.8] | 97.5 [96.2-98.4] | <0.001 | |
| Upper-middle-income economies | 0 [0-0.4] | NA | 1 | 436 | NA | NA | 1 | |
| **YFV vaccine** | | | | | | | | 0.172 |
| No | 28.2 [16.6-41.4] | NA | 2 | 53 | 1 NA | 0 NA | 0.472 | |
| Yes/No | 8 [0-37.7] | NA | 2 | 420 | 4.4 [2.5-7.6] | 94.7 [83.9-98.3] | <0.001 | |
| **Study population: Humans** | | | | | | | | <0.001 |
| Febrile patients | 0 [0-0] | [0-0] | 6 | 14931 | 1 [1-2] | 0 [0-74.6] | 0.87 | |
| Mixed human categories | 17.9 [5.5-34.5] | NA | 1 | 28 | NA | NA | 1 | |
| YFV positive case contact | 0.5 [0-2.3] | NA | 1 | 182 | NA | NA | 1 | |
| YFV suspected cases | 13.4 [7.6-20.6] | [0-44.5] | 11 | 7112 | 6.1 [5.3-7.2] | 97.3 [96.4-98] | <0.001 | |
| **Detection assay** | | | | | | | | 0.342 |

| | Prevalence. % (95%CI) | 95% Prediction interval | N Studies | N Participants | H (95%CI) | I² (95%CI) | P heterogeneity | P difference subtypes |
|---|---|---|---|---|---|---|---|---|
| Classical RT-PCR | 0.9 [0.3-1.8] | [0-10.1] | 3 | 831 | 1 [1-3.1] | 0 [0-89.6] | 0.743 | |
| Culture | 4.3 [0-35.9] | NA | 2 | 464 | 4.4 [2.5-7.7] | 94.9 [84.4-98.3] | <0.001 | |
| Real Time RT-PCR | 3.4 [0-10.2] | [0-38.5] | 7 | 14567 | 5.3 [4.3-6.6] | 96.4 [94.5-97.7] | <0.001 | |
| **Target detected** | | | | | | | | 0.756 |
| Live virus | 4.3 [0-35.9] | NA | 2 | 464 | 4.4 [2.5-7.7] | 94.9 [84.4-98.3] | <0.001 | |
| Viral RNA | 2.1 [0.2-5.3] | [0-19.8] | 10 | 15398 | 4.6 [3.8-5.6] | 95.4 [93.2-96.8] | <0.001 | |
| **Past infection** | | | | | | | | |
| **Study Design** | | | | | | | | <0.001 |
| Cross sectional | 18 [11.2-26] | [0-62.4] | 21 | 14973 | 11.3 [10.5-12.2] | 99.2 [99.1-99.3] | <0.001 | |
| Outbreak | 36 [32.3-39.9] | NA | 1 | 605 | NA | NA | 1 | |
| **Sampling** | | | | | | | | 0.016 |
| Non probabilistic | 26.2 [14.3-40.2] | [0-84.7] | 14 | 9655 | 13.4 [12.4-14.5] | 99.4 [99.3-99.5] | <0.001 | |
| Probabilistic | 9.4 [3.8-17.1] | [0-45.1] | 8 | 5923 | 8.7 [7.5-10.1] | 98.7 [98.2-99] | <0.001 | |
| **Setting** | | | | | | | | 0.953 |
| Community-based | 12.9 [6.7-20.9] | [0-53.8] | 14 | 13352 | 12 [11-13.1] | 99.3 [99.2-99.4] | <0.001 | |
| Hospital-based | 12 [3.1-25.1] | [0-83] | 4 | 1222 | 4.9 [3.6-6.7] | 95.8 [92.1-97.8] | <0.001 | |
| **Hospitalization** | | | | | | | | <0.001 |
| Ambulatory | 14.4 [0.4-41.1] | [0-100] | 6 | 1850 | 12.3 [10.7-14.1] | 99.3 [99.1-99.5] | <0.001 | |
| Hospitalized | 95.5 [76.8-100] | NA | 2 | 42 | 1.7 [1-3.6] | 66.7 [0-92.5] | 0.083 | |
| **Timing of samples collection** | | | | | | | | 0.523 |
| Prospetively | 20 [12.1-29.4] | [0-67.7] | 18 | 13510 | 12 [11.1-13] | 99.3 [99.2-99.4] | <0.001 | |
| Retrospectively | 14 [2.2-33.3] | [0-99.7] | 4 | 2068 | 10 [8.2-12.2] | 99 [98.5-99.3] | <0.001 | |
| **Countries** | | | | | | | | <0.001 |
| Cameroon | 26.1 [12-43.1] | [0-100] | 3 | 353 | 2.6 [1.5-4.5] | 85.2 [56.5-95] | 0.001 | |
| Central African Republic | 6.6 [2.4-12.6] | [0-100] | 3 | 3239 | 5.6 [3.9-8] | 96.8 [93.5-98.4] | <0.001 | |
| Côte d'Ivoire | 79.3 [26.7-100] | [0-100] | 3 | 647 | 6 [4.3-8.5] | 97.2 [94.6-98.6] | <0.001 | |
| Democratic Republic of the Congo | 17.1 [14.8-19.5] | NA | 1 | 978 | NA | NA | 1 | |
| Ethiopia | 15.7 [0.1-48] | [0-100] | 3 | 2091 | 12.1 [9.8-15.1] | 99.3 [99-99.6] | <0.001 | |
| Gabon | 59.5 [55-64] | NA | 1 | 462 | NA | NA | 1 | |
| Kenya | 10.1 [3.5-19.5] | [0-53.5] | 6 | 3684 | 8 [6.7-9.6] | 98.4 [97.7-98.9] | <0.001 | |
| Tanzania | 0.8 [0.2-1.8] | NA | 1 | 500 | NA | NA | 1 | |
| Zambia | 0.3 [0.2-0.5] | NA | 1 | 3624 | NA | NA | 1 | |
| **UNSD Region** | | | | | | | | 0.01 |
| Central Africa | 19.1 [8.5-32.7] | [0-73.1] | 8 | 5032 | 10.4 [9.1-11.8] | 99.1 [98.8-99.3] | <0.001 | |

| | Prevalence. % (95%CI) | 95% Prediction interval | N Studies | N Participants | H (95%CI) | I² (95%CI) | P heterogeneity | P difference subtypes |
|---|---|---|---|---|---|---|---|---|
| Eastern Africa | 8.8 [3.3-16.5] | [0-48.3] | 11 | 9899 | 11.2 [10.1-12.4] | 99.2 [99-99.3] | <0.001 | |
| West Africa | 79.3 [26.7-100] | [0-100] | 3 | 647 | 6 [4.3-8.5] | 97.2 [94.6-98.6] | <0.001 | |
| **Country income level** | | | | | | | | <0.001 |
| Low-income economies | 11.5 [5.5-19.4] | [0-46.4] | 7 | 6308 | 8.5 [7.2-10] | 98.6 [98.1-99] | <0.001 | |
| Lower-middle income economies | 20.8 [11-32.5] | [0-73.8] | 14 | 8808 | 11.3 [10.3-12.4] | 99.2 [99.1-99.3] | <0.001 | |
| Upper-middle-income economies | 59.5 [55-64] | NA | 1 | 462 | NA | NA | 1 | |
| **YFV vaccine** | | | | | | | | 0.436 |
| No | 7.3 [2.1-15.4] | [0-46.9] | 8 | 8833 | 11.5 [10.1-12.9] | 99.2 [99-99.4] | <0.001 | |
| Yes/No | 18.1 [0.2-54.7] | [0-100] | 3 | 1995 | 17.6 [14.9-20.8] | 99.7 [99.5-99.8] | <0.001 | |
| **Study population: Humans** | | | | | | | | <0.001 |
| Apparently healthy individuals | 14.1 [7.8-21.8] | [0-54.9] | 16 | 13939 | 11.6 [10.6-12.6] | 99.3 [99.1-99.4] | <0.001 | |
| Febrile patients | 23 [0-65.4] | NA | 2 | 97 | 3.4 [1.8-6.3] | 91.2 [68.9-97.5] | 0.001 | |
| General population | 0.8 [0.2-1.8] | NA | 1 | 500 | NA | NA | 1 | |
| Pregnant women | 42 [37.3-46.8] | NA | 1 | 419 | NA | NA | 1 | |
| YFV positive case contact | 36 [32.3-39.9] | NA | 1 | 605 | NA | NA | 1 | |
| YFV suspected cases | 100 [90.7-100] | NA | 1 | 18 | NA | NA | 1 | |
| **Detection assay** | | | | | | | | <0.001 |
| Complement fixation test | 5.6 [0-22.3] | NA | 1 | 18 | NA | NA | 1 | |
| Enzyme immunoassay | 6.5 [5.4-7.7] | NA | 1 | 1762 | NA | NA | 1 | |
| Hemagglutination inhibition test | 43 [32.3-54.1] | NA | 1 | 79 | NA | NA | 1 | |
| Indirect ELISA | 21.6 [10.9-34.6] | [0-76.8] | 12 | 9963 | 13.5 [12.4-14.7] | 99.5 [99.3-99.5] | <0.001 | |
| Indirect immunofluorescence assay | 21.1 [0-63.9] | NA | 2 | 541 | 8.9 [6.1-12.9] | 98.7 [97.3-99.4] | <0.001 | |
| Plaque reduction neutralization test (PRNT) | 20.6 [3.3-47.4] | [0-100] | 3 | 1738 | 11.6 [9.3-14.5] | 99.3 [98.8-99.5] | <0.001 | |
| **Target detected** | | | | | | | | 0.449 |
| Antibodies | 14.7 [4.8-28.5] | [0-71.6] | 7 | 3040 | 8.9 [7.6-10.4] | 98.7 [98.3-99.1] | <0.001 | |
| IgG | 21 [11.9-31.7] | [0-71.2] | 15 | 12538 | 13 [12-14.1] | 99.4 [99.3-99.5] | <0.001 | |
| **Sample types** | | | | | | | | 0.604 |
| Dried blood spots | 17.1 [14.8-19.5] | NA | 1 | 978 | NA | NA | 1 | |
| Serum | 19 [11.6-27.6] | [0-66.4] | 21 | 14600 | 11.8 [11-12.7] | 99.3 [99.2-99.4] | <0.001 | |
| **Recent infection** | | | | | | | | |
| **Study Design** | | | | | | | | 0.006 |
| Cross sectional | 4.3 [2.1-7.2] | [0-24.1] | 25 | 28353 | 8.2 [7.5-8.9] | 98.5 [98.2-98.7] | <0.001 | |
| Outbreak | 17.5 [8.1-29.4] | [0-67.4] | 5 | 914 | 3.9 [2.8-5.4] | 93.4 [87.5-96.5] | <0.001 | |
| **Sampling** | | | | | | | | 0.622 |

|  | Prevalence. % (95%CI) | 95% Prediction interval | N Studies | N Participants | H (95%CI) | I² (95%CI) | P heterogeneity | P difference subtypes |
|---|---|---|---|---|---|---|---|---|
| Non probabilistic | 5.7 [3.1-9] | [0-28.9] | 27 | 27752 | 8.3 [7.7-9] | 98.6 [98.3-98.8] | <0.001 |  |
| Probabilistic | 9.9 [0.2-30.4] | [0-100] | 3 | 1515 | 9.1 [7-11.8] | 98.8 [97.9-99.3] | <0.001 |  |
| **Setting** |  |  |  |  |  |  |  | 0.328 |
| Community-based | 8.3 [4.7-12.8] | [0-31.9] | 17 | 11404 | 6.5 [5.8-7.3] | 97.6 [97-98.1] | <0.001 |  |
| Hospital-based | 4.7 [0.4-12.7] | [0-48.8] | 10 | 17321 | 11.9 [10.7-13.2] | 99.3 [99.1-99.4] | <0.001 |  |
| **Hospitalization** |  |  |  |  |  |  |  | 0.338 |
| Ambulatory | 3.5 [0-17.1] | [0-76.8] | 6 | 2945 | 12.6 [11-14.4] | 99.4 [99.2-99.5] | <0.001 |  |
| Hospitalized | 10.9 [0.4-30.1] | [0-99.3] | 4 | 460 | 3.2 [2.1-4.8] | 90 [77.4-95.6] | <0.001 |  |
| **Timing of samples collection** |  |  |  |  |  |  |  | 0.029 |
| Prospetively | 6.3 [3.6-9.6] | [0-29.9] | 29 | 29066 | 8.4 [7.8-9.1] | 98.6 [98.4-98.8] | <0.001 |  |
| Retrospectively | 2.5 [0.7-5.2] | NA | 1 | 201 | NA | NA | 1 |  |
| **Countries** |  |  |  |  |  |  |  | <0.001 |
| Central African Republic | 2 [0.2-5.1] | [0-26.9] | 4 | 5965 | 5.7 [4.3-7.6] | 96.9 [94.5-98.3] | <0.001 |  |
| Côte d'Ivoire | 5.8 [1.4-12.4] | [0-35.2] | 6 | 1161 | 3.3 [2.5-4.6] | 91.1 [83.4-95.2] | <0.001 |  |
| Democratic Republic of the Congo | 8.4 [5.8-11.4] | NA | 1 | 383 | NA | NA | 1 |  |
| Ethiopia | 33.3 [14.5-55.1] | NA | 1 | 21 | NA | NA | 1 |  |
| Guinea | 0 [0-3.6] | NA | 1 | 47 | NA | NA | 1 |  |
| Kenya | 0 [0-0.1] | NA | 2 | 1337 | 1 NA | 0 NA | 0.831 |  |
| Nigeria | 42.6 [38.7-46.5] | NA | 2 | 635 | 1 NA | 0 NA | 0.699 |  |
| Senegal | 0.6 [0-3.3] | [0-26.2] | 4 | 14780 | 4.5 [3.2-6.3] | 95.1 [90.5-97.5] | <0.001 |  |
| Sierra Leone | 2.5 [0.7-5.2] | NA | 1 | 201 | NA | NA | 1 |  |
| Sudan | 38.1 [24.5-52.6] | [0-91.9] | 4 | 201 | 1.8 [1.1-3.1] | 70.2 [14.4-89.6] | 0.018 |  |
| Tanzania | 0.6 [0.1-1.5] | NA | 1 | 500 | NA | NA | 1 |  |
| Uganda | 2.4 [0-16.8] | NA | 2 | 406 | 2.3 [1.1-4.8] | 81.8 [23-95.7] | 0.019 |  |
| Zambia | 0.2 [0.1-0.3] | NA | 1 | 3630 | NA | NA | 1 |  |
| **UNSD Region** |  |  |  |  |  |  |  | <0.001 |
| Central Africa | 3.1 [0.7-7] | [0-24.8] | 5 | 6348 | 6.2 [4.9-7.9] | 97.4 [95.8-98.4] | <0.001 |  |
| Eastern Africa | 0.2 [0-1.1] | [0-4.8] | 7 | 5894 | 2.7 [2-3.8] | 86.6 [74.6-93] | <0.001 |  |
| Northern Africa | 38.1 [24.5-52.6] | [0-91.9] | 4 | 201 | 1.8 [1.1-3.1] | 70.2 [14.4-89.6] | 0.018 |  |
| West Africa | 5.8 [0.8-14.1] | [0-54.4] | 14 | 16824 | 10.4 [9.4-11.4] | 99.1 [98.9-99.2] | <0.001 |  |
| **Country income level** |  |  |  |  |  |  |  | 0.077 |
| Low-income economies | 9.3 [4.6-15.2] | [0-38.3] | 14 | 7224 | 6.2 [5.4-7.1] | 97.4 [96.6-98] | <0.001 |  |
| Lower-middle income economies | 4 [1.2-8.2] | [0-30.1] | 16 | 22043 | 9.7 [8.9-10.7] | 98.9 [98.7-99.1] | <0.001 |  |
| **YFV vaccine** |  |  |  |  |  |  |  | 0.043 |

| | Prevalence. % (95%CI) | 95% Prediction interval | N Studies | N Participants | H (95%CI) | I² (95%CI) | P heterogeneity | P difference subtypes |
|---|---|---|---|---|---|---|---|---|
| No | 1.9 [0.2-4.7] | [0-13.7] | 7 | 5660 | 3.7 [2.8-4.8] | 92.6 [87.3-95.7] | <0.001 | |
| Yes/No | 21.2 [3.2-48.7] | [0-100] | 5 | 4345 | 14.2 [12.4-16.3] | 99.5 [99.3-99.6] | <0.001 | |
| **Study population: Humans** | | | | | | | | 0.001 |
| Apparently healthy individuals | 3.5 [0.6-8.1] | [0-24.8] | 7 | 6916 | 6.3 [5.2-7.6] | 97.5 [96.3-98.3] | <0.001 | |
| Febrile patients | 7.9 [0.4-22.6] | [0-76.7] | 6 | 16278 | 15.4 [13.7-17.3] | 99.6 [99.5-99.7] | <0.001 | |
| General population | 0.6 [0.1-1.5] | NA | 1 | 500 | NA | NA | 1 | |
| Mixed human categories | 15.4 [0-45.5] | [0-100] | 3 | 240 | 3.3 [2.1-5.4] | 91 [76.7-96.6] | <0.001 | |
| YFV positive case contact | 8.4 [0-33.3] | NA | 2 | 348 | 6 [3.8-9.6] | 97.3 [93-98.9] | <0.001 | |
| YFV suspected cases | 6 [2.2-11.1] | [0-30.4] | 11 | 4985 | 4.7 [3.9-5.6] | 95.5 [93.5-96.8] | <0.001 | |
| **Detection assay** | | | | | | | | <0.001 |
| Enzyme immunoassay | 0 [0-0.1] | NA | 1 | 1762 | NA | NA | 1 | |
| Indirect ELISA | 7.2 [3.5-12] | [0-38.8] | 24 | 22373 | 8.8 [8.1-9.5] | 98.7 [98.5-98.9] | <0.001 | |
| Plaque reduction neutralization test (PRNT) | 3.7 [2.2-5.4] | NA | 1 | 546 | NA | NA | 1 | |
