# Supplementary figures and images for: Epidemiology of yellow fever virus in humans, arthropods, and non-human primates in sub-Saharan Africa: A systematic review and meta-analysis
Source: PLoS Negl Trop Dis. 2022 Jul 22;16(7):e0010610. doi: 10.1371/journal.pntd.0010610 (PMC9307179; doi:10.1371/journal.pntd.0010610)

S2 Fig. Prevalence estimate of yellow fever virus infections in humans in Africa.

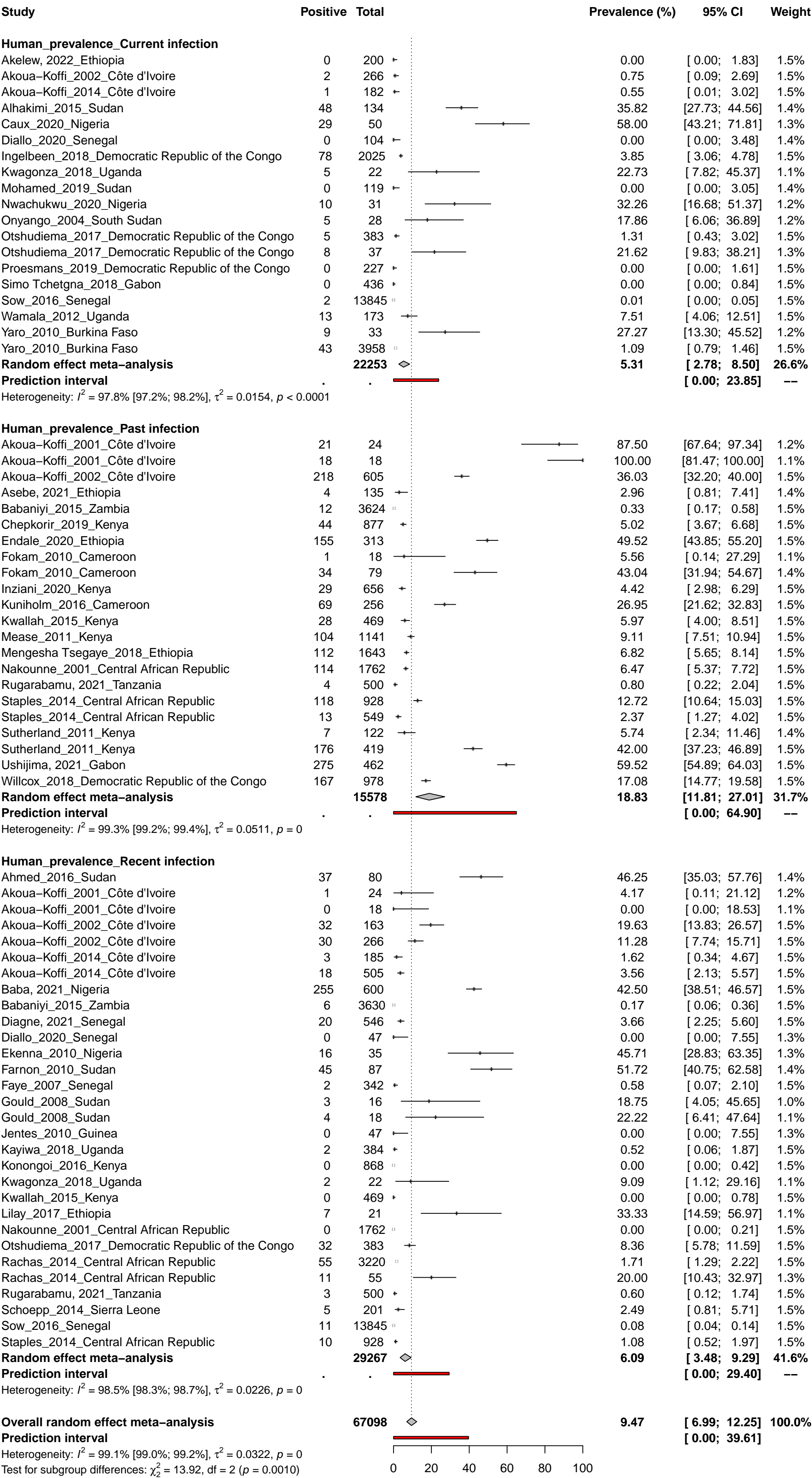

Supplement: S2 Fig — (PDF) [file pntd.0010610.s010.pdf]
