# Supplementary material for: Epidemiology of yellow fever virus in humans, arthropods, and non-human primates in sub-Saharan Africa: A systematic review and meta-analysis
Source: PLoS Negl Trop Dis. 2022 Jul 22;16(7):e0010610. doi: 10.1371/journal.pntd.0010610 (PMC9307179; doi:10.1371/journal.pntd.0010610)
Supplement: S3 Fig — (PDF) [file pntd.0010610.s011.pdf]

S3 Fig. Funnel chart for publications of the yellow fever virus prevalence in humans in Africa.

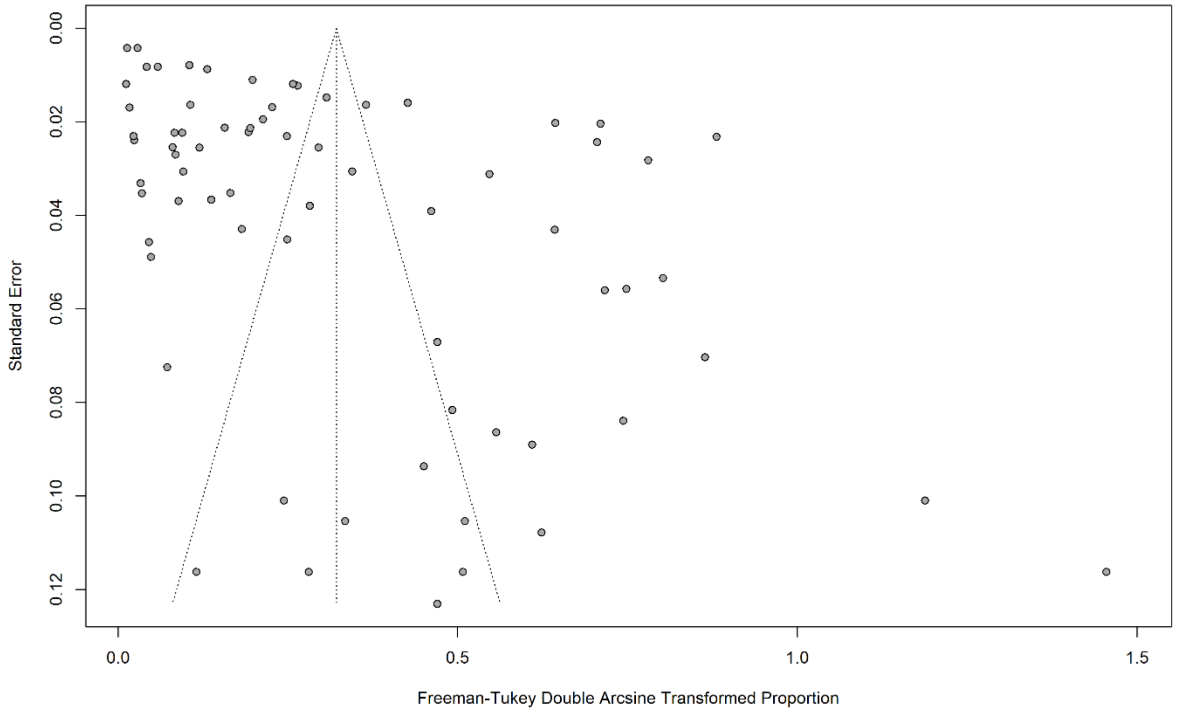

P Egger = < 0.001
