# Supplementary material for: Epidemiology of yellow fever virus in humans, arthropods, and non-human primates in sub-Saharan Africa: A systematic review and meta-analysis
Source: PLoS Negl Trop Dis. 2022 Jul 22;16(7):e0010610. doi: 10.1371/journal.pntd.0010610 (PMC9307179; doi:10.1371/journal.pntd.0010610)
Supplement: Attachment — Submitted filename: Response to Reviewers - PNTD-S-22-00548.doc [file pntd.0010610.s012.doc]

**Review Comments to the Author**

**Editor**

The review and meta-analyzed article were well received by the reviewers; however, some issues require more attention. Additional details are needed in the statistical methods section

Authors: The statistical analysis section has been improved with additional details and the addition of the references, thanks for the suggestion.

The discussion maybe improved by expanding the section that compared the meta-analyzes with recent reports and the section pointing to the limitations of this study

Authors: We did that with the addition of the paragraph below in the discussion section, thank you.

“It should also be noted that African population is more at risk of contracting the yellow fever virus and of developing severe forms and death due to a low rate of vaccination coverage and daily activities that bring them closer to vectors such as agriculture, livestock, hunting, and deforestation [78-81]. Also, the existence in Africa of other health conditions such as malnutrition, tuberculosis, malaria and, HIV are other factors that could be associated with this high of YFV CFR [82-84].”

Fig-2) It would may help to link the maps of Fig-2 with the graphs of Fig-3

Authors: Fig. 2 is the graphical representation of case fatality rate according to African regions while Fig. 3 is the forest plot of case fatality rate estimates, thank you.

Fig-3) Fig-3 is mostly a table, the graph shown is redundant with the data.

Authors: Fig.3 is a forest plot which is a common and widely used way to graphically represent meta-analysis estimates, thank you.

**Reviewer #1:** The objectives of the study are clearly stated.

The systematic review and meta-analysis design adopted in this study is appropriate to address the study objectives.

The study area covered, study population was explicitly described.

Sample size was not necessary for this study, since this was a systematic review of published literature.

The descriptive, and meta-analysis methods were appropriate to analyze the data. Sensitivity and publication analyses were appropriate to address heterogeneity and publication bias.

there are no concerns about ethical requirements. The review reports on previously published data, ethical clearance was not required.

the analysis presented marched the analysis plan

Risk of bias: was well conducted to clearly ascertain the quality of included studies.

Baseline description: the summary and individual data of the included studies was clearly presented as stated in the analysis plan

Random effect model: clearly done to estimate the CFR of YFV and the prevalence of YFV among humans and the results presented in forest plot

Cochran Q test and the I2 test statistic: results clearly reported as described in the analysis plan

Sensitivity analysis: sub-group analysis was conducted as stated in the analysis plan

publication bias and analysis: Funnel plots and Egger's regression test are presented as stated in analysis plan

the results and figures are clearly and completely presented

the figures are of sufficient quality, well labeled and clear

All the conclusions are supported by the data presented

Limitations of the analysis are clearly stated (L356-L359)

Detailed discussion of how the data advances our understanding of the topic is clearly elaborated.

the public health relevance of the study is addressed

The data was clearly presented in this article and there are modifications am suggesting to the authors.

Authors: Thank you very much for your appreciation and comments.

**Reviewer #2:** 1)- The objectives of the study were generally articulated

2)- In general the section Mthods needs further imporvements. For exemple, the authors should:

a) describe in depth the statistical method of the meta-analysis (ex. fixed effect or rendom effect estimation).

Authors: The statistical analysis section has been improved with additional details and the addition of the references, thanks for the suggestion.

b) provide sensitvity analysis to show the robustness of the results.

Authors: sensitivity analysis that included only cross-sectional and low risk of bias studies was performed as stated in the statistical analysis of the method section, thank you.

c)- provide the criteria and summary table (or figure) showing the quality of the studies should be given in the manuscript. This help to assess the comparability of the studies before performing the meta-analysis.

Authors: Supporting table for grouped and individual risk of bias among included studies are given in S5 and 6 Tables, thank you.

1)- The results fairly matched with the plan analysis

Authors: Thank you very much for your appreciation

2)- The results should be compared with current results observed in the litterature.

Authors: Thanks for the suggestion which was previously made and now improved in the discussion section.

3)- The quality of figures may be improved.

Authors: Done, thank you.

1)- The conclusions were generally clear.

Authors: Thank you very much for your appreciation

2)- However, beyond the classical limits (heterogeneity, publication bais), the authors may provide others specific limitations of the study

Authors: Apart from these limitations, we mentioned the specific limitations of the study such as the difficulty of differentiating IgG levels due to a natural infection compared to that of vaccination (Lines 324-325). We also mentioned the issue of cross-reactivity of YFV antibodies with that of other flaviviruses in the discussion (Lines 342-343), thank you.

Authors:

**Reviewer #3:** The methodology followed sufficient analysis. Clearly describing the entire methodology.

The analyzes followed the methodological planning and presented very interesting results.

I emphasize that these results are of paramount importance for public health worldwide.

The conclusion is supported by the data presented conclusively. The limitations of the study are clearly presented. This is a very important study for global public health as it is the first review in the region.

I suggest accepting the article without changes.

Authors: Thank you very much for your appreciation and comments.
